# Supplementary material for: Development and validation of a high-throughput qPCR platform for the detection of soil-transmitted helminth infections
Source: PLoS Negl Trop Dis. 2025 Jan 10;19(1):e0012760. doi: 10.1371/journal.pntd.0012760 (PMC11756772; doi:10.1371/journal.pntd.0012760)
Supplement: S1 Appendix — The complete report of all activities performed as part of the DeWorm3 assay validation process. This report summarizes and collates the underlying data generated in support of this manuscript. (PDF) [file pntd.0012760.s001.pdf]

DeWorm3 Assay Validation Report

Table of Contents

Introduction 3

Background 3

I.A. Run Controls Validation 3

I.A.i. Validation of Run Control: *Ascaris suum*-spiked stool....3

I.A.ii. Validation of Run Control: *Bacillus atrophaeus* bacteria....4

I.A.iii. Validation of Run Control: Whole genome-amplified (WGA) STH nucleic acid....5

I.A.iv. Run Control Validation Target Performance Specifications Go/No-Go Chart....5

I.B. Assay Validation....6

I.B.i. Multi-Site Characterization of Spiked-Stool Standards 6

I.B.ii. Evaluation of Assay Accuracy....9

I.B.iii. Evaluation of Assay Specificity....10

I.B.iv. Evaluation of Assay Sensitivity/LOD 11

I.B.v. Evaluation of Assay Precision 13

I.B.vi. Assay Validation Target Performance Specifications Go/No-Go Chart 13

Additional Investigation....14

Conclusion....16

Next Steps 16

Appendices....i

A1: Raw Data: *Ascaris suum*-spiked stool Validation (I.A.i.) i

A2: Raw Data: *B. atrophaeus* Validation (I.A.ii.) ii

A3: Raw Data: WGA STH nucleic acid (I.A.iii.) x

A4: Raw Data: Multi-site Characterization of Spiked Stool Standards, Assay Accuracy ((I.B.i., I.B.ii) xi

A5: Evaluation of Assay Specificity (I.B.iii) xxii

A6: Raw Data: Assay Sensitivity/LOD (I.B.iv)....xxvii

A7: 200 Sample Experiment Data xxxv

Figure A7.1: The 200 sample experiment found 48 positive samples across 4 extractions. Within these samples, there was discordance, both between sites and within sites....xxxvi

Figure A7.2: *All line data for the 200 sample experiment extractions*....xl

Figure A7.3: *DNA line data for N. americanus at Quantigen and Smith* lvi

Figure A7.4: *DNA line data for T. trichiura at Quantigen and Smith* lxiii

Figure A7.5: *DNA line data for A. lumbricoides at Quantigen and Smith*.... lxx

A8: Control Proposal.... lxxvii

Purpose.... lxxvii

Internal Process Control: *Bacillus atrophaeus* lxxvii

Data in support of *B. atrophaeus* Proposal………………………………………………………………………… lxxviii
Supporting Material for the *B. atrophaeus* Proposal……………………………………………………………… lxxx
Extraction Control: *A. suum*………………………………………………………………………………………… lxxxi
Current *A. suum* Control……………………………………………………………………………………………… lxxxi
A. suum Proposal……………………………………………………………………………………………………… lxxxi
Data in support of *A. suum* Proposal………………………………………………………………………………... lxxxii
Supporting Material for the *A. suum* Proposal…………………………………………………………………... lxxxiv
Negative Controls: No Sample Control and No Template Control………………………………………… lxxxv
No Template Control (NTC)………………………………………………………………………………………… lxxxv
NSC/NTC Proposal…………………………………………………………………………………………………….. lxxxv
Data to support NSC/NTC Proposal………………………………………………………………………………… lxxxv
Evidence to support NSC/NTC Proposal…………………………………………………………………………… lxxxviii
Positive PCR…… lxxxviii
Current Positive PCR Standard……………………………………………………………………………………… lxxxviii
Positive PCR Proposal………………………………………………………………………………………………… lxxxviii
Transition to Plasmids………………………………………………………………………………………………... lxxxviii
Continue with WGA, with altered passing qualifications……………………………………………………… lxxxix
Data to Support Positive PCR Proposal…………………………………………………………………………… lxxxix
Evidence to Support Positive PCR Proposal……………………………………………………………………… xc
References……… xci

Introduction

The *DeWorm3 Assay Validation Plan* outlined a novel approach to assay validation in the absence of both standardized Soil Transmitted Helminth (STH) control material and an accurate “gold-standard” for STH diagnostics (I.H). This plan was executed by DeWorm3 collaborators at Smith College, Quantigen LLC, and Christian Medical College Vellore (CMC) from September 2020 through August 2021. The results demonstrate high performance of the DeWorm3 Assay and point to the potential for improved STH diagnostics.

Results presented below are organized by sections of the *DeWorm3 Assay Validation Plan*: Run Control Validation (II.A) and Assay Validation (II.B). Each verification checkpoint outlined in the *DeWorm3 Assay Validation Plan* is denoted with “***Verification***” throughout the report.

Background

STH experts at Smith College developed and characterized (1) run controls and (2) a panel of spiked-stool standards for use in evaluating the DeWorm3 qPCR Assay. Through a generative process of standards development and assay validation, DeWorm3 teams at Quantigen and CMC further characterized these spiked stool standards and run controls. A final panel of 18 well-characterized spiked stool standards representing various species and intensities of STH infection were developed and validated (**Figure 4**). The final panel of standards, in addition to field-collected samples from DeWorm3 collection sites, were utilized to measure target performance specifications of the DeWorm3 Assay (I.B.i – I.B.v). Finally, a large-scale additional investigation was included to resolve any Assay performance concerns.

I.A. Run Controls Validation

I.A.i. Validation of Run Control: *Ascaris suum*-spiked stool

Control Preparation

A. suum-spiked stool will serve as the positive extraction control in the DeWorm3 extraction procedure. A fixed number of *A. suum* eggs were spiked into 5g of commercially purchased, helminth-naïve stool to achieve an eggs-per-gram (epg) concentration indicative of a moderate-level infection (5,000 to 49,999 epg). 280 aliquots (70 mg) of *A. suum*-spiked stool were prepared. Smith College extracted DNA from 10 aliquots and tested by qPCR in duplicate for quality control.

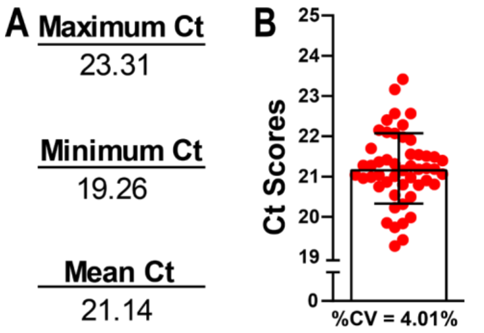

Figure 1: *A. suum* run control validation. A) The minimum, maximum, and average Ct of the validation. B) Dot plot depicting the range of the Ct’s from the assay. The error bar in B is the standard deviation.

Methods Summary

Quantigen extracted DNA from 25 *A. suum*-spiked aliquots and tested them in duplicate by qPCR using DeWorm3 Lyophilized qPCR Plates.

Results

A. suum Ct scores (n=50) generated a composite CV of 4%, meeting the minimum target specification with a mean of 21.14 (± 0.86) (**Figure 1**). The raw data are presented in the Appendix A1 in S1 Appendix (**Table A1. 1**).

Defining Ct Score Cut-off

Passing qualifications for this control are defined as positive *A. suum* amplification with a Ct score of ≤ 23.

I.A.ii. Validation of Run Control: *Bacillus atrophaeus* bacteria

Control Preparation

Bacillus atrophaeus will serve as the internal extraction control spiked into each DeWorm3 sample at a fixed concentration. *B. atrophaeus* stock were commercially purchased and diluted prior to use.

Methods Summary

B. atrophaeus spores were spiked into 200 STH-negative stool samples each from Benin and India[a]. *B. atrophaeus* was validated on both Assay 1 (*B. atrophaeus*, *N. americanus*, *T. trichiura*) and Assay 2 (*B. atrophaeus, A. lumbricoides, A. duodenale*).

Results

Across samples from both Benin and India (n=413)[b] *B. atrophaeus* Ct scores generated a composite CV of < 7% (**Figure 2**). The mean *B. atrophaeus* Ct score for Assay 1 and Assay 2 were 29.96 (±2.02) and 30.00 (±2.00), respectively. The raw data are presented in Appendix A2 in S1 Appendix (**Table A2. 1**.)

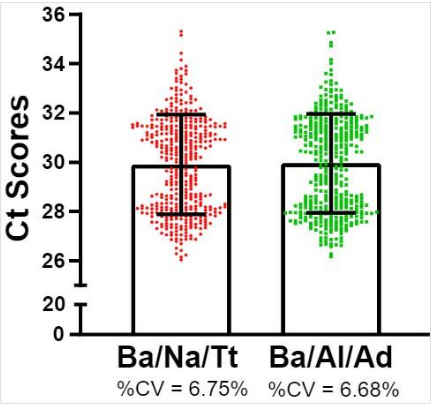

Figure 2: *B. atrophaeus* validation. A dot plot displaying all the Ct scores for Benin and India samples for both assays.

Defining Ct Score Cut-off

The acceptable *B. atrophaeus* Ct score range for the DeWorm3 study will be informed by results from DeWorm3 baseline samples. In the interim, passing qualifications for this control are defined as positive *B. atrophaeus* amplification with a Ct score ≤ 34 in both Assay 1 & Assay 2 in the absence of STH-amplification.

---

[a] STH-negative status was determined by the DeWorm3 Assay as part of evaluation of Assay Specificity (section II.B.iv). DeWorm3 samples collected in the Malawi site are not included in this report due to delays in sample shipment. Once received, *B. atrophaeus*, will be validated in samples from this site.

[b] An additional 13 STH negative stool samples were utilized from Benin for a total of 213 Benin samples.

I.A.iii. Validation of Run Control: Whole genome-amplified (WGA) STH nucleic acid

Control Preparation

WGA STH nucleic acid will serve as a positive PCR control for the DeWorm3 Assay. Purified genomic DNA for each of the four helminth species was subjected to whole-genome amplification (WGA), diluted, and combined into a single pool targeting ±2 Ct's for each species.

Table 1: WGA validation.

Species	Min Ct	Max Ct	Mean Ct	%CV
N. americanus	27.74	31.27	29.67	3.32%
A. lumbricoides	28.97	37.84	32.03	5.90%
A. duodenale	28.26	37.47	29.41	6.21%
T. trichiura	26.94	29.20	27.85	1.80%

Methods Summary

Twenty-five aliquots of the final WGA STH nucleic acid pool were tested by the by qPCR using DeWorm3 Lyophilized qPCR Plates.

Results

Ct scores for each STH species in all aliquots resulted in < 10% CV (**Table 1**). The raw data are presented in the Appendix A3 in S1 Appendix (**Table A3. 1**).

Defining Ct Score Cut-off

Passing qualifications for WGA STH nucleic acid will be defined as < 2 standard deviations from the validation-defined mean Ct for each Assay. Thus, the Ct score cut-off for each species are as follows: *N. americanus* ≤ 31.6, *A. lumbricoides* ≤ 35.8, *A. duodenale* ≤ 33.1, and *T. trichiura* ≤ 28.9.

I.A.iv. Run Control Validation Target Performance Specifications Go/No-Go Chart

Run Controls Validation

	Variance		Results	
Validation	Minimum	Optimistic	Observed	Pass/Fail
Ascaris suum-spiked stool	≤ 10% CV	≤ 2% CV	4.01% CV	Pass
Bacillus atrophaeus	≤ 10% CV	≤ 2% CV	<7% CV	Pass
WGA STH nucleic acid	≤ 10% CV	≤ 2% CV	2-6% CV	Pass

Table 2: Go/No-go table for validation II.A. Section II.A.iv is awaiting *B. atrophaeus* data from Malawi samples, however, all run controls meet the minimum target.

I.B. Assay Validation

Smith College prepared a panel of twenty-five candidate standards containing twenty STH-positive and five STH-negative standards by spiking helminth naïve stool with field samples of known STH infection status (**Figure 3**). Standards were initially characterized by triplicate DNA extraction and quadruplicate qPCR (3 aliquots x 4 replicates), according to Smith College's procedures. All standards maintained consistent amplification across all four qPCR technical replicates tested at Smith College. For assay validation, the STH-status of each standard was defined by the initial characterization by the Smith College Assay.

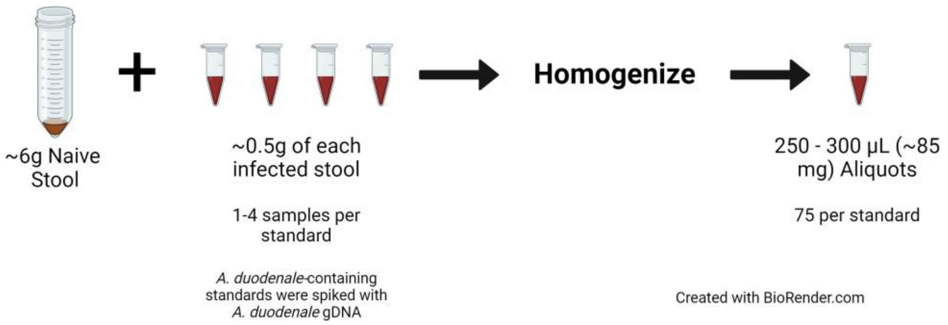

Figure 3*: Smith College procedure for preparation of spiked-stool standards*

I.B.i. Multi-Site Characterization of Spiked-Stool Standards

Methods Summary

Characterization of Initial Panel

Three blinded aliquots of each standard (~85 mg per aliquot) were extracted and qPCR tested in quadruplicate at Quantigen by the DeWorm3 Assay (25 standards x 3 aliquots x 4 technical replicates). In the following summary, technical replicates refer to the 300 qPCR replicates tested at Quantigen (i.e., twelve qPCR data points for each of the twenty-five standards).

Discordant results across (1) technical replicates tested by the DeWorm3 Assay and (2) between aliquots tested by the DeWorm3 Assay and the Smith College Assay were investigated to determine the source of discordance. Experiments interrogated (1) Differences in qPCR Assay (i.e., Smith College Assay vs. DeWorm3 Assay), (2) Differences in DNA extraction procedures (i.e., Smith College DNA Extraction vs. DeWorm3 DNA extraction), and (3) Aliquot-to-aliquot variability of the contrived spiked-stool standards. As outlined in the Validation Plan, multi-site characterization interrogated all standards to ensure a robust panel of standards for utilization in DeWorm3 Assay Validation.

Results

Characterization of Initial Panel

Table 3: Failed Detection of STH in Initial Panel Standards by the DeWorm3 Assay. 3 aliquots x 4 technical replicates (n=12) were tested per standard.

Standard #	STH Target	Aliquot Failures	Technical Replicate Failures
**3**	Tt		1
**4.2**	Tt	1	7
**8**	Tt		1
**9**	Tt		2
**10.2**	Ad		4
**11**	Tt		2
**12**	Na		2
**13**	Tt	2	9
**16**	Tt	1	4
**17**	Na		5
**20**	Tt		2

Quantigen observed dropout of technical replicates in 11 of the 25 standards during initial testing by the DeWorm3 Assay (**Table 3**)**.** The DeWorm3 Assay failed to detect *T. trichiura* in at least 1 of 12 technical replicates for 8 standards (#3, #4.2, #8, #9, #11, #13, #16, #20, **Appendix A4**, **A4.1**) including 3 standards for which Quantigen failed to detect *T. trichiura* for at least 1 of 3 aliquots (Standards #4.2, #13, and #16). The DeWorm3 Assay failed to detect *N. americanus* in at least 1 of 12 technical replicates for 2 standards (#12 and #17) (**Appendix A4, A4.2**), none of which failed detection at the aliquot level. Finally, the DeWorm3 Assay failed to detect *A. duodenale* in at least 1 of 12 technical replicates for 1 standard (#10.2) (**Appendix A4, Table A4.3**), which did not fail detection at the aliquot level.

1) Comparison of qPCR Assay: DeWorm3 vs. Smith College

The failed detection of *T. trichiura* by the DeWorm3 Assay was investigated first by comparison of qPCR Assays. Quantigen-extracted DNA from all three aliquots of Standards #4.2, #13, and #16 were shipped to Smith College for testing by the Smith College qPCR Assay. The Smith College Assay produced nearly identical results to the DeWorm3 Assay at the aliquot and technical replicate level, suggesting either (a) sub-optimal DNA extraction by the DeWorm3 procedure or (b) aliquot-to-aliquot variability in the spiked-stool standards created by Smith College (**Appendix A4, Table A4.4**).

2) Comparison of DNA Extraction Procedures: DeWorm3 vs. Smith College

Due to the robust nature of *T. trichiura* eggs, sub-optimal lysis by the DeWorm3 DNA extraction procedure was suspected and investigated. Increased bead-beating (lysis) times and different bead mixtures (i.e., Omni International beads utilized in the DeWorm3 extraction vs. MP Bio beads utilized in the Smith College extraction) were compared across a sub-set of standard aliquots, but no clear differences were observed (**Appendix A4, Table A4.5**).

3) Aliquot-to-Aliquot Variability of Standards

Finally, aliquot-to-aliquot variability was investigated through multiple extractions of Standard #11 with the Smith College extraction procedure (utilizing both Omni International beads and MP Bio beads) and the Smith College qPCR assay. Smith College observed dropout of *T. trichiura* detection across aliquots extracted with both bead types, suggesting that inconsistent detection of *T. trichiura* by the DeWorm3 Assay was likely due to variability across aliquots of the standards (**Appendix A4, Table A4. 6**).

It is important to note that *T. trichiura* material utilized in the creation of spiked-stool standards was limited, as exhibited by the high Ct Scores (Mean Ct of 30) observed across Smith and Quantigen during initial characterization (**Appendix A4, Table A4. 7**). Further, since the exact STH egg-count spiked into each standard is unknown, it is impossible to determine whether eggs were poorly dispersed across aliquots, or if the concentration of *T. trichiura* material is skirting the limit of detection for both the Smith College and DeWorm3 Assays.

Following the above outlined experiments, Smith College created new *T. trichiura* standards by spiking-in more than one *T. trichiura*-positive infected stool sample (3-4 total samples) into helminth naïve stool. These new standards (NewTt_4, NewTt_5, NewTt_6, and NewTt_7) performed consistently across aliquots and technical replicates tested by both the Smith College Assay and the DeWorm3 Assay (**Appendix A4, Table A4. 8**).

Three additional standards (#10.2, #12, and #17) produced inconsistent results and were removed from the panel. Standard #12 and #17 demonstrated a large deviation in Ct score across Smith aliquots, in addition to technical replicate drop out at Quantigen (**Appendix A4, Table A4. 9** & **Table A4. 2**). Standard #10.2 demonstrated technical replicate dropout for *A. duodenale* at Quantigen (**Appendix A4, Table A4. 3**).

Species	Count
Tt	5
Al	11
Na	10
Ad	1
**Total**	**18**

Standard #	STH Status			
1		Al	Na	
2			Na	
7		Al		
12v2			Na	
14		Al		
15		Al	Na	
18		Al		
19	Tt	Al	Na	
21				
22				
23				
24				
25				
27		Al	Na	Ad
NewTt_4	Tt	(Al)	Na	
NewTt_5	Tt	Al	Na	
NewTt_6	Tt	Al	Na	
NewTt_7	Tt	Al	(Na)	

Figure 4: DeWorm3 Spiked Stool Standards. STH-status represented in parentheses denotes STH which were shown to be detected inconsistently at both Smith and Quantigen.

Characterization of Final Panel

The final panel (**Figure 4**) performed consistently (at the technical replicate and aliquot level) at Smith College and Quantigen with all standards meeting the target of 100% Inter-laboratory concordance. Raw data is presented in **Appendix A4** (**Table A4. 8, Table A4. 10, Table A4. 11, Table A4. 12**).

Of note, standards NewTt_4 and NewTt_7 are suspected to include low quantities of *A. lumbricoides* and *N. americanus*, respectively, due to sporadic detection of these species across technical replicates at both Smith and Quantigen (**Figure 5**). These "weak positives" are likely due to a low-intensity *A. lumbricoides* or *N. americanus* infection in one of the 3-4 infected stool samples utilized in the preparation of these standards (**Figure 3**). Thus, for the purposes of validation and use of the Standards Panel for proficiency testing either positive or negative amplification may be acceptable for these species in these standards; this is denoted by parentheses in **Figure 4**.

		Positive Amplification (Tech. Replicates)			
		Tt	(Al)	Na	Ad
**NewTt_4**	**Smith**	12	2	12	0
	**Quantigen**	12	2	12	0

		Positive Amplification (Tech. Replicates)			
		Tt	Al	(Na)	Ad
**NewTt_7**	**Smith**	12	12	1	0
	**Quantigen**	12	12	5	0

Figure 5: Technical Replicate Positive STH status Smith vs. Quantigen for NewTt_4 and NewTt_7.

Conclusion

Ultimately, a final panel of 18 consistent Spiked-Stool Standards (13 STH-positive and 5 STH-negative, **Figure 4**) was utilized for DeWorm3 Assay Validation. This final panel will be validated at CMC for site qualification.[c] The final data from all three sites will be compared to ensure 100% interlaboratory concordance, but the current results point to strong interlaboratory concordance.

[c] CMC has yet to complete testing due to delays in receipt of supplies. Results will be reported when testing is completed at this site.

I.B.ii. Evaluation of Assay Accuracy

Methods Summary

Assay accuracy was evaluated on the technical replicate level and the aliquot level using the final panel of 18 standards. Three blinded aliquots (~85 mg) of each of the 18 standards were extracted and qPCR tested in quadruplicate by the DeWorm3 Assay (18 standards x 3 aliquots x 4 technical replicates). Accuracy was calculated using **Equation 1**. The true positive or negative status of each standard was defined by Smith College's initial characterization (**Figure 4**). Evaluation of STH positivity at the aliquot level was defined as at least one positive amplification out of all technical replicates tested by the DeWorm3 Assay. Well failures were not included in calculations of assay accuracy.

$$(1) Accuracy = \frac{True\ Positives + True\ Negatives}{True\ Positives + True\ Negatives + False\ Positives + False\ Negatives}$$

Table 4: DeWorm3 Assay Accuracy: Individual Extraction and Technical replicate level.

Assay Accuracy				
**Level**	*T. trichiura*	*A. lumbricoides*	*N. americanus*	*A. duodenale*
Technical Replicate	99.5%	100.0%	100.0%	100.0%
Individual Extraction	98.1%	100.0%	100.0%	100.0%

Assay Accuracy on the technical replicate level met the minimum target with > 99% accuracy for all assays (**Table 4**). *A. lumbricoides, N. americanus,* and *A. duodenale* assays met the minimum 100% accuracy at the individual extraction level. One *T. trichiura* false positive present in standard #27, however, decreased the individual extraction level accuracy for *T. trichiura* to 98.1% (**Table 4**). See Appendix A4 for raw data.

The above-mentioned false positive was due to erroneous *T. trichiura* amplification in 1 out of 12 technical replicates of standard #27 (36.8 Ct, **Table A4. 8**). Upon quadruplicate repeat testing at Quantigen in DeWorm3 multiplex and singleplex assays (n=8), this *T. trichiura* amplification was not reproducible.

All the species present in the stool standards were amplified in all three aliquots. All the standard aliquots amplified helminth targets ≥ 3 out of the 4 qPCR wells and < 1% of all qPCR wells failed to amplify (**Table A4. 13**). None of the STH negative stool standards (n=5) amplified erroneous targets.

All replicate wells in which at least one of the expected STH targets did not amplify, *B. atrophaeus* (internal extraction control) also failed to amplify. No false negatives were observed.

[c] CMC has yet to complete testing due to delays in receipt of supplies. Results will be reported when testing is completed at this site.

I.B.iii. Evaluation of Assay Specificity

Methods Summary

Assay specificity was assessed using 200 confirmed STH-negative stool samples per DeWorm3 site (n=413)[d]. Quantigen identified 213 and 200 STH-negative samples from the Benin and India sites, respectively, using the DeWorm3 Assay. All 413 stool samples found STH-negative by the Deworm3 Assay were subsequently tested utilizing the Smith College assay. The panel of Benin negatives, the "Benin Specificity Panel" (n=213) were tested at Smith College[e] and the panel of India negatives, the "India Specificity Panel" (n=200) were tested at CMC; both Smith College and CMC utilized the Smith College extraction and qPCR procedure.

Results

Benin Specificity Panel

Smith College identified 3 samples from the Benin Specificity Panel to be positive for *N. americanus* (n=2) and *A. lumbricoides* (n=1) (**Appendix A5, Table A5. 1**). As outlined in the validation plan, these inconsistencies were investigated. First, DNA extracted by Smith College and Quantigen from all three discordant samples (DNA from initial testing at each site) were tested by the liquid and lyophilized DeWorm3 Assay at Quantigen. The STH status of the Smith College extracts were confirmed by the DeWorm3 Assay in liquid and lyophilized form (**Appendix A5, Table A5. 2**). However, one of the three Quantigen extracts were found positive for *A. lumbricoides* (n=1) by testing in both the liquid and lyophilized DeWorm3 Assay (**Appendix A5, Table A5. 3**).

To investigate aliquot-to-aliquot variability, three new aliquots of each discordant sample (3 samples x 3 aliquots) were re-extracted by the DeWorm3 procedure and tested by the DeWorm3 liquid Assay in single qPCR replicates. All previously detected STH were detected in at least 2 out of 3 aliquots extracted. (**Appendix A5, Table A5. 4**).

Discordance among different aliquots from the same sample, tested by the same procedure and assay, suggests the discordance between Smith and Quantigen results lies in the heterogenous distribution of STH eggs throughout stool sample aliquots.

India Specificity Panel

CMC identified 5 *N. americanus*-positive samples among those identified as negative in Quantigen's initial screening. Further investigation into these inconsistencies was conducted. Per the validation plan, these inconsistencies were interrogated. DNA extracted by CMC and Quantigen from the 5 discordant samples were tested in triplicate by the lyophilized DeWorm3 Assay at Quantigen (**Appendix Table A5. 5**, **Table A5. 6**). All original STH status of the extracts were confirmed.

---

[d] DeWorm3 samples collected in the Malawi site are not included in this report due to delays in sample shipment. Once received, assay specificity will be validated by the same methods.
[e] The 13 additional Benin samples were shipped to Smith because some samples were low volume. However, all 213 samples had sufficient volume upon extraction at Smith, thus, all results are reported here.

To investigate aliquot-to-aliquot variability, the 5 discordant samples were re-extracted. Three of 5 samples were re-extracted in duplicate, 2 of 5 samples were re-extracted once.[f] These 8 extracts (3 samples x 2 extractions + 2 samples x 1 extraction) were tested via the lyophilized DeWorm3 Assay in duplicate. The samples that were re-extracted once detected STH in all qPCR replicates. One sample extracted in duplicate detected STH across both aliquots. Two samples that extracted in duplicate were discordant in STH detections between extractions (**Appendix A5,Table A5. 7).**

Following the investigation into India Specificity discordance, the data suggests that the source of discordance between CMC and Quantigen is the uneven distribution of STH eggs throughout stool sample aliquots.

Specificity Go/No-Go

With Benin and India Specificity Panel results, the DeWorm3 Assay Specificity is 98%; this does not meet the minimum target of 99%. As demonstrated by the investigation of the discordant samples from the Benin and India Specificity Panels, however, it is likely this variability is due to the heterogeneity of stool samples.

I.B.iv. Evaluation of Assay Sensitivity/LOD

Methods Summary

Spiked stool standards were selected for the determination of the limit of detection (LOD) due to the availability of Kato-Katz data from the field samples used in their creation[g].

Approximately 500mg of each field sample was spiked into naïve feces for the development of all standards. **Equation 2** was used to calculate the concentration of STH (epg) in each standard. Estimated STH concentrations (epg) for each standard are listed in **Table 5**.

Table 5: Concentration of STH eggs in field samples used to make the validation standards.

Species	Standard	Field Sample Concentration
N. americanus	1	960 epg
A. lumbricoides	1	2100 epg
A. duodenale	27	500 epg*
T. trichiura	19	4224 epg

*Standard 27 was spiked with 13 ng of *A. duodenale* WGA material which equates to 500 eggs.

$$(2)\left(\frac{Eggs\ in\ Field\ Sample}{1g}\right) \times \left(\frac{Spiked\ Field\ Sample\ Mass}{Total\ Standard\ Mass}\right) = STH\ eggs\ per\ grams\ of\ Standard$$

[f] The two samples that were extracted once were done so due to exhaustion.

[g] Note that no *A. duodenale* egg material was available for standard development, thus Standard #27 was spiked with 13ng of *A. duodenale* WGA DNA, which equates to approximately 500 eggs.

Each standard was extracted by the DeWorm3 procedure. Extracted DNA for each standard was serially diluted 1:10 for a total of 6 dilution points. Each dilution point was tested by the DeWorm3 Assay in ten technical replicates (4 standards x 6 dilution points x 10 replicates) and the lowest concentration in which all ten replicates generated a positive signal was identified. Starting at a concentration 2-fold higher than this concentration, five 1:2 serial dilutions were created. Twenty technical replicates of each dilution point were tested by qPCR (4 standards x 5 dilution points x 20 replicates). The LOD was determined as the lowest concentration at which at least 19/20 technical replicates amplified for the target STH species (**Table A6. 1**).

Table 6: Concentration of STH+ in stool standards.

Species	Field Sample Mass	Naive Stool Mass	Conc. per gram**
N. americanus	~500 mg	6.052 g	73.26 epg
A. lumbricoides	~500 mg	6.052 g	160.26 epg
A. duodenale	500 eggs*	6.357 g	78.65 epg
T. trichiura	~500 mg	6.045 g	322.69 epg

*13 ng/500 eggs were spiked into naive stool due to lack of egg material

**Final concentration of STH eggs or DNA per gram of stool

The assay LOD was calculated using **Equation 3.** However, several assumptions are made in these calculations. First, it is assumed that the STH eggs are evenly distributed throughout the feces. Second, it is assumed that 500 mg of spiked stool is added to naïve stool, as the initial spiked amount was not recorded. Third, between 50 and 75 mg of feces is extracted and used to test for STH. To measure the LOD, it was assumed that the lowest allowable mass was extracted (50 mg).

$$\frac{STH\ eggs}{1\ g\ of\ Standard} \times 50\ mg\ aliquot \times qPCR\ dilution\ limit = LOD$$

Results

Quantigen determined the lowest concentration at which at least 19/20 technical replicates amplified for the target STH species (See **Table A6. 2** & **Table A6. 3** for *N. americanus* data, **Table A6. 4** & **Table A6. 5** for *A. lumbricoides* data, **Table A6. 6** & **Table A6. 7** for *A. duodenale* data, and **Table A6. 8** & **Table A6. 9** for *T. trichiura* data). Using **Equation 3**, Quantigen determined that the LOD for all standards was <1 epg for each species (**Table 6**), meeting the minimum target (**Table 7**).

Table 7: Limit of detection for each STH.

Species	Dilution Limit	LOD
N. americanus	2.50×10^{-5}	9.16×10^{-5}
A. lumbricoides	1.25×10^{-4}	1.00×10^{-3}
A. duodenale	1.25×10^{-2}	4.92×10^{-2}
T. trichiura	1.25×10^{-4}	2.02×10^{-3}

I.B.v. Evaluation of Assay Precision

Methods Summary

Three independent operators will extract DNA from all standards and test on qPCR using 2 separate QuantStudio qPCR machines in duplicate.

Table 8: Assay precision for technical and aliquot extractions.

Species	Max Ct TR*	Max Ct AQ**
N. americanus	1.18%	0.62%
A. lumbricoides	24.37%	8.99%
A. duodenale	1.90%	0.95%
T. trichiura	6.89%	3.09%

*Technical replicate
**Aliquot

Validation of protocol

The precision of the assay failed at the level of technical replicates but passed at the level of extraction (aliquots) (**Table 8**). One technical replicate out of 4 from standard 18 had an *A. lumbricoides* Ct score that was uncharacteristically higher than that of the other technical replicates for this aliquot. We speculate that this could be a loading error. It is important to note that the "clinical call" for this replicate would still be accurate.

I.B.vi. B Assay Validation Target Performance Specifications Go/No-Go Chart

II.B. Assay Validation

Section	Validation	Level	Variance: Minimum	Variance: Optimistic	Results: Observed	Results: Pass/Fail
I.B.i	Multi-Site Characterization	-	100%	100%	TBD	-
I.B.ii.	Accuracy	Technical Replicate	≥ 95%	100%	≥ 99.5%	Pass
		Individual Extraction	100%	100%	≥ 98.1%	Fail
I.B.iii.	Specificity	-	≥ 99%	100%	98%	Fail
I.B.iv.	Sensitivity/LOD	-	≤ 1 epg	≤ 1 epg	≤ 1 epg	Pass
I.B.v .	Precision	Technical Replicate	CV ≤ 10%	CV ≤ 5%	≤ 24.4%*	Fail
		Individual Extraction	CV ≤ 15%	CV ≤ 10%	≤ 9%	Pass

*Precision for all but one technical replicate of Standard #18 produced < 9.3% CV.

Table 9: Assay Validation Go/No-Go Table.

Additional Investigation

200 Sample Experiment

Methods Summary

The goal of the 200 Sample Experiment was to determine whether discordance seen during Assay Validation was due to sample heterogeneity or suboptimal assay performance. This experiment was done by taking a random 10% of the first 2,000 extracted samples and extracting four (4) aliquots of each sample. Each extraction was then run on both the DeWorm3 Assay and the comparator Smith College Assay. This generated a total of 2 data points per extraction, and a total of 8 data points for each sample. To test for site extraction differences, two extractions per sample were completed via the DeWorm3 protocol and two extractions per sample were completed via the Smith College protocol. A diagram illustrating the workflow for one sample is below in **Figure 6**.

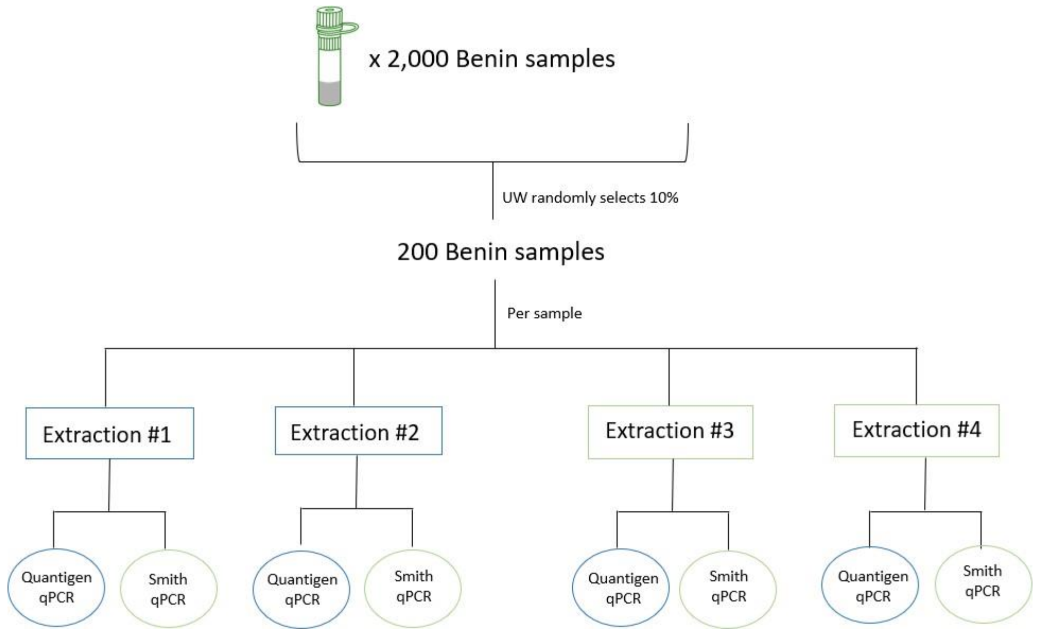

Figure 6: Sample workflow for the 200-sample experiment. This diagram shows how one sample generates eight points of data

Results

Between all four extractions, discordance was seen for all but one target, *A. duodenale*, which was negative for all extractions. 8/200 *N. americanus*, 3/200 *T. trichiura*, and 10/200 *A. lumbricoides* were found to be discordant.

When looking at the data on a site-by-site extraction basis, Quantigen saw discordance for *N. americanus* (6/200) and *A. lumbricoides* (8/200), while Smith College saw discordance for *N. americanus* (2/200), *T. trichiura* (1/200), and *A. lumbricoides* (3/200). Further looking at site-by-site performance, Quantigen and Smith found similar numbers of overall positivity (200 samples x 2 extractions = 400).

Extraction 1, 3, and 4 found 36 positives on a population level, while extraction 2 found 43. The remaining difference between extraction 2 and the other extractions can be reduced to unavoidable stochastic variability. These data are illustrated in **Figure 7** and show that despite the procedural differences, the DeWorm3 Assay and Smith College Assay have equivalent performance.

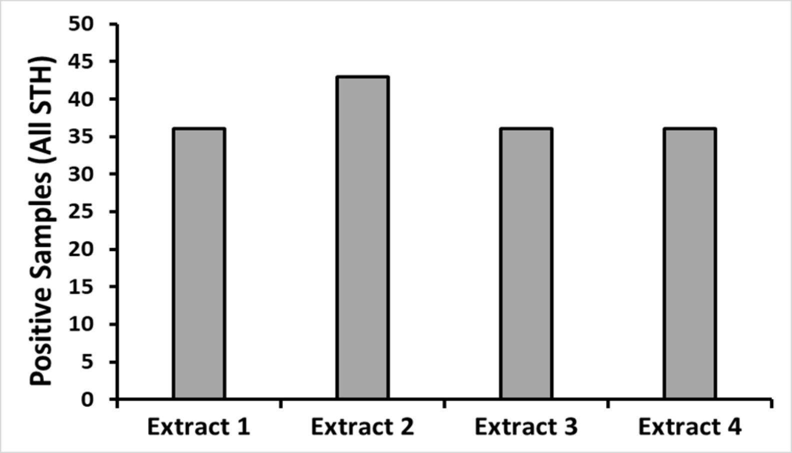

Figure 7: Positive Samples on a Population per extraction. Each extraction had an (n) of 200. Extract 1, Extract 3, and Extract 4 found (n)=36 positives and Extract 2 found (n)=43 positives

Further comparisons were made between the Smith College qPCR Assay and the Quantigen DeWorm3 Assay. This was done by comparing the results of one extract on the two assays. The two assays produced high amount of concordance between the labs, indicating that both assays are equivalent. Overall concordance for each species is as follows: *N. americanus* 98.5% concordance, *T. trichiura* 100% concordance, *A. duodenale* 100% concordance, and *A. lumbricoides* 98.1% concordance.

All line data for this experiment can be found in **Appendix A7 in S1 Appendix**

Conclusion

Looking at all these data, there is no clear assay performance differences between the DeWorm3 Assay and the Smith College Assay, leading to the conclusion that the Quantigen DeWorm3 Assay is performing at the necessary level for final validation.

This experiment also shows that the previous specificity inaccuracies were due to stool sample heterogeneity that cannot be avoided, regardless of the procedure used. The data confirms that the sample heterogeneity that is observed is not a function of the DeWorm3 assay itself. Additionally, the observed specificity from the Benin and India Specificity Panels shows that these measured specificity values are to be expected due to the nature of the samples.

This experiment will also be functionally repeated during the QC/QA testing built into the study. 10% of samples throughout the study, including those with lower intensity infections, will be retested between sites, allowing for further quantification of assay performance in samples with wide ranges of intensities.

Conclusion

Although there are some items of the Validation Plan that are considered to miss the target set out by the initial plan, upon completion of the 200-sample experiment, collaborators at Quantigen, Smith College, and the University of Washington are confident that the DeWorm3 Assay meets the criteria for final validation. All of the observed results indicate that inaccuracies seen are due to sample heterogeneity and are not a function of the DeWorm3 Assay.

Next Steps

Qualification of international labs are still pending completion due to shipping delays and COVID-19 related issues. This includes testing of standards at CMC for multi-site characterization (I.B.i) and evaluation of specificity using samples from the DeWorm3 Malawi site (I.B.iii). Results from these portions of validation will be reported to all principles upon completion, however, these data are not necessary for verifying that the Quantigen DeWorm3 Assay is validated.

Appendix A1: Raw Data: Validation of Run control- *Ascaris suum* spiked stool (I.A.i)
-

Table A1. 1: *A. suum Ct Scores for 25 aliquots of the DeWorm3 Ascaris suum-spiked stool (positive extraction control) extracted and qPCR tested by the DeWorm3 Assay*

Sample Name	CT - *Ascaris suum*
A1	19.26
A1	19.41
A2	19.71
A2	19.82
A3	19.81
A3	19.95
A4	20.75
A4	20.92
A5	21.20
A5	21.30
A6	20.90
A6	21.13
A7	20.27
A7	20.19
A8	21.22
A8	21.21
A9	21.47
A9	21.63
A10	20.70
A10	20.75
A11	20.85
A11	20.74
A12	21.14
A12	20.99
A13	20.49
A13	20.45
A14	21.35
A14	21.49
A15	21.42
A15	21.33
A16	20.95
A16	20.98
A17	22.20
A17	22.32
A18	21.09
A18	21.12
A19	21.44
A19	21.25
A20	21.99
A20	21.00
A21	22.47
A21	21.90
A22	23.06
A22	23.31
A23	22.02
A23	21.83
A24	22.07
A24	22.48
A25	20.93
A25	20.81

Appendix A2: Raw Data: Validation of Run Control- B. atrophaeus Validation (I.A.ii)

Table A2. 1: *B. atrophaeus Ct Scores from 413 STH-Negative Benin and India DeWorm3 Samples. Each sample has two Ct Scores for B. atrophaeus corresponding to each of the two wells/assays constituting the DeWorm3 Assay.*

Bacillus Ct Assay 1 (Na/Tt/Ba)	Bacillus Ct Assay 2(Ad/Al/Ba)
28.59	28.46
27.52	27.61
30.13	29.14
26.91	27.15
30.21	29.84
28.60	28.38
27.99	27.81
26.74	26.84
27.85	28.01
27.92	28.13
28.68	28.56
27.87	27.63
28.16	28.55
28.22	27.67
28.40	28.17
28.69	28.67
28.62	28.39
28.13	27.96
28.14	27.80
28.02	27.97
27.80	28.04
27.61	27.60
28.29	28.28
28.00	27.30
27.92	27.66
28.56	28.31
28.16	28.18
27.20	28.07
28.38	28.31
27.25	27.28
27.72	27.68
28.28	28.40
28.55	28.27
27.93	28.34
28.18	28.38
27.74	27.81
28.25	27.78
28.26	28.26
27.99	27.47
28.17	28.80
27.36	27.48
27.83	27.68
28.55	28.41
28.77	28.73
27.36	27.50
28.70	28.45
30.30	30.67
28.23	28.67
27.57	28.20

Appendices

Bacillus Ct Assay 1 (Na/Tt/Ba)	Bacillus Ct Assay 2(Ad/Al/Ba)
28.06	27.42
27.59	27.93
28.46	27.96
28.10	27.95
28.64	28.70
27.77	27.48
33.77	30.68
27.85	27.78
29.72	29.66
30.39	27.98
27.83	27.76
28.11	28.43
26.95	27.20
27.50	27.67
27.85	28.50
32.15	26.69
27.86	28.04
30.41	31.21
29.18	29.88
31.18	31.19
31.29	30.73
31.23	31.19
31.74	32.54
30.86	31.14
30.46	30.49
33.85	33.80
30.52	30.44
30.93	30.50
32.62	36.24
27.97	28.67
33.94	33.31
30.25	31.12
31.16	31.01
29.39	31.50
30.31	30.59
30.94	30.66
29.83	29.90
30.25	30.52
29.55	30.47
31.39	31.38
32.33	32.11
31.50	31.94
32.98	33.25
30.64	30.47
31.49	31.11
30.49	30.13
31.62	31.57
31.05	30.61
32.56	32.38
32.44	30.39

Appendices

Bacillus Ct Assay 1 (Na/Tt/Ba)	Bacillus Ct Assay 2(Ad/Al/Ba)
29.27	29.78
30.89	31.12
29.62	29.49
31.43	31.95
31.31	38.69
31.12	31.88
29.42	30.91
30.23	30.38
30.56	31.25
31.12	31.90
30.01	30.29
33.63	32.72
31.23	31.47
29.41	30.74
33.04	32.11
29.31	32.57
29.63	29.73
29.23	28.88
28.67	28.92
29.48	29.23
28.74	29.53
29.00	29.16
29.49	29.27
29.77	29.56
30.67	30.15
29.16	29.33
30.89	30.76
30.82	31.25
30.44	30.16
30.70	30.11
28.95	29.13
29.74	29.53
29.48	29.96
28.61	29.29
27.71	27.86
30.19	30.56
30.68	29.80
28.55	28.73
29.13	29.11
28.76	28.40
29.15	28.92
29.61	29.18
28.94	28.70
29.27	29.23
28.73	28.86
28.68	28.49
29.51	30.28
28.89	29.42
29.53	29.41
29.65	29.51

Appendices

Bacillus Ct Assay 1 (Na/Tt/Ba)	Bacillus Ct Assay 2(Ad/Al/Ba)
30.06	30.04
29.70	29.40
31.77	31.79
30.17	29.44
27.53	27.60
31.45	30.69
29.26	29.13
29.28	29.96
29.38	30.04
31.01	31.29
31.17	30.43
31.48	31.03
39.97	32.04
30.93	31.34
30.63	30.75
36.36	31.19
30.50	30.28
32.46	30.51
30.71	31.07
32.20	33.09
31.21	31.49
31.20	31.09
31.38	31.54
31.53	31.28
34.47	31.51
30.19	30.47
30.12	31.08
30.70	30.84
30.97	32.21
30.98	30.69
31.92	32.17
31.71	31.85
35.36	29.32
30.38	30.38
30.88	30.99
31.07	30.93
33.66	31.69
32.15	32.23
30.62	30.93
27.54	27.65
27.62	27.70
28.00	27.83
27.13	27.15
27.10	26.91
28.23	28.53
30.26	31.09
30.04	30.44
33.21	32.27
30.34	30.24
31.55	30.99

Appendices

Bacillus Ct Assay 1 (Na/Tt/Ba)	Bacillus Ct Assay 2(Ad/Al/Ba)
29.25	28.74
29.35	29.36
28.38	28.50
27.68	27.33
27.71	27.51
28.77	28.56
27.32	27.51
27.60	27.64
28.35	28.64
28.95	28.66
28.29	28.31
27.97	27.93
29.80	29.70
28.12	28.26
27.40	27.61
29.13	29.50
28.17	28.19
28.19	28.21
28.16	28.14
28.75	28.85
28.23	27.97
28.35	28.57
27.88	27.82
28.46	28.40
27.98	28.01
27.51	27.85
28.23	28.29
27.99	28.59
27.38	27.98
26.92	27.36
27.43	27.71
28.01	27.79
28.66	29.79
27.94	27.94
27.56	27.56
27.69	27.71
27.52	28.21
26.83	26.74
27.05	27.21
28.11	28.02
27.44	27.62
28.38	28.59
29.73	29.73
27.73	27.98
28.70	28.52
28.83	28.63
28.24	27.98
27.82	27.56
28.39	28.15
29.08	29.20

Bacillus Ct Assay 1 (Na/Tt/Ba)	Bacillus Ct Assay 2(Ad/Al/Ba)
28.32	28.34
28.27	28.32
28.65	28.65
28.96	28.94
28.11	28.22
28.74	28.45
28.15	28.21
27.39	27.62
30.61	31.73
31.55	32.07
31.84	31.89
31.56	31.66
30.43	31.26
31.09	32.21
30.35	31.18
31.86	32.47
30.66	31.55
31.16	32.64
31.22	30.91
30.74	31.29
32.14	32.99
31.77	32.00
30.62	31.36
31.70	31.83
31.08	31.35
30.69	31.42
31.14	31.23
32.41	33.65
31.52	31.33
31.26	32.07
31.77	31.86
31.98	31.34
31.46	31.55
32.17	31.89
31.44	31.54
30.72	31.68
33.78	32.44
31.94	32.77
32.13	33.35
31.44	32.44
31.34	32.20
30.97	31.22
31.52	31.62
31.51	32.04
31.80	33.01
30.85	30.96
31.83	32.09
31.71	31.82
32.80	31.84
31.50	32.78

Bacillus Ct Assay 1 (Na/Tt/Ba)	Bacillus Ct Assay 2(Ad/Al/Ba)
31.21	31.48
33.06	36.11
31.49	31.83
32.19	32.35
30.90	32.34
30.97	31.84
31.52	31.08
31.30	31.26
30.79	31.51
31.69	32.35
32.73	34.06
35.21	35.32
34.20	34.21
33.28	32.41
33.07	34.90
33.55	32.73
33.33	33.20
28.39	28.50
27.11	26.84
27.62	27.55
27.75	28.19
26.09	26.63
26.93	26.85
26.29	26.22
27.34	27.58
27.39	27.46
28.15	28.21
27.94	27.89
28.22	28.01
27.10	27.37
27.33	27.35
26.56	26.70
28.00	28.01
27.40	27.31
26.34	26.72
26.91	26.99
27.07	26.98
26.20	26.33
28.20	28.01
27.17	27.39
27.58	27.58
27.85	27.85
26.92	26.93
28.01	27.93
27.46	27.81
27.10	26.98
27.45	27.48
27.19	27.27
26.46	26.67
37.98	31.84

Bacillus Ct Assay 1 (Na/Tt/Ba)	Bacillus Ct Assay 2(Ad/Al/Ba)
31.40	31.01
31.78	31.11
32.63	32.99
31.62	32.24
31.44	31.67
31.06	31.35
30.59	30.92
32.96	32.56
30.90	31.28
32.79	32.54
31.98	31.85
31.62	32.65
31.84	31.72
30.90	31.82
32.26	31.69
31.34	31.05
31.57	31.41
30.88	31.62
30.97	32.05
30.98	30.97
31.19	31.24
31.71	32.97
31.79	31.62
31.52	31.80
30.77	31.42
29.15	30.39
30.56	31.36
32.89	31.42
30.17	29.91
31.28	31.07
30.28	30.15
31.12	30.74
30.38	30.72
31.67	31.45
32.60	35.30
31.49	31.54
32.14	32.01
32.58	32.90
32.47	32.38
33.42	32.75
31.50	30.76
31.51	31.54
32.14	31.66
31.77	30.89
31.84	31.72
33.45	33.61
32.08	30.97
31.52	30.43
32.51	32.28
31.56	31.44
31.23	31.46
30.91	31.90
30.63	30.78
32.23	31.78
32.65	32.46
30.99	34.77
31.61	31.11
31.21	31.44
31.89	31.44
31.77	31.91
30.97	30.57
31.16	31.23
32.08	31.92

Appendix A3: Raw Data: Validation of Run Control-WGA STH nucleic acid (I.A.iii)

Table A3. 1: *Validation of 25 aliquots WGA STH nucleic acid (positive qPCR control). Ct Scores for each STH species are displayed.*

WGA Run Controls				
**Sample**	***A. duodenale***	***A. lumbricoides***	***N. americanus***	***T. trichiura***
gDNA 1	28.57	32.30	30.89	29.02
gDNA 2	28.90	32.56	28.73	27.21
gDNA 3	29.57	33.47	29.80	28.08
gDNA 4	29.57	33.15	29.57	27.97
gDNA 5	28.86	29.13	28.49	28.17
gDNA 6	29.19	32.83	28.78	27.65
gDNA 7	29.15	32.87	27.91	27.76
gDNA 8	28.62	30.48	31.27	29.20
gDNA 9	29.11	29.93	29.93	27.81
gDNA 10	29.19	31.21	30.10	28.37
gDNA 11	28.26	31.47	31.19	27.57
gDNA 12	28.99	31.01	28.99	27.72
gDNA 13	37.47	37.84	29.10	26.94
gDNA 14	28.80	34.49	30.61	27.69
gDNA 15	31.97	33.28	29.65	27.54
gDNA 16	28.47	32.38	28.88	28.12
gDNA 17	29.01	31.68	29.63	27.37
gDNA 18	29.14	28.97	29.05	27.76
gDNA 19	28.93	31.19	30.34	27.46
gDNA 20	28.56	30.56	29.56	27.99
gDNA 21	28.49	32.66	29.43	28.12
gDNA 22	28.52	33.07	27.74	27.89
gDNA 23	29.77	32.45	30.33	27.34
gDNA 24	28.55	29.27	30.51	27.82
gDNA 25	29.65	32.55	31.24	27.66

Appendix A4: Raw Data: Multi-site Characterization of Spiked Stool Standards (I.B.i)

Table A4. 1: *Initial Validation Panel Candidates Quantigen data (Technical Replicates), **T. trichiura Assay**.*

T. trichiura												
	Ct Scores											
Smith Panel ID	Extraction #1				Extraction #2				Extraction #3			
1	Undetermined	Undetermined	Undetermined	Undetermined	Undetermined	Undetermined	Undetermined	Undetermined	Undetermined	Undetermined	Undetermined	Undetermined
2	Undetermined	Undetermined	Undetermined	Undetermined	Undetermined	Undetermined	Undetermined	Undetermined	Undetermined	Undetermined	Undetermined	Undetermined
3	40.00	32.05	37.80	34.52	35.68	36.93	Well Failure	36.55	32.59	33.72	35.41	30.97
7	Well Failure	Undetermined	Undetermined	Undetermined	Undetermined	Undetermined	Undetermined	Undetermined	Undetermined	Undetermined	Undetermined	Undetermined
8	38.53	34.36	33.57	34.07	35.48	36.28	34.31	35.47	35.14	40.00	35.97	36.69
9	40.00	32.31	33.46	33.07	33.56	40.00	33.11	28.00	35.03	32.92	33.59	35.00
11	35.36	36.18	40.00	33.91	35.52	33.79	33.34	33.76	40.00	34.48	35.43	32.94
12	Undetermined	Undetermined	Undetermined	Undetermined	Undetermined	Undetermined	Undetermined	Undetermined	Undetermined	Undetermined	Undetermined	Undetermined
13	40.00	40.00	40.00	40.00	40.00	40.00	40.00	40.00	35.93	34.44	36.22	40.00
14	Undetermined	Undetermined	Undetermined	Undetermined	Undetermined	Undetermined	Undetermined	Well Failure	Undetermined	Undetermined	Undetermined	Undetermined
15	Undetermined	Undetermined	Undetermined	Undetermined	Undetermined	Undetermined	Undetermined	Undetermined	Well Failure	Undetermined	Undetermined	Undetermined
16	30.84	30.63	30.76	31.60	40.00	40.00	40.00	40.00	35.04	33.99	35.29	35.44
17	Undetermined	Undetermined	Undetermined	Undetermined	Undetermined	Undetermined	Undetermined	Undetermined	Undetermined	Undetermined	Undetermined	Undetermined
18	Undetermined	Undetermined	Undetermined	Undetermined	Well Failure	Undetermined	Undetermined	Undetermined	Undetermined	Undetermined	Undetermined	Undetermined
19	34.08	32.61	32.99	33.54	Well Failure	33.10	32.54	32.73	32.81	35.78	39.73	35.38
20	35.30	34.83	35.05	35.65	38.35	35.67	34.97	35.71	40.00	40.00	35.35	36.00
4.2	36.46	35.53	36.57	35.70	40.00	40.00	40.00	40.00	40.00	36.52	40.00	40.00
10.2	Undetermined	Undetermined	Undetermined	Undetermined	Undetermined	Undetermined	Undetermined	Undetermined	Undetermined	Undetermined	Undetermined	Undetermined
27	Undetermined	Undetermined	Undetermined	Undetermined	Undetermined	Undetermined	36.76	Undetermined	Undetermined	Undetermined	Undetermined	Undetermined
12.2	Undetermined	Undetermined	Undetermined	Undetermined	Undetermined	Undetermined	Undetermined	Undetermined	Undetermined	Undetermined	Undetermined	Undetermined
21	Undetermined	Undetermined	Undetermined	Undetermined	Undetermined	Undetermined	Undetermined	Undetermined	Undetermined	Undetermined	Undetermined	Undetermined
22	Undetermined	Undetermined	Undetermined	Undetermined	Undetermined	Undetermined	Undetermined	Undetermined	Undetermined	Undetermined	Undetermined	Undetermined
23	Undetermined	Undetermined	Undetermined	Undetermined	Undetermined	Undetermined	Undetermined	Undetermined	Undetermined	Undetermined	Undetermined	Undetermined
24	Undetermined	Undetermined	Undetermined	Undetermined	Undetermined	Undetermined	Undetermined	Undetermined	Undetermined	Undetermined	Undetermined	Undetermined
25	Undetermined	Undetermined	Undetermined	Undetermined	Undetermined	Undetermined	Undetermined	Undetermined	Undetermined	Undetermined	Undetermined	Undetermined

Table A4. 2: *Initial Validation Panel Candidates Quantigen data (Technical Replicates), N. americanus Assay. Standard #17 was dropped from the final DeWorm3 Spiked Stool Standards Panel due to inconsistent Ct Scores across Smith and Quantigen Data; Smith data is summarized in Table A6.3.*

N. americanus												
	Ct Scores											
Smith Panel ID	Extraciton #1				Extraction #2				Extraction #3			
1	21.27	21.31	21.27	21.39	21.86	21.43	21.52	21.93	23.06	22.82	23.10	23.02
2	27.17	27.06	27.54	27.06	29.62	29.54	29.89	29.68	26.03	26.47	25.82	26.21
3	Undetermined	Undetermined	Undetermined	Undetermined	Undetermined	Undetermined	Well Failure	Undetermined	Undetermined	Undetermined	Undetermined	Undetermined
7	Well Failure	Undetermined	Undetermined	Undetermined	Undetermined	Undetermined	Undetermined	Undetermined	Undetermined	Undetermined	Undetermined	Undetermined
8	18.63	24.16	24.05	24.30	32.94	31.81	32.63	32.97	29.37	29.50	29.25	29.32
9	31.08	29.89	29.78	29.73	24.71	31.29	24.80	24.74	29.46	30.02	29.50	29.46
11	30.10	29.78	29.91	29.88	25.60	25.50	25.66	25.54	36.30	29.75	30.39	30.13
12	40.00	35.65	32.84	33.16	33.02	34.49	32.93	37.33	40.00	35.59	34.22	35.08
13	Undetermined	Undetermined	Undetermined	Undetermined	Undetermined	Undetermined	Undetermined	Undetermined	Undetermined	Undetermined	Undetermined	Undetermined
14	Undetermined	Undetermined	Undetermined	Undetermined	Undetermined	Undetermined	Undetermined	Well Failure	Undetermined	Undetermined	Undetermined	Undetermined
15	27.45	27.48	27.44	27.46	28.40	28.57	28.71	28.85	Well Failure	28.68	27.87	28.35
16	Undetermined	Undetermined	Undetermined	Undetermined	Undetermined	Undetermined	Undetermined	Undetermined	Undetermined	Undetermined	Undetermined	Undetermined
17	26.88	26.94	27.05	27.34	36.35	40.00	40.00	36.66	37.85	40.00	40.00	40.00
18	Undetermined	Undetermined	Undetermined	Undetermined	Well Failure	Undetermined	Undetermined	Undetermined	Undetermined	Undetermined	Undetermined	Undetermined
19	21.72	21.66	21.87	21.99	Well Failure	23.58	23.43	23.43	22.04	22.14	22.17	22.14
20	23.77	24.51	24.35	24.53	26.27	26.56	26.44	26.69	25.95	25.98	26.25	26.06
4.2	Undetermined	Undetermined	Undetermined	Undetermined	Undetermined	Undetermined	Undetermined	Undetermined	Undetermined	Undetermined	Undetermined	Undetermined
10.2	25.23	25.04	24.74	25.07	26.49	26.61	26.41	26.23	26.81	26.65	26.92	26.80
27	27.23	27.80	27.53	27.82	27.39	27.48	27.35	27.50	27.52	27.43	27.56	27.37
12.2	28.47	28.15	28.34	28.20	27.38	27.51	26.89	27.19	27.50	27.47	27.41	27.40
21	Undetermined	Undetermined	Undetermined	Undetermined	Undetermined	Undetermined	Undetermined	Undetermined	Undetermined	Undetermined	Undetermined	Undetermined
22	Undetermined	Undetermined	Undetermined	Undetermined	Undetermined	Undetermined	Undetermined	Undetermined	Undetermined	Undetermined	Undetermined	Undetermined
23	Undetermined	Undetermined	Undetermined	Undetermined	Undetermined	Undetermined	Undetermined	Undetermined	Undetermined	Undetermined	Undetermined	Undetermined
24	Undetermined	Undetermined	Undetermined	Undetermined	Undetermined	Undetermined	Undetermined	Undetermined	Undetermined	Undetermined	Undetermined	Undetermined
25	Undetermined	Undetermined	Undetermined	Undetermined	Undetermined	Undetermined	Undetermined	Undetermined	Undetermined	Undetermined	Undetermined	Undetermined

Table A4. 3*: *Initial Validation Panel Candidates Quantigen data (Technical Replicates),* *A. duodenale Assay*. *Standard #10.2 was dropped from the final DeWorm3 Spiked Stool Standards Panel due to the drop-out of technical replicates observed during DeWorm3 Validation; it is suspected that the gDNA spiked into naïve stool at low-concentration led to degradation of the DNA.

	A. duodenale											
	Ct Scores											
Smith Panel ID	Extraction #1				Extraction #2				Extraction #3			
1	Undetermined	Undetermined	Undetermined	Undetermined	Undetermined	Undetermined	Undetermined	Undetermined	Undetermined	Undetermined	Undetermined	Undetermined
2	Undetermined	Undetermined	Undetermined	Undetermined	Undetermined	Undetermined	Undetermined	Undetermined	Undetermined	Undetermined	Undetermined	Undetermined
3	Undetermined	Undetermined	Undetermined	Undetermined	Undetermined	Undetermined	Undetermined	Undetermined	Undetermined	Undetermined	Undetermined	Undetermined
7	Undetermined	Undetermined	Undetermined	Undetermined	Undetermined	Undetermined	Undetermined	Undetermined	Undetermined	Undetermined	Undetermined	Undetermined
8	Undetermined	Undetermined	Undetermined	Undetermined	Undetermined	Undetermined	Undetermined	Undetermined	Undetermined	Undetermined	Undetermined	Undetermined
9	Undetermined	Undetermined	Undetermined	Undetermined	Undetermined	Undetermined	Undetermined	Undetermined	Undetermined	Undetermined	Undetermined	Undetermined
11	Undetermined	Undetermined	Undetermined	Undetermined	Undetermined	Undetermined	Undetermined	Undetermined	Undetermined	Undetermined	Undetermined	Undetermined
12	Undetermined	Undetermined	Undetermined	Undetermined	Undetermined	Undetermined	Undetermined	Undetermined	Undetermined	Undetermined	Undetermined	Undetermined
13	Undetermined	Undetermined	Undetermined	Undetermined	Undetermined	Undetermined	Undetermined	Undetermined	Undetermined	Undetermined	Undetermined	Undetermined
14	Undetermined	Undetermined	Undetermined	Undetermined	Undetermined	Undetermined	Undetermined	Undetermined	Undetermined	Undetermined	Undetermined	Undetermined
15	Undetermined	Undetermined	Undetermined	Undetermined	Undetermined	Undetermined	Undetermined	Undetermined	Undetermined	Undetermined	Undetermined	Undetermined
16	Undetermined	Undetermined	Undetermined	Undetermined	Undetermined	Undetermined	Undetermined	Undetermined	Undetermined	Undetermined	Undetermined	Undetermined
17	Undetermined	Undetermined	Undetermined	Undetermined	Undetermined	Undetermined	Undetermined	Undetermined	Undetermined	Undetermined	Undetermined	Undetermined
18	Undetermined	Undetermined	Undetermined	Undetermined	Undetermined	Undetermined	Undetermined	Undetermined	Undetermined	Undetermined	Undetermined	Undetermined
19	Undetermined	Undetermined	Undetermined	Undetermined	Undetermined	Undetermined	Undetermined	Undetermined	Undetermined	Undetermined	Undetermined	Undetermined
20	Undetermined	Undetermined	Undetermined	Undetermined	Undetermined	Undetermined	Undetermined	Undetermined	Undetermined	Undetermined	Undetermined	Undetermined
4.2	Undetermined	Undetermined	Undetermined	Undetermined	Undetermined	Undetermined	Undetermined	Undetermined	Undetermined	Undetermined	Undetermined	Undetermined
10.2	34.10	34.28	34.47	34.06	38.79	40.00	36.67	40.00	40.00	40.00	39.88	36.71
27	30.59	30.00	30.41	30.40	30.16	30.28	30.19	30.34	31.34	32.76	31.28	31.55
12.2	Undetermined	Undetermined	Undetermined	Undetermined	Undetermined	Undetermined	Undetermined	Undetermined	Undetermined	Undetermined	Undetermined	Undetermined
21	Undetermined	Undetermined	Undetermined	Undetermined	Undetermined	Undetermined	Undetermined	Undetermined	Undetermined	Undetermined	Undetermined	Undetermined
22	Undetermined	Undetermined	Undetermined	Undetermined	Undetermined	Undetermined	Undetermined	Undetermined	Undetermined	Undetermined	Undetermined	Undetermined
23	Undetermined	Undetermined	Undetermined	Undetermined	Undetermined	Undetermined	Undetermined	Undetermined	Undetermined	Undetermined	Undetermined	Undetermined
24	Undetermined	Undetermined	Undetermined	Undetermined	Undetermined	Undetermined	Undetermined	Undetermined	Undetermined	Undetermined	Undetermined	Undetermined
25	Undetermined	Undetermined	Undetermined	Undetermined	Undetermined	Undetermined	Undetermined	Undetermined	Undetermined	Undetermined	Undetermined	Undetermined

Table A4. 4: *Investigation of inconsistent T. trichiura containing standards: Comparison of qPCR procedures. Each of the three Quantigen extracts from standards #13, #4.2, and #16 were tested by Smith College qPCR Assay in quadruplicate. Comparing the DeWorm3 qPCR Assay results "Quantigen" and the Smith College Results "Smith," suggests the discordance of these standard is not due to significant differences in detection by qPCR procedure.*

T. trichiura													
	**Ct Scores**												
**Smith Panel ID**		**Extraction #1**				**Extraction #2**				**Extraction #3**			
13	Quantigen	40.00	40.00	40.00	40.00	40.00	40.00	40.00	40.00	35.93	34.44	36.22	40.00
	Smith	40.00	40.00	40.00	40.00	40.00	40.00	40.00	40.00	33.26	33.26	40.00	40.00
4.2	Quantigen	36.46	35.53	36.57	35.70	40.00	40.00	40.00	40.00	40.00	36.52	40.00	40.00
	Smith	35.89	35.89	40.00	40.00	40.00	40.00	40.00	40.00	40.00	40.00	40.00	40.00
16	Quantigen	30.84	30.63	30.76	31.60	40.00	40.00	40.00	40.00	35.04	33.99	35.29	35.44
	Smith	32.75	33.59	31.12	33.18	40.00	40.00	40.00	40.00	37.06	35.71	34.20	35.19

Table A4. 5: *Investigation of inconsistent T. trichiura containing standards: Comparison of DNA Extraction, Bead beating procedures. Note all Ct scores represent a single aliquot and qPCR replicate (i.e., each bead type was tested with a unique aliquot of each Standard). Smith College Beadbeating mix is "MP Bio" and the DeWorm3 Assay Beadbeating mix "Omni".*

**ID**	**Standard Status**	***N. americanus***				***T. trichiura***				***A. lumbricoides***			
		**3 min**		**30 min**		**3 min**		**30 min**		**3 min**		**30 min**	
		**MP Bio**	**Omni**	**MP Bio**	**Omni**	**MP Bio**	**Omni**	**MP Bio**	**Omni**	**MP Bio**	**Omni**	**MP Bio**	**Omni**
**Std 8**	**Tt, Al, Na**	29.70	24.73	25.75	22.24	35.08	34.95	33.59	33.67	21.23	21.57	20.82	19.96
**Std 11**	**Tt, Na**	26.42	27.32	31.59	27.12	33.40	UD	33.82	31.91	35.61	UD	27.90	UD
**Std 13**	**Tt**	UD	UD	UD	UD	UD	33.04	UD	37.61	UD	UD	UD	38.86
**Std 16**	**Tt, Al**	UD	UD	UD	UD	32.48	31.70	29.87	31.55	14.88	14.77	14.23	14.00

Table A4. 6: *Investigation of inconsistent T. trichiura containing standards. Smith College extracted 6 additional aliquots of Standard #11 with the Smith College Beadbeating mix (MP Bio "Mp", n=3) and the DeWorm3 Assay Beadbeating mix (Omni "O", n=3). Samples were qPCR tested in quadruplicate and tested on two qPCR plates (Run 1 & Run 2), see Panel 1. The same experiment was repeated in Panel 2 with 6 additional aliquots of Standard #11.*

Panel 1: Initial Experiment

	qPCR Run 1	qPCR Run 2
**Sample Name**	**Tt Cт**	**Tt Cт**
Mp #1	31.08	37.75
Mp #1	UD	UD
Mp #1	29.90	35.97
Mp #1	33.69	39.78
Mp #2	UD	33.05
Mp #2	31.16	34.29
Mp #2	UD	30.80
Mp #2	UD	31.12
Mp #3	32.50	30.83
Mp #3	31.05	30.96
Mp #3	29.92	30.57
Mp #3	29.84	29.42
O #1	33.84	32.57
O #1	34.00	30.60
O #1	34.25	UD
O #1	31.94	30.85
O #2	30.63	30.44
O #2	30.21	29.59
O #2	30.34	30.22
O #2	34.09	29.63
O #3	34.58	UD
O #3	33.02	32.14
O #3	34.44	31.43
O #3	33.09	31.76

Panel 2: Repeat

Sample Name	Tt Cт
MP v2 #1	32.71
MP v2 #1	29.43
MP v2 #1	29.53
MP v2 #1	33.25
MP v2 #2	29.66
MP v2 #2	29.32
MP v2 #2	28.54
MP v2 #2	27.75
MP v2 #3	34.57
MP v2 #3	30.33
MP v2 #3	34.39
MP v2 #3	30.05
O v2 #1	32.03
O v2 #1	32.46
O v2 #1	34.01
O v2 #1	37.24
O v2 #2	29.81
O v2 #2	29.42
O v2 #2	29.95
O v2 #2	32.19
O v2 #3	UD
O v2 #3	UD
O v2 #3	35.20
O v2 #3	UD

Table A4. 7: Initial Validation Panel, Smith vs. Quantigen Aliquot-Level ***T. trichiura* Assay.** Note that Smith College Ct Scores provide the standard deviation in parentheses.

		T. trichiura Ct (Mean- 4 Replicates)					
**Smith Panel ID**	**Sample Summary Status**	**Smith College**			**Quantigen**		
		**Aliquot A**	**Aliquot B**	**Aliquot C**	**Aliquot Q1**	**Aliquot Q2**	**Aliquot Q3**
1	**Al, Na**				**neg**	**neg**	**neg**
2	**Na**				**neg**	**neg**	**neg**
3	**Tt**	**32.15 (1.84)**	**32.73 (2.36)**	**33.23 (2.17)**	**34.79**	**36.39**	**33.17**
7	**Al**				**neg**	**neg**	**neg**
8	**Tt, Al, Na**	**30.71 (0.81)**	**29.34(0.75)**	**30.41(1.19)**	**35.13**	**35.38**	**35.93**
9	**Tt, Al, Na**	**27.54 (0.59)**	**28.08 (0.58)**	**28.95 (0.69)**	**32.95**	**31.56**	**34.13**
11	**Tt, Na**	**29.35 (0.73)**	**30.28 (0.59)**	**30.47 (0.86)**	**35.15**	**34.10**	**34.28**
12	**Na**				**neg**	**neg**	**neg**
13	**Tt**	**30.24 (0.86)**	**30.29 (0.58)**	**29.92 (0.36)**	**neg**	**neg**	**35.53**
14	**Al**				**neg**	**neg**	**neg**
15	**Al, Na**				**neg**	**neg**	**neg**
16	**Tt, Al**	**29.50 (0.60)**	**29.40 (0.59)**	**32.88 (2.16)**	**30.96**	**neg**	**34.94**
17	**Na**				**neg**	**neg**	**neg**
18	**Al**				**neg**	**neg**	**neg**
19	**Tt, Al, Na**	**31.42 (0.90)**	**30.71 (0.41)**	**31.41 (0.77)**	**33.30**	**32.79**	**35.93**
20	**Tt, Al, Na**	**30.94 (0.33)**	**32.33 (0.64)**	**30.22 (0.62)**	**35.21**	**36.18**	**35.68**
4 v2	**Tt, Al**	**32.65 (1.48)**	**31.10 (0.84)**	**32.23 (1.58)**	**36.07**	**neg**	**36.52**
**10 v2**	**Na, Ad**				**neg**	**neg**	**neg**
27	**Al, Na, Ad**				**neg**	**36.76**	**neg**
12 v2	**Na**				**neg**	**neg**	**neg**
21	**Bioreclamation**				**neg**	**neg**	**neg**
22	**Bioreclamation**				**neg**	**neg**	**neg**
23	**Bioreclamation**				**neg**	**neg**	**neg**
24	**Bioreclamation**				**neg**	**neg**	**neg**
25	**Bioreclamation**				**neg**	**neg**	**neg**

Table A4.8: *T. trichiura Ct Scores for all aliquots and technical replicates of the final standard panel tested at Smith and Quantigen. Note where one aliquot is shown no amplification was observed in all other aliquots and replicates.*

Sample ID		*Trichuris trichiura*								
		Smith				Quantigen				
	Aliquot	Rep #1	Rep #2	Rep #3	Rep #4	Aliquot	Rep #1	Rep #2	Rep #3	Rep #4
1	A					D				
2	A					D				
7	A					D				
12v2	A					D				
14	A					D				
15	A					D				
18	A					D				
19	A	32.2	30.5	32.2	30.8	D	34.1	32.6	33.0	33.5
	B	30.4	31.3	30.5	30.6	E		33.1	32.5	32.7
	C	32.2	31.8	30.6	31.0	F	32.8	35.8	39.7	35.4
21	A					A				
22	A					A				
23	A					A				
24	A					A				
25	A					A				
27	A					D				
	B					E			36.76244	
	C					F				
NewTt_4	A	26.6	26.3	27.0	26.2	D	32.8	33.0	32.3	32.0
	B	25.9	25.6	26.0	25.7	E	30.0	30.1	30.3	30.1
	C	26.5	26.6	26.5	26.6	F	30.2	30.1	30.7	30.3
NewTt_5	A	26.2	25.6	26.0	26.0	D	30.4	30.2	30.0	30.9
	B	25.1	25.0	25.1	24.8	E	30.3	29.8	29.9	29.9
	C	25.3	25.5	25.5	25.4	F	31.1	31.4	31.3	31.0
New Tt_6	A	26.1	26.0	25.9	25.9	D	31.0	31.5	30.8	30.9
	B	26.1	26.2	26.3	25.8	E	29.4	29.3	29.2	29.2
	C	26.8	26.4	26.1	26.4	F	29.2	29.4	29.3	29.3
New Tt_7	A	26.4	26.2	26.5	26.2	D	29.2	30.8	30.1	30.8
	B	27.3	27.4	27.1	27.3	E	32.4	32.4	32.4	32.0
	C	26.0	26.0	26.2	26.4	F	29.8	29.9	30.0	29.8

Table A4. 9: *Initial Validation Panel, Smith vs. Quantigen Aliquot-Level* ***N. americanus Assay***
Note that Smith College Ct Scores provide the standard deviation in parentheses.

		N. americanus Ct (Mean- 4 Replicates					
Smith Panel ID	Sample Summary Status	Smith College			Quantigen		
		Aliquot A	Aliquot B	Aliquot C	Aliquot Q1	Aliquot Q2	Aliquot Q3
1	Al, Na	22.13 (0.08)	23.61 (0.11)	20.99 (0.14)	21.31	21.68	23.00
2	Na	23.93 (0.03)	25.48 (0.29)	22.43 (0.06)	27.21	29.68	26.13
3	Tt				neg	neg	neg
7	Al				neg	neg	neg
8	Tt, Al, Na	24.13 (0.03)	21.04 (0.27)	26.17 (0.21)	22.79	32.59	29.36
9	Tt, Al, Na	24.97 (1.06)	25.32 (0.31)	23.11 (0.44)	30.12	26.38	29.61
11	Tt, Na	23.72 (0.04)	25.76 (0.14)	25.25 (0.18)	29.92	25.57	31.64
12	Na	28.17 (0.10)	28.88 (0.10)	35.05 (2.55)	33.88	34.44	34.97
13	Tt				neg	neg	neg
14	Al				neg	neg	neg
15	Al, Na	22.33 (0.21)	25.19 (0.16)	25.72 (0.14)	27.46	28.63	28.30
16	Tt, Al				neg	neg	neg
17	Na	35.35 (2.17)	24.38 (0.14)	26.48 (0.07)	27.05	36.51	37.85
18	Al				neg	neg	neg
19	Tt, Al, Na	20.90 (0.10)	21.81 (0.09)	20.97 (0.10)	21.81	23.48	22.12
20	Tt, Al, Na	22.52 (0.08)	22.65 (0.09)	23.31 (0.12)	24.29	26.49	26.06
4 v2	Tt, Al				neg	neg	neg
10 v2	Na, Ad	22.20 (0.24)	22.11 (0.21)	19.75 (0.06)	25.02	26.43	26.79
27	Al, Na, Ad	19.58 (0.19)	19.03 (0.15)	19.63 (0.11)	27.60	27.43	27.47
12 v2	Na	25.8 (0.17)	25.0 (0.21)	26.2 (0.32)	28.29	27.24	27.44
21	Bioreclamation				neg	neg	neg
22	Bioreclamation				neg	neg	neg
23	Bioreclamation				neg	neg	neg
24	Bioreclamation				neg	neg	neg
25	Bioreclamation				neg	neg	neg

Table A4.10: *A. lumbricoides Ct Scores for all aliquots and technical replicates of the final standard panel tested at Smith and Quantigen. Note where one aliquot is shown no amplification was observed in all other aliquots and replicates.*

Sample ID		*Ascaris lumbricoides*								
		Smith					Quantigen			
	Aliquot	Rep #1	Rep #2	Rep #3	Rep #4	Aliquot	Rep #1	Rep #2	Rep #3	Rep #4
1	A	20.0	19.9	20.0	19.8	D	23.4	23.4	23.6	23.3
	B	21.8	21.8	22.2	21.9	E	25.1	26.9	26.3	25.3
	C	21.1	21.2	21.1	21.7	F	24.4	24.6	24.5	24.6
2	A					D				
7	A	15.4	16.4	16.4	16.4	D	22.7	21.1	21.3	20.9
	B	16.2	16.2	16.4	16.5	E	20.6	23.0	23.0	20.8
	C	16.4	16.4	16.1	16.5	F	22.6	22.2	21.9	21.7
12v2	A					D				
14	A	13.7	13.6	13.6	13.7	D	17.4	17.4	17.4	17.3
	B	13.5	13.5	13.6	13.6	E	15.6	15.7	15.7	17.5
	C	13.5	13.7	13.4	13.4	F	18.0	17.9	17.9	17.9
15	A	18.8	19.0	18.9	18.9	D	21.5	21.5	21.5	21.5
	B	18.1	18.2	18.0	18.0	E	21.4	21.5	21.4	22.5
	C	18.7	18.8	18.8	18.6	F	24.4	24.4	24.4	24.3
18	A	13.9	14.0	13.9	14.0	D	18.2	18.3	18.3	17.8
	B	14.8	14.9	14.9	14.8	E	29.5	17.7	18.0	17.8
	C	14.6	14.6	14.6	14.6	F	18.3	18.9	18.9	18.5
19	A	19.4	19.3	19.3	19.2	D	20.8	20.8	20.6	20.6
	B	18.3	18.4	18.5	18.5	E	25.7	21.0	21.2	20.8
	C	19.0	19.1	19.3	19.0	F	20.3	21.3	21.3	20.7
21	A					A				
22	A					A				
23	A					A				
24	A					A				
25	A					A				
27	A	12.8	12.8	12.8	12.8	D	17.0	17.0	17.3	17.6
	B	12.8	12.8	12.9	12.9	E	17.9	18.0	18.1	18.2
	C	13.3	13.4	13.3	13.3	F	18.4	19.3	18.6	18.5
NewTt_4	A					D	36.9			
	B	33.4				E				37.2
	C	33.1				F				
NewTt_5	A	12.5	12.5	12.7	12.5	D	16.7	16.5	16.6	16.6
	B	12.5	12.4	12.5	12.5	E	16.2	16.0	16.3	16.1
	C	12.2	12.0	12.2	12.2	F	16.0	16.0	15.9	16.0
New Tt_6	A	13.2	13.2	13.3	13.4	D	16.9	16.7	16.7	16.6
	B	13.0	13.0	13.0	12.9	E	15.9	16.0	16.2	16.0
	C	13.5	13.5	13.4	13.2	F	16.8	16.9	16.8	16.9
New Tt_7	A	13.4	13.3	13.3	13.3	D	16.2	16.3	16.2	16.1
	B	12.9	13.0	12.8	12.7	E	15.6	15.7	15.7	15.7
	C	13.5	13.3	13.3	13.5	F	16.5	16.7	16.6	16.6

Table A4. 11: *N. americanus Ct Scores for all aliquots and technical replicates of the final standard panel tested at Smith and Quantigen. Note where one aliquot is shown no amplification was observed in all other aliquots and replicates.*

		Necator americanus								
Sample ID		Smith				Quantigen				
	Aliquot	Rep #1	Rep #2	Rep #3	Rep #4	Aliquot	Rep #1	Rep #2	Rep #3	Rep #4
1	A	22.1	22.2	22.1	22.1	D	21.3	21.3	21.3	21.4
	B	23.5	23.6	23.6	23.8	E	21.9	21.4	21.5	21.9
	C	21.2	21.1	20.9	20.9	F	23.1	22.8	23.1	23.0
2	A	23.9	23.9	24.0	23.9	D	27.2	27.1	27.5	27.1
	B	25.9	25.2	25.4	25.4	E	29.6	29.5	29.9	29.7
	C	22.5	22.4	22.4	22.3	F	26.0	26.5	25.8	26.2
7	A					D				
12v2	A	25.8	26.0	25.8	25.5	D	28.5	28.1	28.3	28.2
	B	25.1	25.0	24.7	25.2	E	27.4	27.5	26.9	27.2
	C	26.0	26.7	26.0	26.3	F	27.5	27.5	27.4	27.4
14	A					D				
15	A	22.4	22.4	22.5	22.0	D	27.5	27.5	27.4	27.5
	B	25.2	25.0	25.4	25.1	E	28.4	28.6	28.7	28.8
	C	25.7	25.8	25.8	25.5	F		28.7	27.9	28.4
18	A					D				
19	A	20.8	21.0	20.9	20.8	D	21.7	21.7	21.9	22.0
	B	21.8	21.9	21.8	21.8	E		23.6	23.4	23.4
	C	20.9	21.1	20.9	21.0	F	22.0	22.1	22.2	22.1
21	A					A				
22	A					A				
23	A					A				
24	A					A				
25	A					A				
27	A	19.3	19.7	19.7	19.6	D	27.2	27.8	27.5	27.8
	B	18.9	18.9	19.1	19.2	E	27.4	27.5	27.3	27.5
	C	19.6	19.7	19.5	19.7	F	27.5	27.4	27.6	27.4
NewTt_4	A	17.8	17.6	17.8	17.7	D	25.6	25.7	25.5	25.8
	B	20.4	20.3	20.3	19.8	E	21.7	21.8	21.5	21.7
	C	20.0	19.9	19.5	20.0	F	22.9	22.9	23.0	23.3
NewTt_5	A	18.6	19.0	19.2	19.0	D	25.1	25.1	25.2	25.3
	B	18.7	18.7	19.0	18.4	E	21.9	21.9	21.7	21.8
	C	18.1	18.1	17.7	17.8	F	21.9	21.8	22.0	22.3
New Tt_6	A	20.1	19.6	20.0	20.1	D	22.6	22.6	22.6	22.7
	B	18.6	18.4	18.3	18.5	E	21.2	21.3	21.2	21.2
	C	18.0	18.3	17.6	17.8	F	22.1	22.1	22.2	22.4
New Tt_7	A				34.4	D				
	B					E	29.3	29.8	29.8	29.7
	C					F			37.0	

Table A4. 12: *A. duodenale Ct Scores for all aliquots and technical replicates of the final standard panel tested at Smith and Quantigen. Note where one aliquot is shown no amplification was observed in all other aliquots and replicates.*

Sample ID		*Ancylostoma duodenale*								
		Smith				Quantigen				
	Aliquot	Rep #1	Rep #2	Rep #3	Rep #4	Aliquot	Rep #1	Rep #2	Rep #3	Rep #4
1	A					D				
2	A					D				
7	A					D				
12v2	A					D				
14	A					D				
15	A					D				
18	A					D				
19	A					D				
21	A					A				
22	A					A				
23	A					A				
24	A					A				
25	A					A				
27	A	25.6	25.9	26.1	25.9	D	30.4	30.4	30.2	30.3
	B	26.2	26.3	26.0	26.3	E	30.2	30.3	31.3	32.8
	C	25.4	25.9	25.8	25.9	F	31.3	31.5	39.9	36.7
NewTt_4	A					D				
NewTt_5	A					D				
New Tt_6	A					D				
New Tt_7	A					D				

Table A4. 13: *Evaluation of Assay Accuracy by standards. (a) Calculation of Assay Accuracy at the Technical Replicate level. (b) Calculation of Assay Accuracy at the Aliquot level. TP = True Positive, TN = True Negative, FN = False Negative, FP = False Positive.*

(a)

Technical Replicate																				
					T. trichiura				*A. lumbricoides*				*N. americanus*				*A. duodenale*			
**Standard #**	**STH Status**				**TP**	**TN**	**FN**	**FP**	**TP**	**TN**	**FN**	**FP**	**TP**	**TN**	**FN**	**FP**	**TP**	**TN**	**FN**	**FP**
1		Al	Na			12			12				12					12		
2			Na			12				12			12					12		
7		Al				12			12					12				12		
12v2			Na			12				12			12					12		
14		Al				12			12					12				12		
15		Al	Na			12			12				11					12		
18		Al				12			12					12				12		
19	Tt	Al	Na		11				12				11					12		
21						12				12				12				12		
22						12				12				12				12		
23						12				12				12				12		
24						12				12				12				12		
25						12				12				12				12		
27		Al	Na	Ad		11		1	12				12				12			
NewTt_4	Tt	(Al)	Na		12				2	10			12					12		
NewTt_5	Tt	Al	Na		12				12				12					12		
NewTt_6	Tt	Al	Na		12				12				12					12		
NewTt_7	Tt	Al	(Na)		12				12				5	7				12		
				Totals	59	155	0	1	122	94	0	0	111	103	0	0	12	204	0	0
				Accuracy (Tech. Replicate)	99.5%				100.0%				100.0%				100.0%			

(b)

Individual Extraction																				
					T. trichiura				*A. lumbricoides*				*N. americanus*				*A. duodenale*			
**Standard #**	**STH Status**				**TP**	**TN**	**FN**	**FP**	**TP**	**TN**	**FN**	**FP**	**TP**	**TN**	**FN**	**FP**	**TP**	**TN**	**FN**	**FP**
1		Al	Na			3			3				3					3		
2			Na			3				3			3					3		
7		Al				3			3					3				3		
12v2			Na			3				3			3					3		
14		Al				3			3					3				3		
15		Al	Na			3			3				3					3		
18		Al				3			3					3				3		
19	Tt	Al	Na		3				3				3					3		
21						3				3				3				3		
22						3				3				3				3		
23						3				3				3				3		
24						3				3				3				3		
25						3				3				3				3		
27		Al	Na	Ad		2		1	3				3				3			
NewTt_4	Tt	(Al)	Na		3				3				3					3		
NewTt_5	Tt	Al	Na		3				3				3					3		
NewTt_6	Tt	Al	Na		3				3				3					3		
NewTt_7	Tt	Al	(Na)		3				3				3					3		
				Totals	15	38	0	1	33	21	0	0	30	24	0	0	3	51	0	0
				Accuracy (Individual Extract)	98.1%				100.0%				100.0%				100.0%			

Appendix A5: Evaluation of Assay Specificity (I.B.iii)

Table A5. 1: *Raw Ct Scores for four Benin Specificity Panel Samples identified STH-positive by Smith College DNA extraction and qPCR testing. Note each sample was tested in duplicate by qPCR.*

Original Smith Data (Ran in duplicate)				
**Sample ID**	***N. americanus***	***T. trichiura***	***A. lumbricoides***	***A. duodenale***
1	Undetermined	Undetermined	31.0	Undetermined
	Undetermined	Undetermined	30.5	Undetermined
2	27.8	Undetermined	Undetermined	Undetermined
	28.2	Undetermined	Undetermined	Undetermined
3	21.4	Undetermined	Undetermined	Undetermined
	21.2	Undetermined	Undetermined	Undetermined

Table A5. 2: *(a)* ***DeWorm3 Liquid Assay testing of Smith College DNA extracts*** *of the four Benin Specificity Panel Samples identified STH- positive by the Smith College Assay.* ***(b) DeWorm3 Lyophilized Assay testing of Smith College DNA extracts*** *of the four Benin Specificity Panel Samples identified STH-positive by the Smith College Assay.*

(a)

Experiment 1: Smith and Quantigen Extracts Ran in Triplicate on Liquid SMITH DATA ONLY				
**Sample ID**	**N. americanus**	**T. trichiura**	**A. lumbricoides**	**A. duodenale**
1	Undetermined	Undetermined	31.8	Undetermined
	Undetermined	Undetermined	32.2	Undetermined
	Undetermined	Undetermined	32.1	Undetermined
2	29.7	Undetermined	Undetermined	Undetermined
	30.0	Undetermined	Undetermined	Undetermined
	29.6	Undetermined	Undetermined	Undetermined
3	22.4	Undetermined	Undetermined	Undetermined
	22.4	Undetermined	Undetermined	Undetermined
	22.4	Undetermined	Undetermined	Undetermined

(b)

Experiment 2: Smith and Quantigen Extracts Ran in Triplicate on Lyo SMITH DATA ONLY				
**Sample ID**	**N. americanus**	**T. trichiura**	**A. lumbricoides**	**A. duodenale**
1	Undetermined	Undetermined	32.5	Undetermined
	Undetermined	Undetermined	33.8	Undetermined
	Undetermined	Undetermined	33.9	Undetermined
2	29.6	Undetermined	Undetermined	Undetermined
	29.1	Undetermined	Undetermined	Undetermined
	30.1	Undetermined	Undetermined	Undetermined
3	23.0	Undetermined	Undetermined	Undetermined
	22.4	Undetermined	Undetermined	Undetermined
	22.9	Undetermined	Undetermined	Undetermined

Table A5. 3: ***(a) Re-testing by DeWorm3 Liquid Assay of the Quantigen Extracts*** *of the three Benin Specificity Panel Samples identified STH- positive by the Smith College Assay.* ***(b) Re-testing by DeWorm3 Lyophilized Assay of the Quantigen Extracts*** *of the three Benin Specificity Panel Samples identified STH-positive by the Smith College Assay.*

(a)

Experiment 1: Smith and Quantigen Extracts Ran in Triplicate on Liquid QUANTIGEN DATA ONLY				
Sample ID	N. americanus	T. trichiura	A. lumbricoides	A. duodenale
1	Undetermined	Undetermined	34.0	Undetermined
	Undetermined	Undetermined	36.2	Undetermined
	Undetermined	Undetermined	37.5	Undetermined
2	Undetermined	Undetermined	Undetermined	Undetermined
	Undetermined	Undetermined	Undetermined	Undetermined
	Undetermined	Undetermined	Undetermined	Undetermined
3	Undetermined	Undetermined	Undetermined	Undetermined
	Undetermined	Undetermined	Undetermined	Undetermined
	Undetermined	Undetermined	Undetermined	Undetermined

(b)

Experiment 2: Smith and Quantigen Extracts Ran in Triplicate on Lyo QUANTIGEN DATA ONLY				
Sample ID	N. americanus	T. trichiura	A. lumbricoides	A. duodenale
1	Undetermined	Undetermined	37.2	Undetermined
	Undetermined	Undetermined	38.7	Undetermined
	Undetermined	Undetermined	36.8	Undetermined
2	Undetermined	Undetermined	Undetermined	Undetermined
	Undetermined	Undetermined	Undetermined	Undetermined
	Undetermined	Undetermined	Undetermined	Undetermined
3	Undetermined	Undetermined	Undetermined	Undetermined
	Undetermined	Undetermined	Undetermined	Undetermined
	Undetermined	Undetermined	Undetermined	Undetermined

Table A5. 4*: Re-extraction and qPCR testing by the DeWorm3 procedures at Quantigen of the three Benin Specificity Panel Samples identified STH-positive by Smith College Assay. Note three new aliquots from each sample were extracted in singlet qPCR testing, thus each row represents a separate aliquot.*

Experiment 3: Original Samples Re-extracted in Triplicate (Quantigen Ext'n Method)				
Sample ID	*N. americanus*	*T. trichirua*	*A. lumbricoides*	*A. duodenale*
1	Undetermined	Undetermined	Undetermined	Undetermined
	Undetermined	Undetermined	36.9	Undetermined
	Undetermined	Undetermined	34.3	Undetermined
2	31.3	Undetermined	Undetermined	Undetermined
	Undetermined	Undetermined	Undetermined	Undetermined
	31.3	Undetermined	Undetermined	Undetermined
3	30.1	Undetermined	Undetermined	Undetermined
	28.2	Undetermined	Undetermined	Undetermined
	Undetermined	Undetermined	Undetermined	Undetermined

Table A5. 5: *Re-testing of the five discordant DNA extracts from CMC by the Lyophilized DeWorm3 Assay.*

Re-qPCR of Original CMC DNA at Quantigen					
Sample ID	***Replicate***	***N. americanus***	***T. trichiura***	***A. lumbricoides***	***A. duodenale***
**1**	**a**	21.6	Undetermined	Undetermined	Undetermined
	**b**	21.2	Undetermined	Undetermined	Undetermined
	**c**	21.3	Undetermined	Undetermined	Undetermined
**2**	**a**	19.6	Undetermined	Undetermined	Undetermined
	**b**	20.0	Undetermined	Undetermined	Undetermined
	**c**	19.8	Undetermined	Undetermined	Undetermined
**3**	**a**	31.0	Undetermined	Undetermined	Undetermined
	**b**	31.2	Undetermined	Undetermined	Undetermined
	**c**	31.0	Undetermined	Undetermined	Undetermined
**4**	**a**	36.9	Undetermined	Undetermined	Undetermined
	**b**	Undetermined	Undetermined	Undetermined	Undetermined
	**c**	32.7	Undetermined	Undetermined	Undetermined
**5**	**a**	25.0	Undetermined	Undetermined	Undetermined
	**b**	25.2	Undetermined	Undetermined	Undetermined
	**c**	26.6	Undetermined	Undetermined	Undetermined

Table A5. 6: *Re-testing of the five discordant Quantigen DNA extracts by the Lyophilized DeWorm3 Assay.*

Re-qPCR of Original Quantigen DNA					
Sample ID	***Replicate***	***N. americanus***	***T. trichiura***	***A. lumbricoides***	***A. duodenale***
**1**	**a**	Undetermined	Undetermined	Undetermined	Undetermined
	**b**	Undetermined	Undetermined	Undetermined	Undetermined
	**c**	Undetermined	Undetermined	Undetermined	Undetermined
**2**	**a**	Undetermined	Undetermined	Undetermined	Undetermined
	**b**	Undetermined	Undetermined	Undetermined	Undetermined
	**c**	Undetermined	Undetermined	Undetermined	Undetermined
**3**	**a**	Undetermined	Undetermined	Undetermined	Undetermined
	**b**	Undetermined	Undetermined	Undetermined	Undetermined
	**c**	Undetermined	Undetermined	Undetermined	Undetermined
**4**	**a**	Undetermined	Undetermined	Undetermined	Undetermined
	**b**	Undetermined	Undetermined	Undetermined	Undetermined
	**c**	Undetermined	Undetermined	Undetermined	Undetermined
**5**	**a**	Undetermined	Undetermined	Undetermined	Undetermined
	**b**	Undetermined	Undetermined	Undetermined	Undetermined
	**c**	Undetermined	Undetermined	Undetermined	Undetermined

Table A5. 7: *Re-extraction of the 5 discordant India samples at Quantigen. Each sample extract was testing in duplicate.*

**Re-Extraction of Samples at Quantigen**						
Sample ID	***Extraction***	***Replicate***	***N. americanus***	***T. trichiura***	***A. lumbricoides***	***A. duodenale***
**1**	1	a	**26.3**	Undetermined	Undetermined	Undetermined
		b	**26.7**	Undetermined	Undetermined	Undetermined
	2	a	**N/A**	Undetermined	Undetermined	Undetermined
		b	**N/A**	Undetermined	Undetermined	Undetermined
**2**	1	a	**27.2**	Undetermined	Undetermined	Undetermined
		b	**26.9**	Undetermined	Undetermined	Undetermined
	2	a	**25.4**	Undetermined	Undetermined	Undetermined
		b	**24.8**	Undetermined	Undetermined	Undetermined
**3**	1	a	**Undetermined**	Undetermined	Undetermined	Undetermined
		b	**Undetermined**	Undetermined	Undetermined	Undetermined
	2	a	**29.1**	Undetermined	Undetermined	Undetermined
		b	**29.3**	Undetermined	Undetermined	Undetermined
**4**	1	a	**36.7**	Undetermined	Undetermined	Undetermined
		b	**36.0**	Undetermined	Undetermined	Undetermined
	2	a	**N/A**	Undetermined	Undetermined	Undetermined
		b	**N/A**	Undetermined	Undetermined	Undetermined
**5**	1	a	**Undetermined**	Undetermined	Undetermined	Undetermined
		b	**Undetermined**	Undetermined	Undetermined	Undetermined
	2	a	**30.3**	Undetermined	Undetermined	Undetermined
		b	**30.2**	Undetermined	Undetermined	Undetermined

Appendix A6: Raw Data: Assay Sensitivity/LOD (I.B.iv)

***Table A6. 1**: Limit of detection for STH. Each STH standard was serially diluted and qPCR was performed (a) 1:10 Serial Dilution Results (b) 1:2 Serial Dilution Results. Green highlighting indicates that the dilution yielded CT scores for all technical replicates. Red indicates the run contained at least 1 undeterminable Ct score. Yellow highlighting indicates the dilution from which to begin the 1:2 serial. Black indicates that the dilution was not run.*

(a)

First round of dilutions (1:10)						
**Sample**	First Dilution Point	2nd	3rd	4th	5th	6th
**N. americanus** – StdExt1_6_Na	1.00E-01	1.00E-02	1.00E-03	1.00E-04	1.00E-05	1.00E-06
**Positives**	10/10	10/10	10/10	10/10	3/10	0/10
**A. lumbricoides** – StdExt1_6_Al	1.00E-01	1.00E-02	1.00E-03	1.00E-04	1.00E-05	1.00E-06
**Positives**	10/10	10/10	10/10	8/10	2/10	1/10
**A. duodenale** – StdExt1_208_Ad	1.00E-01	1.00E-02	1.00E-03	1.00E-04	1.00E-05	1.00E-06
**Positives**	10/10	9/10	1/10	0/10	0/10	0/10
**T. trichiura** – StdExt2_44_Tt	1.00E-01	1.00E-02	1.00E-03	1.00E-04	1.00E-05	1.00E-06
**Positives**	10/10	10/10	10/10	9/10	0/10	

Key	
Aliquot ID	*Standard #*
StdExt1_6	1
StdExt1_208	27
StdExt2_44	19

(b)

Second round of dilutions (1:2)							
**Sample**	First Dilution Point	2nd	3rd	4th	5th	6th	7th
**N. americanus** – StdExt1_6_Na	1.00E-04	5.00E-05	2.50E-05	1.25E-05			
**Positives**	20/20	20/20	20/20	18/20			
**A. lumbricoides** – StdExt1_6_Al	1.00E-03	5.00E-04	2.50E-04	1.25E-04	6.25E-05	3.13E-05	1.56E-05
**Positives**	20/20	20/20	20/20	20/20	17/20	4/20	4/20
**A. duodenale** – StdExt1_208_Ad	1.00E-01	5.00E-02	2.50E-02	1.25E-02	6.25E-03	3.13E-03	1.56E-03
**Positives**	20/20	20/20	20/20	20/20	15/20	11/20	9/20
**T. trichiura** – StdExt2_44_Tt	1.00E-03	5.00E-04	2.50E-04	1.25E-04	6.25E-05	3.13E-05	1.56E-05
**Positives**	20/20	20/20	20/20	19/20	16/20	10/20	7/20

***Table A6. 2**: 1:10 Serial Dilution Ct Scores for N. americanus. Note there are 10 replicates of each dilution point.*

First Dilution (1:10)					
Well	Sample Name	Target Name	CT		
1	StdExt1_6_Na_0.1_1	N. americanus	22.596		
10	StdExt1_6_Na_0.1_10	N. americanus	21.807		
2	StdExt1_6_Na_0.1_2	N. americanus	21.788		
3	StdExt1_6_Na_0.1_3	N. americanus	21.742		
4	StdExt1_6_Na_0.1_4	N. americanus	22.392		
5	StdExt1_6_Na_0.1_5	N. americanus	21.704		
6	StdExt1_6_Na_0.1_6	N. americanus	22.286		
7	StdExt1_6_Na_0.1_7	N. americanus	21.698		
8	StdExt1_6_Na_0.1_8	N. americanus	22.163		
9	StdExt1_6_Na_0.1_9	N. americanus	21.784	1.00E-01	1st Dilution
49	StdExt1_6_Na_0.01_1	N. americanus	25.696		
58	StdExt1_6_Na_0.01_10	N. americanus	24.741		
50	StdExt1_6_Na_0.01_2	N. americanus	25.620		
51	StdExt1_6_Na_0.01_3	N. americanus	25.859		
52	StdExt1_6_Na_0.01_4	N. americanus	24.781		
53	StdExt1_6_Na_0.01_5	N. americanus	26.013		
54	StdExt1_6_Na_0.01_6	N. americanus	24.899		
55	StdExt1_6_Na_0.01_7	N. americanus	25.224		
56	StdExt1_6_Na_0.01_8	N. americanus	24.996		
57	StdExt1_6_Na_0.01_9	N. americanus	25.071	1.00E-02	2nd Dilution
97	StdExt1_6_Na_0.001_1	N. americanus	28.668		
106	StdExt1_6_Na_0.001_10	N. americanus	29.081		
98	StdExt1_6_Na_0.001_2	N. americanus	28.961		
99	StdExt1_6_Na_0.001_3	N. americanus	28.928		
100	StdExt1_6_Na_0.001_4	N. americanus	28.121		
101	StdExt1_6_Na_0.001_5	N. americanus	29.189		
102	StdExt1_6_Na_0.001_6	N. americanus	28.148		
103	StdExt1_6_Na_0.001_7	N. americanus	29.131		
104	StdExt1_6_Na_0.001_8	N. americanus	28.790		
105	StdExt1_6_Na_0.001_9	N. americanus	27.991	1.00E-03	3rd Dilution
145	StdExt1_6_Na_0.0001_1	N. americanus	31.092		
154	StdExt1_6_Na_0.0001_10	N. americanus	31.348		
146	StdExt1_6_Na_0.0001_2	N. americanus	31.89536095		
147	StdExt1_6_Na_0.0001_3	N. americanus	31.308		
148	StdExt1_6_Na_0.0001_4	N. americanus	35.81274796		
149	StdExt1_6_Na_0.0001_5	N. americanus	31.985		
150	StdExt1_6_Na_0.0001_6	N. americanus	32.653		
151	StdExt1_6_Na_0.0001_7	N. americanus	32.847		
152	StdExt1_6_Na_0.0001_8	N. americanus	33.163		
153	StdExt1_6_Na_0.0001_9	N. americanus	31.540	1.00E-04	4th Dilution
193	StdExt1_6_Na_0.00001_1	N. americanus	30.832		
202	StdExt1_6_Na_0.00001_10	N. americanus	Undetermined		
194	StdExt1_6_Na_0.00001_2	N. americanus	Undetermined		
195	StdExt1_6_Na_0.00001_3	N. americanus	Undetermined		
196	StdExt1_6_Na_0.00001_4	N. americanus	Undetermined		
197	StdExt1_6_Na_0.00001_5	N. americanus	36.29724503		
198	StdExt1_6_Na_0.00001_6	N. americanus	Undetermined		
199	StdExt1_6_Na_0.00001_7	N. americanus	Undetermined		
200	StdExt1_6_Na_0.00001_8	N. americanus	36.0435257		
201	StdExt1_6_Na_0.00001_9	N. americanus	Undetermined	1.00E-05	5th Dilution
241	StdExt1_6_Na_0.000001_1	N. americanus	Undetermined		
250	StdExt1_6_Na_0.000001_10	N. americanus	Undetermined		
242	StdExt1_6_Na_0.000001_2	N. americanus	Undetermined		
243	StdExt1_6_Na_0.000001_3	N. americanus	Undetermined		
244	StdExt1_6_Na_0.000001_4	N. americanus	Undetermined		
245	StdExt1_6_Na_0.000001_5	N. americanus	Undetermined		
246	StdExt1_6_Na_0.000001_6	N. americanus	Undetermined		
247	StdExt1_6_Na_0.000001_7	N. americanus	Undetermined		
248	StdExt1_6_Na_0.000001_8	N. americanus	Undetermined		
249	StdExt1_6_Na_0.000001_9	N. americanus	Undetermined	1.00E-06	6th Dilution

***Table A6. 3**: 1:2 Serial Dilution Ct Scores for N. americanus. Note there are 20 replicates of each dilution point.*

Second Dilution (1:2)					
Well	Sample Name	Target Name	CT		
23	NTC	N. americanus	Undetermined		
24	Pos. PCR	N. americanus	26.950		
1	StdExt1_6_Na_D1_R1	N. americanus	30.290	1.00E-04	1st Dilution
19	StdExt1_6_Na_D1_R10	N. americanus	30.510		
2	StdExt1_6_Na_D1_R11	N. americanus	30.302		
4	StdExt1_6_Na_D1_R12	N. americanus	30.999		
6	StdExt1_6_Na_D1_R13	N. americanus	31.616		
8	StdExt1_6_Na_D1_R14	N. americanus	31.217		
10	StdExt1_6_Na_D1_R15	N. americanus	31.905		
12	StdExt1_6_Na_D1_R16	N. americanus	31.162		
14	StdExt1_6_Na_D1_R17	N. americanus	31.200		
16	StdExt1_6_Na_D1_R18	N. americanus	30.342		
18	StdExt1_6_Na_D1_R19	N. americanus	30.876		
3	StdExt1_6_Na_D1_R2	N. americanus	31.453		
20	StdExt1_6_Na_D1_R20	N. americanus	31.631		
5	StdExt1_6_Na_D1_R3	N. americanus	31.597		
7	StdExt1_6_Na_D1_R4	N. americanus	31.240		
9	StdExt1_6_Na_D1_R5	N. americanus	30.940		
11	StdExt1_6_Na_D1_R6	N. americanus	30.964		
13	StdExt1_6_Na_D1_R7	N. americanus	30.592		
15	StdExt1_6_Na_D1_R8	N. americanus	31.330		
17	StdExt1_6_Na_D1_R9	N. americanus	32.218		
49	StdExt1_6_Na_D2_R1	N. americanus	32.909	5.00E-05	2nd Dilution
67	StdExt1_6_Na_D2_R10	N. americanus	33.023		
50	StdExt1_6_Na_D2_R11	N. americanus	32.564		
52	StdExt1_6_Na_D2_R12	N. americanus	32.635		
54	StdExt1_6_Na_D2_R13	N. americanus	34.877		
56	StdExt1_6_Na_D2_R14	N. americanus	31.207		
58	StdExt1_6_Na_D2_R15	N. americanus	31.582		
60	StdExt1_6_Na_D2_R16	N. americanus	33.777		
62	StdExt1_6_Na_D2_R17	N. americanus	32.335		
64	StdExt1_6_Na_D2_R18	N. americanus	31.951		
66	StdExt1_6_Na_D2_R19	N. americanus	33.287		
51	StdExt1_6_Na_D2_R2	N. americanus	32.747		
68	StdExt1_6_Na_D2_R20	N. americanus	32.224		
53	StdExt1_6_Na_D2_R3	N. americanus	31.441		
55	StdExt1_6_Na_D2_R4	N. americanus	33.075		
57	StdExt1_6_Na_D2_R5	N. americanus	31.952		
59	StdExt1_6_Na_D2_R6	N. americanus	33.297		
61	StdExt1_6_Na_D2_R7	N. americanus	32.993		
63	StdExt1_6_Na_D2_R8	N. americanus	31.082		
65	StdExt1_6_Na_D2_R9	N. americanus	32.950		
97	StdExt1_6_Na_D3_R1	N. americanus	32.718	2.50E-05	3rd Dilution
115	StdExt1_6_Na_D3_R10	N. americanus	33.694		
98	StdExt1_6_Na_D3_R11	N. americanus	31.805		
100	StdExt1_6_Na_D3_R12	N. americanus	35.434		
102	StdExt1_6_Na_D3_R13	N. americanus	32.817		
104	StdExt1_6_Na_D3_R14	N. americanus	33.682		
106	StdExt1_6_Na_D3_R15	N. americanus	33.135		
108	StdExt1_6_Na_D3_R16	N. americanus	34.605		
110	StdExt1_6_Na_D3_R17	N. americanus	34.548		
112	StdExt1_6_Na_D3_R18	N. americanus	35.765		
114	StdExt1_6_Na_D3_R19	N. americanus	32.246		
99	StdExt1_6_Na_D3_R2	N. americanus	31.883		
116	StdExt1_6_Na_D3_R20	N. americanus	32.658		
101	StdExt1_6_Na_D3_R3	N. americanus	33.425		
103	StdExt1_6_Na_D3_R4	N. americanus	38.347		
105	StdExt1_6_Na_D3_R5	N. americanus	32.610		
107	StdExt1_6_Na_D3_R6	N. americanus	33.578		
109	StdExt1_6_Na_D3_R7	N. americanus	32.713		
111	StdExt1_6_Na_D3_R8	N. americanus	32.915		
113	StdExt1_6_Na_D3_R9	N. americanus	33.874		

Second Dilution (1:2)					
Well	Sample Name	Target Name	CT		
145	StdExt1_6_Na_D4_R1	N. americanus	33.585	1.25E-05	4th Dilution
163	StdExt1_6_Na_D4_R10	N. americanus	32.114		
146	StdExt1_6_Na_D4_R11	N. americanus	35.518		
148	StdExt1_6_Na_D4_R12	N. americanus	34.381		
150	StdExt1_6_Na_D4_R13	N. americanus	35.530		
152	StdExt1_6_Na_D4_R14	N. americanus	34.630		
154	StdExt1_6_Na_D4_R15	N. americanus	35.581		
156	StdExt1_6_Na_D4_R16	N. americanus	33.914		
158	StdExt1_6_Na_D4_R17	N. americanus	36.208		
160	StdExt1_6_Na_D4_R18	N. americanus	34.616		
162	StdExt1_6_Na_D4_R19	N. americanus	34.376		
147	StdExt1_6_Na_D4_R2	N. americanus	33.340		
164	StdExt1_6_Na_D4_R20	N. americanus	34.052		
149	StdExt1_6_Na_D4_R3	N. americanus	Undetermined		
151	StdExt1_6_Na_D4_R4	N. americanus	32.252		
153	StdExt1_6_Na_D4_R5	N. americanus	32.530		
155	StdExt1_6_Na_D4_R6	N. americanus	31.538		
157	StdExt1_6_Na_D4_R7	N. americanus	35.085		
159	StdExt1_6_Na_D4_R8	N. americanus	34.682		
161	StdExt1_6_Na_D4_R9	N. americanus	Undetermined		

Table A6. 4: *1:2 Serial Dilution Ct Scores for A. lumbricoides. Note there are 10 replicates of each dilution point.*

First Dilution (1:10)					
Well	Sample Name	Target Name	CT		
25	StdExt1_6_Al_0.1_1	A.lumbricoides	22.880		
34	StdExt1_6_Al_0.1_10	A.lumbricoides	23.029		
26	StdExt1_6_Al_0.1_2	A.lumbricoides	23.886		
27	StdExt1_6_Al_0.1_3	A.lumbricoides	23.215		
28	StdExt1_6_Al_0.1_4	A.lumbricoides	23.744		
29	StdExt1_6_Al_0.1_5	A.lumbricoides	23.168		
30	StdExt1_6_Al_0.1_6	A.lumbricoides	27.280		
31	StdExt1_6_Al_0.1_7	A.lumbricoides	23.156		
32	StdExt1_6_Al_0.1_8	A.lumbricoides	23.856		
33	StdExt1_6_Al_0.1_9	A.lumbricoides	23.259	1.00E-01	1st Dilution
73	StdExt1_6_Al_0.01_1	A.lumbricoides	26.410		
82	StdExt1_6_Al_0.01_10	A.lumbricoides	26.905		
74	StdExt1_6_Al_0.01_2	A.lumbricoides	26.037		
75	StdExt1_6_Al_0.01_3	A.lumbricoides	26.340		
76	StdExt1_6_Al_0.01_4	A.lumbricoides	25.949		
77	StdExt1_6_Al_0.01_5	A.lumbricoides	25.986		
78	StdExt1_6_Al_0.01_6	A.lumbricoides	26.292		
79	StdExt1_6_Al_0.01_7	A.lumbricoides	25.980		
80	StdExt1_6_Al_0.01_8	A.lumbricoides	28.906		
81	StdExt1_6_Al_0.01_9	A.lumbricoides	26.505	1.00E-02	2nd Dilution
121	StdExt1_6_Al_0.001_1	A.lumbricoides	30.009		
130	StdExt1_6_Al_0.001_10	A.lumbricoides	30.876		
122	StdExt1_6_Al_0.001_2	A.lumbricoides	30.349		
123	StdExt1_6_Al_0.001_3	A.lumbricoides	28.970		
124	StdExt1_6_Al_0.001_4	A.lumbricoides	29.121		
125	StdExt1_6_Al_0.001_5	A.lumbricoides	30.860		
126	StdExt1_6_Al_0.001_6	A.lumbricoides	30.264		
127	StdExt1_6_Al_0.001_7	A.lumbricoides	30.362		
128	StdExt1_6_Al_0.001_8	A.lumbricoides	29.713		
129	StdExt1_6_Al_0.001_9	A.lumbricoides	30.745	1.00E-03	3rd Dilution
169	StdExt1_6_Na_0.0001_1	A.lumbricoides	34.878		
178	StdExt1_6_Na_0.0001_10	A.lumbricoides	35.157		
170	StdExt1_6_Na_0.0001_2	A.lumbricoides	Undetermined		
171	StdExt1_6_Na_0.0001_3	A.lumbricoides	35.434		
172	StdExt1_6_Na_0.0001_4	A.lumbricoides	Undetermined		
173	StdExt1_6_Na_0.0001_5	A.lumbricoides	35.858		
174	StdExt1_6_Na_0.0001_6	A.lumbricoides	39.186		
175	StdExt1_6_Na_0.0001_7	A.lumbricoides	38.760		
176	StdExt1_6_Na_0.0001_8	A.lumbricoides	37.116		
177	StdExt1_6_Na_0.0001_9	A.lumbricoides	39.221	1.00E-04	4th Dilution
217	StdExt1_6_Al_0.00001_1	A.lumbricoides	27.074		
226	StdExt1_6_Al_0.00001_10	A.lumbricoides	Undetermined		
218	StdExt1_6_Al_0.00001_2	A.lumbricoides	32.781		
219	StdExt1_6_Al_0.00001_3	A.lumbricoides	Undetermined		
220	StdExt1_6_Al_0.00001_4	A.lumbricoides	Undetermined		
221	StdExt1_6_Al_0.00001_5	A.lumbricoides	Undetermined		
222	StdExt1_6_Al_0.00001_6	A.lumbricoides	Undetermined		
223	StdExt1_6_Al_0.00001_7	A.lumbricoides	Undetermined		
224	StdExt1_6_Al_0.00001_8	A.lumbricoides	Undetermined		
225	StdExt1_6_Al_0.00001_9	A.lumbricoides	Undetermined	1.00E-05	5th Dilution
265	StdExt1_6_Al_0.000001_1	A.lumbricoides	Undetermined		
274	StdExt1_6_Al_0.000001_10	A.lumbricoides	Undetermined		
266	StdExt1_6_Al_0.000001_2	A.lumbricoides	Undetermined		
267	StdExt1_6_Al_0.000001_3	A.lumbricoides	Undetermined		
268	StdExt1_6_Al_0.000001_4	A.lumbricoides	37.743		
269	StdExt1_6_Al_0.000001_5	A.lumbricoides	Undetermined		
270	StdExt1_6_Al_0.000001_6	A.lumbricoides	Undetermined		
271	StdExt1_6_Al_0.000001_7	A.lumbricoides	Undetermined		
272	StdExt1_6_Al_0.000001_8	A.lumbricoides	Undetermined		
273	StdExt1_6_Al_0.000001_9	A.lumbricoides	Undetermined	1.00E-06	6th Dilution

Table A6. 5: *1:2 Serial Dilution Ct Scores for A. lumbricoides. Note there are 20 replicates of each dilution point.*

Second Dilution (1:2)					
Well	Sample Name	Target Name	CT		
25	StdExt1_6_Al_D1_R1	A.lumbricoides	30.474	1.00E-03	1st Dilution
43	StdExt1_6_Al_D1_R10	A.lumbricoides	30.462		
26	StdExt1_6_Al_D1_R11	A.lumbricoides	30.425		
28	StdExt1_6_Al_D1_R12	A.lumbricoides	30.024		
30	StdExt1_6_Al_D1_R13	A.lumbricoides	29.543		
32	StdExt1_6_Al_D1_R14	A.lumbricoides	30.062		
34	StdExt1_6_Al_D1_R15	A.lumbricoides	29.836		
36	StdExt1_6_Al_D1_R16	A.lumbricoides	30.571		
38	StdExt1_6_Al_D1_R17	A.lumbricoides	30.291		
40	StdExt1_6_Al_D1_R18	A.lumbricoides	29.929		
42	StdExt1_6_Al_D1_R19	A.lumbricoides	30.159		
27	StdExt1_6_Al_D1_R2	A.lumbricoides	29.355		
44	StdExt1_6_Al_D1_R20	A.lumbricoides	29.844		
29	StdExt1_6_Al_D1_R3	A.lumbricoides	29.202		
31	StdExt1_6_Al_D1_R4	A.lumbricoides	30.090		
33	StdExt1_6_Al_D1_R5	A.lumbricoides	28.853		
35	StdExt1_6_Al_D1_R6	A.lumbricoides	29.889		
37	StdExt1_6_Al_D1_R7	A.lumbricoides	29.960		
39	StdExt1_6_Al_D1_R8	A.lumbricoides	29.007		
41	StdExt1_6_Al_D1_R9	A.lumbricoides	30.490		
73	StdExt1_6_Al_D2_R1	A.lumbricoides	31.922	5.00E-04	2nd Dilution
91	StdExt1_6_Al_D2_R10	A.lumbricoides	30.870		
74	StdExt1_6_Al_D2_R11	A.lumbricoides	31.513		
76	StdExt1_6_Al_D2_R12	A.lumbricoides	31.591		
78	StdExt1_6_Al_D2_R13	A.lumbricoides	31.125		
80	StdExt1_6_Al_D2_R14	A.lumbricoides	31.877		
82	StdExt1_6_Al_D2_R15	A.lumbricoides	30.000		
84	StdExt1_6_Al_D2_R16	A.lumbricoides	30.874		
86	StdExt1_6_Al_D2_R17	A.lumbricoides	30.727		
88	StdExt1_6_Al_D2_R18	A.lumbricoides	30.458		
90	StdExt1_6_Al_D2_R19	A.lumbricoides	31.793		
75	StdExt1_6_Al_D2_R2	A.lumbricoides	30.998		
92	StdExt1_6_Al_D2_R20	A.lumbricoides	29.650		
77	StdExt1_6_Al_D2_R3	A.lumbricoides	32.154		
79	StdExt1_6_Al_D2_R4	A.lumbricoides	31.356		
81	StdExt1_6_Al_D2_R5	A.lumbricoides	32.255		
83	StdExt1_6_Al_D2_R6	A.lumbricoides	30.740		
85	StdExt1_6_Al_D2_R7	A.lumbricoides	31.506		
87	StdExt1_6_Al_D2_R8	A.lumbricoides	31.110		
89	StdExt1_6_Al_D2_R9	A.lumbricoides	32.324		
121	StdExt1_6_Al_D3_R1	A.lumbricoides	33.847	2.50E-04	3rd Dilution
139	StdExt1_6_Al_D3_R10	A.lumbricoides	31.042		
122	StdExt1_6_Al_D3_R11	A.lumbricoides	30.956		
124	StdExt1_6_Al_D3_R12	A.lumbricoides	32.504		
126	StdExt1_6_Al_D3_R13	A.lumbricoides	33.273		
128	StdExt1_6_Al_D3_R14	A.lumbricoides	34.463		
130	StdExt1_6_Al_D3_R15	A.lumbricoides	32.057		
132	StdExt1_6_Al_D3_R16	A.lumbricoides	31.146		
134	StdExt1_6_Al_D3_R17	A.lumbricoides	32.633		
136	StdExt1_6_Al_D3_R18	A.lumbricoides	33.740		
138	StdExt1_6_Al_D3_R19	A.lumbricoides	32.245		
123	StdExt1_6_Al_D3_R2	A.lumbricoides	33.904		
140	StdExt1_6_Al_D3_R20	A.lumbricoides	32.299		
125	StdExt1_6_Al_D3_R3	A.lumbricoides	32.407		
127	StdExt1_6_Al_D3_R4	A.lumbricoides	31.218		
129	StdExt1_6_Al_D3_R5	A.lumbricoides	32.479		
131	StdExt1_6_Al_D3_R6	A.lumbricoides	31.007		
133	StdExt1_6_Al_D3_R7	A.lumbricoides	32.190		
135	StdExt1_6_Al_D3_R8	A.lumbricoides	30.842		
137	StdExt1_6_Al_D3_R9	A.lumbricoides	31.322		

Second Dilution (1:2)					
Well	Sample Name	Target Name	CT		
169	StdExt1_6_Al_D4_R1	*A.lumbricoides*	35.216	1.25E-04	4th Dilution
187	StdExt1_6_Al_D4_R10	*A.lumbricoides*	31.984		
170	StdExt1_6_Al_D4_R11	*A.lumbricoides*	34.237		
172	StdExt1_6_Al_D4_R12	*A.lumbricoides*	33.063		
174	StdExt1_6_Al_D4_R13	*A.lumbricoides*	32.529		
176	StdExt1_6_Al_D4_R14	*A.lumbricoides*	33.823		
178	StdExt1_6_Al_D4_R15	*A.lumbricoides*	33.165		
180	StdExt1_6_Al_D4_R16	*A.lumbricoides*	35.175		
182	StdExt1_6_Al_D4_R17	*A.lumbricoides*	31.635		
184	StdExt1_6_Al_D4_R18	*A.lumbricoides*	33.941		
186	StdExt1_6_Al_D4_R19	*A.lumbricoides*	32.639		
171	StdExt1_6_Al_D4_R2	*A.lumbricoides*	34.165		
188	StdExt1_6_Al_D4_R20	*A.lumbricoides*	33.353		
173	StdExt1_6_Al_D4_R3	*A.lumbricoides*	38.900		
175	StdExt1_6_Al_D4_R4	*A.lumbricoides*	34.765		
177	StdExt1_6_Al_D4_R5	*A.lumbricoides*	38.360		
179	StdExt1_6_Al_D4_R6	*A.lumbricoides*	36.389		
181	StdExt1_6_Al_D4_R7	*A.lumbricoides*	37.786		
183	StdExt1_6_Al_D4_R8	*A.lumbricoides*	33.883		
185	StdExt1_6_Al_D4_R9	*A.lumbricoides*	35.750		
25	StdExt1_6_Al_D5_R1	*A.lumbricoides*	Undetermined	6.25E-05	5th Dilution
26	StdExt1_6_Al_D5_R11	*A.lumbricoides*	37.122		
27	StdExt1_6_Al_D5_R2	*A.lumbricoides*	34.363		
28	StdExt1_6_Al_D5_R12	*A.lumbricoides*	35.149		
29	StdExt1_6_Al_D5_R3	*A.lumbricoides*	35.674		
30	StdExt1_6_Al_D5_R13	*A.lumbricoides*	34.477		
31	StdExt1_6_Al_D5_R4	*A.lumbricoides*	35.099		
32	StdExt1_6_Al_D5_R14	*A.lumbricoides*	34.094		
33	StdExt1_6_Al_D5_R5	*A.lumbricoides*	35.277		
34	StdExt1_6_Al_D5_R15	*A.lumbricoides*	35.843		
35	StdExt1_6_Al_D5_R6	*A.lumbricoides*	35.794		
36	StdExt1_6_Al_D5_R16	*A.lumbricoides*	37.658		
37	StdExt1_6_Al_D5_R7	*A.lumbricoides*	Undetermined		
38	StdExt1_6_Al_D5_R17	*A.lumbricoides*	34.468		
39	StdExt1_6_Al_D5_R8	*A.lumbricoides*	35.227		
40	StdExt1_6_Al_D5_R18	*A.lumbricoides*	Undetermined		
41	StdExt1_6_Al_D5_R9	*A.lumbricoides*	36.832		
42	StdExt1_6_Al_D5_R19	*A.lumbricoides*	34.332		
43	StdExt1_6_Al_D5_R10	*A.lumbricoides*	36.050		
44	StdExt1_6_Al_D5_R20	*A.lumbricoides*	35.009		
47	StdExt1_6_Al_D6_R1	*A.lumbricoides*	Undetermined	3.13E-05	6th Dilution
48	StdExt1_6_Al_D6_R11	*A.lumbricoides*	Undetermined		
73	StdExt1_6_Al_D6_R2	*A.lumbricoides*	Undetermined		
74	StdExt1_6_Al_D6_R12	*A.lumbricoides*	Undetermined		
75	StdExt1_6_Al_D6_R3	*A.lumbricoides*	Undetermined		
76	StdExt1_6_Al_D6_R13	*A.lumbricoides*	Undetermined		
77	StdExt1_6_Al_D6_R4	*A.lumbricoides*	Undetermined		
78	StdExt1_6_Al_D6_R14	*A.lumbricoides*	35.311		
79	StdExt1_6_Al_D6_R5	*A.lumbricoides*	Undetermined		
80	StdExt1_6_Al_D6_R15	*A.lumbricoides*	37.398		
81	StdExt1_6_Al_D6_R6	*A.lumbricoides*	21.451		
82	StdExt1_6_Al_D6_R16	*A.lumbricoides*	Undetermined		
83	StdExt1_6_Al_D6_R7	*A.lumbricoides*	Undetermined		
84	StdExt1_6_Al_D6_R17	*A.lumbricoides*	Undetermined		
85	StdExt1_6_Al_D6_R8	*A.lumbricoides*	Undetermined		
86	StdExt1_6_Al_D6_R18	*A.lumbricoides*	Undetermined		
87	StdExt1_6_Al_D6_R9	*A.lumbricoides*	Undetermined		
88	StdExt1_6_Al_D6_R19	*A.lumbricoides*	Undetermined		
89	StdExt1_6_Al_D6_R10	*A.lumbricoides*	35.910		
90	StdExt1_6_Al_D6_R20	*A.lumbricoides*	Undetermined		
91	StdExt1_6_Al_D7_R1	*A.lumbricoides*	Undetermined	1.56E-05	7th Dilution
92	StdExt1_6_Al_D7_R11	*A.lumbricoides*	Undetermined		
121	StdExt1_6_Al_D7_R2	*A.lumbricoides*	Undetermined		
122	StdExt1_6_Al_D7_R12	*A.lumbricoides*	Undetermined		
123	StdExt1_6_Al_D7_R3	*A.lumbricoides*	Undetermined		
124	StdExt1_6_Al_D7_R13	*A.lumbricoides*	Undetermined		
125	StdExt1_6_Al_D7_R4	*A.lumbricoides*	35.615		
126	StdExt1_6_Al_D7_R14	*A.lumbricoides*	Undetermined		
127	StdExt1_6_Al_D7_R5	*A.lumbricoides*	Undetermined		
128	StdExt1_6_Al_D7_R15	*A.lumbricoides*	Undetermined		
129	StdExt1_6_Al_D7_R6	*A.lumbricoides*	Undetermined		
130	StdExt1_6_Al_D7_R16	*A.lumbricoides*	Undetermined		
131	StdExt1_6_Al_D7_R7	*A.lumbricoides*	Undetermined		
132	StdExt1_6_Al_D7_R17	*A.lumbricoides*	Undetermined		
133	StdExt1_6_Al_D7_R8	*A.lumbricoides*	37.860		
134	StdExt1_6_Al_D7_R18	*A.lumbricoides*	Undetermined		
135	StdExt1_6_Al_D7_R9	*A.lumbricoides*	Undetermined		
136	StdExt1_6_Al_D7_R19	*A.lumbricoides*	38.819		
137	StdExt1_6_Al_D7_R10	*A.lumbricoides*	Undetermined		
138	StdExt1_6_Al_D7_R20	*A.lumbricoides*	32.877		

Table A6. 6: *1:10 Serial Dilution Ct Scores for A. duodenale. Note there are 10 replicates of each dilution point.*

First Dilution (1:10)					
Well	Sample Name	Target Name	CT		
11	StdExt1_208_Ad_0.1_1	A. duodenale	32.320		
20	StdExt1_208_Ad_0.1_10	A. duodenale	32.429		
12	StdExt1_208_Ad_0.1_2	A. duodenale	31.835		
13	StdExt1_208_Ad_0.1_3	A. duodenale	32.014		
14	StdExt1_208_Ad_0.1_4	A. duodenale	31.738		
15	StdExt1_208_Ad_0.1_5	A. duodenale	31.590		
16	StdExt1_208_Ad_0.1_6	A. duodenale	31.858		
17	StdExt1_208_Ad_0.1_7	A. duodenale	31.996		
18	StdExt1_208_Ad_0.1_8	A. duodenale	32.508		
19	StdExt1_208_Ad_0.1_9	A. duodenale	31.875	1.00E-01	1st Dilution
83	StdExt1_208_Ad_0.01_1	A. duodenale	35.675		
92	StdExt1_208_Ad_0.01_10	A. duodenale	35.728		
84	StdExt1_208_Ad_0.01_2	A. duodenale	34.523		
85	StdExt1_208_Ad_0.01_3	A. duodenale	36.200		
86	StdExt1_208_Ad_0.01_4	A. duodenale	37.116		
87	StdExt1_208_Ad_0.01_5	A. duodenale	37.102		
88	StdExt1_208_Ad_0.01_6	A. duodenale	34.971		
89	StdExt1_208_Ad_0.01_7	A. duodenale	35.025		
90	StdExt1_208_Ad_0.01_8	A. duodenale	36.862		
91	StdExt1_208_Ad_0.01_9	A. duodenale	Undetermined	1.00E-02	2nd Dilution
131	StdExt1_208_Ad_0.001_1	A. duodenale	39.028		
140	StdExt1_208_Ad_0.001_10	A. duodenale	Undetermined		
132	StdExt1_208_Ad_0.001_2	A. duodenale	Undetermined		
133	StdExt1_208_Ad_0.001_3	A. duodenale	Undetermined		
134	StdExt1_208_Ad_0.001_4	A. duodenale	Undetermined		
135	StdExt1_208_Ad_0.001_5	A. duodenale	Undetermined		
136	StdExt1_208_Ad_0.001_6	A. duodenale	Undetermined		
137	StdExt1_208_Ad_0.001_7	A. duodenale	Undetermined		
138	StdExt1_208_Ad_0.001_8	A. duodenale	Undetermined		
139	StdExt1_208_Ad_0.001_9	A. duodenale	Undetermined	1.00E-03	3rd Dilution
179	StdExt1_208_Ad_0.0001_1	A. duodenale	Undetermined		
188	StdExt1_208_Ad_0.0001_10	A. duodenale	Undetermined		
180	StdExt1_208_Ad_0.0001_2	A. duodenale	Undetermined		
181	StdExt1_208_Ad_0.0001_3	A. duodenale	Undetermined		
182	StdExt1_208_Ad_0.0001_4	A. duodenale	Undetermined		
183	StdExt1_208_Ad_0.0001_5	A. duodenale	Undetermined		
184	StdExt1_208_Ad_0.0001_6	A. duodenale	Undetermined		
185	StdExt1_208_Ad_0.0001_7	A. duodenale	Undetermined		
186	StdExt1_208_Ad_0.0001_8	A. duodenale	Undetermined		
187	StdExt1_208_Ad_0.0001_9	A. duodenale	Undetermined	1.00E-04	4th Dilution
227	StdExt1_208_Ad_0.00001_1	A. duodenale	Undetermined		
236	StdExt1_208_Ad_0.00001_10	A. duodenale	Undetermined		
228	StdExt1_208_Ad_0.00001_2	A. duodenale	Undetermined		
229	StdExt1_208_Ad_0.00001_3	A. duodenale	Undetermined		
230	StdExt1_208_Ad_0.00001_4	A. duodenale	Undetermined		
231	StdExt1_208_Ad_0.00001_5	A. duodenale	Undetermined		
232	StdExt1_208_Ad_0.00001_6	A. duodenale	Undetermined		
233	StdExt1_208_Ad_0.00001_7	A. duodenale	Undetermined		
234	StdExt1_208_Ad_0.00001_8	A. duodenale	Undetermined		
235	StdExt1_208_Ad_0.00001_9	A. duodenale	Undetermined	1.00E-05	5th Dilution
275	StdExt1_208_Ad_0.000001_1	A. duodenale	Undetermined		
284	StdExt1_208_Ad_0.000001_10	A. duodenale	Undetermined		
276	StdExt1_208_Ad_0.000001_2	A. duodenale	Undetermined		
277	StdExt1_208_Ad_0.000001_3	A. duodenale	Undetermined		
278	StdExt1_208_Ad_0.000001_4	A. duodenale	Undetermined		
279	StdExt1_208_Ad_0.000001_5	A. duodenale	Undetermined		
280	StdExt1_208_Ad_0.000001_6	A. duodenale	Undetermined		
281	StdExt1_208_Ad_0.000001_7	A. duodenale	Undetermined		
282	StdExt1_208_Ad_0.000001_8	A. duodenale	Undetermined		
283	StdExt1_208_Ad_0.000001_9	A. duodenale	Undetermined	1.00E-06	6th Dilution

Table A6. 7: *1:2 Serial Dilution Ct Scores for A. duodenale. Note there are 20 replicates of each dilution point.*

Second Dilution (1:2)					
Well	Sample Name	Target Name	CT		
47	NTC	A. duodenale	Undetermined		
48	Pos. PCR	A. duodenale	27.999		
217	StdExt1_208_Ad_D1_R1	A. duodenale	30.835	1.00E-01	1st Dilution
235	StdExt1_208_Ad_D1_R10	A. duodenale	31.735		
218	StdExt1_208_Ad_D1_R11	A. duodenale	31.079		
220	StdExt1_208_Ad_D1_R12	A. duodenale	30.665		
222	StdExt1_208_Ad_D1_R13	A. duodenale	31.353		
224	StdExt1_208_Ad_D1_R14	A. duodenale	30.747		
226	StdExt1_208_Ad_D1_R15	A. duodenale	31.399		
228	StdExt1_208_Ad_D1_R16	A. duodenale	31.000		
230	StdExt1_208_Ad_D1_R17	A. duodenale	30.721		
232	StdExt1_208_Ad_D1_R18	A. duodenale	30.628		
234	StdExt1_208_Ad_D1_R19	A. duodenale	31.446		
219	StdExt1_208_Ad_D1_R2	A. duodenale	30.857		
236	StdExt1_208_Ad_D1_R20	A. duodenale	31.319		
221	StdExt1_208_Ad_D1_R3	A. duodenale	30.966		
223	StdExt1_208_Ad_D1_R4	A. duodenale	30.512		
225	StdExt1_208_Ad_D1_R5	A. duodenale	31.231		
227	StdExt1_208_Ad_D1_R6	A. duodenale	31.110		
229	StdExt1_208_Ad_D1_R7	A. duodenale	31.095		
231	StdExt1_208_Ad_D1_R8	A. duodenale	30.912		
233	StdExt1_208_Ad_D1_R9	A. duodenale	31.270		
265	StdExt1_208_Ad_D2_R1	A. duodenale	31.749	5.00E-02	2nd Dilution
283	StdExt1_208_Ad_D2_R10	A. duodenale	31.977		
266	StdExt1_208_Ad_D2_R11	A. duodenale	31.307		
268	StdExt1_208_Ad_D2_R12	A. duodenale	31.647		
270	StdExt1_208_Ad_D2_R13	A. duodenale	31.925		
272	StdExt1_208_Ad_D2_R14	A. duodenale	31.940		
274	StdExt1_208_Ad_D2_R15	A. duodenale	32.198		
276	StdExt1_208_Ad_D2_R16	A. duodenale	33.388		
278	StdExt1_208_Ad_D2_R17	A. duodenale	31.587		
280	StdExt1_208_Ad_D2_R18	A. duodenale	33.051		
282	StdExt1_208_Ad_D2_R19	A. duodenale	31.971		
267	StdExt1_208_Ad_D2_R2	A. duodenale	32.638		
284	StdExt1_208_Ad_D2_R20	A. duodenale	31.540		
269	StdExt1_208_Ad_D2_R3	A. duodenale	32.289		
271	StdExt1_208_Ad_D2_R4	A. duodenale	31.475		
273	StdExt1_208_Ad_D2_R5	A. duodenale	32.493		
275	StdExt1_208_Ad_D2_R6	A. duodenale	32.496		
277	StdExt1_208_Ad_D2_R7	A. duodenale	31.644		
279	StdExt1_208_Ad_D2_R8	A. duodenale	31.664		
281	StdExt1_208_Ad_D2_R9	A. duodenale	32.665		
313	StdExt1_208_Ad_D3_R1	A. duodenale	33.788	2.50E-02	3rd Dilution
331	StdExt1_208_Ad_D3_R10	A. duodenale	34.538		
314	StdExt1_208_Ad_D3_R11	A. duodenale	32.405		
316	StdExt1_208_Ad_D3_R12	A. duodenale	32.440		
318	StdExt1_208_Ad_D3_R13	A. duodenale	32.586		
320	StdExt1_208_Ad_D3_R14	A. duodenale	33.110		
322	StdExt1_208_Ad_D3_R15	A. duodenale	33.641		
324	StdExt1_208_Ad_D3_R16	A. duodenale	32.904		
326	StdExt1_208_Ad_D3_R17	A. duodenale	34.067		
328	StdExt1_208_Ad_D3_R18	A. duodenale	34.057		
330	StdExt1_208_Ad_D3_R19	A. duodenale	32.790		
315	StdExt1_208_Ad_D3_R2	A. duodenale	34.918		
332	StdExt1_208_Ad_D3_R20	A. duodenale	33.329		
317	StdExt1_208_Ad_D3_R3	A. duodenale	32.474		
319	StdExt1_208_Ad_D3_R4	A. duodenale	32.488		
321	StdExt1_208_Ad_D3_R5	A. duodenale	33.430		
323	StdExt1_208_Ad_D3_R6	A. duodenale	32.920		
325	StdExt1_208_Ad_D3_R7	A. duodenale	32.355		
327	StdExt1_208_Ad_D3_R8	A. duodenale	32.292		
329	StdExt1_208_Ad_D3_R9	A. duodenale	32.236		

Second Dilution (1:2)					
Well	Sample Name	Target Name	CT		
361	StdExt1_208_Ad_D4_R1	A. duodenale	33.459	1.25E-02	4th Dilution
379	StdExt1_208_Ad_D4_R10	A. duodenale	33.383		
362	StdExt1_208_Ad_D4_R11	A. duodenale	34.610		
364	StdExt1_208_Ad_D4_R12	A. duodenale	36.116		
366	StdExt1_208_Ad_D4_R13	A. duodenale	34.729		
368	StdExt1_208_Ad_D4_R14	A. duodenale	33.443		
370	StdExt1_208_Ad_D4_R15	A. duodenale	34.368		
372	StdExt1_208_Ad_D4_R16	A. duodenale	35.491		
374	StdExt1_208_Ad_D4_R17	A. duodenale	33.878		
376	StdExt1_208_Ad_D4_R18	A. duodenale	34.646		
378	StdExt1_208_Ad_D4_R19	A. duodenale	34.343		
363	StdExt1_208_Ad_D4_R2	A. duodenale	34.167		
380	StdExt1_208_Ad_D4_R20	A. duodenale	34.457		
365	StdExt1_208_Ad_D4_R3	A. duodenale	34.042		
367	StdExt1_208_Ad_D4_R4	A. duodenale	34.761		
369	StdExt1_208_Ad_D4_R5	A. duodenale	33.673		
371	StdExt1_208_Ad_D4_R6	A. duodenale	33.608		
373	StdExt1_208_Ad_D4_R7	A. duodenale	33.122		
375	StdExt1_208_Ad_D4_R8	A. duodenale	34.628		
377	StdExt1_208_Ad_D4_R9	A. duodenale	34.139		
217	StdExt1_208_Ad_D5_R1	A. duodenale	Undetermined	6.25E-03	5th Dilution
218	StdExt1_208_Ad_D5_R11	A. duodenale	Undetermined		
219	StdExt1_208_Ad_D5_R2	A. duodenale	37.405		
220	StdExt1_208_Ad_D5_R12	A. duodenale	Undetermined		
221	StdExt1_208_Ad_D5_R3	A. duodenale	36.288		
222	StdExt1_208_Ad_D5_R13	A. duodenale	Undetermined		
223	StdExt1_208_Ad_D5_R4	A. duodenale	35.569		
224	StdExt1_208_Ad_D5_R14	A. duodenale	35.962		
225	StdExt1_208_Ad_D5_R5	A. duodenale	34.733		
226	StdExt1_208_Ad_D5_R15	A. duodenale	35.696		
227	StdExt1_208_Ad_D5_R6	A. duodenale	35.196		
228	StdExt1_208_Ad_D5_R16	A. duodenale	36.114		
229	StdExt1_208_Ad_D5_R7	A. duodenale	35.788		
230	StdExt1_208_Ad_D5_R17	A. duodenale	36.334		
231	StdExt1_208_Ad_D5_R8	A. duodenale	39.662		
232	StdExt1_208_Ad_D5_R18	A. duodenale	36.404		
233	StdExt1_208_Ad_D5_R9	A. duodenale	Undetermined		
234	StdExt1_208_Ad_D5_R19	A. duodenale	39.296		
235	StdExt1_208_Ad_D5_R10	A. duodenale	36.454		
236	StdExt1_208_Ad_D5_R20	A. duodenale	34.920		
265	StdExt1_208_Ad_D6_R1	A. duodenale	35.141	3.13E-03	6th Dilution
266	StdExt1_208_Ad_D6_R11	A. duodenale	Undetermined		
267	StdExt1_208_Ad_D6_R2	A. duodenale	36.431		
268	StdExt1_208_Ad_D6_R12	A. duodenale	Undetermined		
269	StdExt1_208_Ad_D6_R3	A. duodenale	Undetermined		
270	StdExt1_208_Ad_D6_R13	A. duodenale	35.366		
271	StdExt1_208_Ad_D6_R4	A. duodenale	38.609		
272	StdExt1_208_Ad_D6_R14	A. duodenale	Undetermined		
273	StdExt1_208_Ad_D6_R5	A. duodenale	Undetermined		
274	StdExt1_208_Ad_D6_R15	A. duodenale	35.641		
275	StdExt1_208_Ad_D6_R6	A. duodenale	38.139		
276	StdExt1_208_Ad_D6_R16	A. duodenale	34.706		
277	StdExt1_208_Ad_D6_R7	A. duodenale	Undetermined		
278	StdExt1_208_Ad_D6_R17	A. duodenale	39.343		
279	StdExt1_208_Ad_D6_R8	A. duodenale	38.058		
280	StdExt1_208_Ad_D6_R18	A. duodenale	Undetermined		
281	StdExt1_208_Ad_D6_R9	A. duodenale	36.533		
282	StdExt1_208_Ad_D6_R19	A. duodenale	Undetermined		
283	StdExt1_208_Ad_D6_R10	A. duodenale	Undetermined		
284	StdExt1_208_Ad_D6_R20	A. duodenale	36.423		
313	StdExt1_208_Ad_D7_R1	A. duodenale	39.310	1.56E-03	7th Dilution
314	StdExt1_208_Ad_D7_R11	A. duodenale	38.522		
315	StdExt1_208_Ad_D7_R2	A. duodenale	Undetermined		
316	StdExt1_208_Ad_D7_R12	A. duodenale	Undetermined		
317	StdExt1_208_Ad_D7_R3	A. duodenale	36.146		
318	StdExt1_208_Ad_D7_R13	A. duodenale	Undetermined		
319	StdExt1_208_Ad_D7_R4	A. duodenale	36.495		
320	StdExt1_208_Ad_D7_R14	A. duodenale	Undetermined		
321	StdExt1_208_Ad_D7_R5	A. duodenale	35.162		
322	StdExt1_208_Ad_D7_R15	A. duodenale	Undetermined		
323	StdExt1_208_Ad_D7_R6	A. duodenale	Undetermined		
324	StdExt1_208_Ad_D7_R16	A. duodenale	Undetermined		
325	StdExt1_208_Ad_D7_R7	A. duodenale	37.771		
326	StdExt1_208_Ad_D7_R17	A. duodenale	Undetermined		
327	StdExt1_208_Ad_D7_R8	A. duodenale	Undetermined		
328	StdExt1_208_Ad_D7_R18	A. duodenale	Undetermined		
329	StdExt1_208_Ad_D7_R9	A. duodenale	36.275		
330	StdExt1_208_Ad_D7_R19	A. duodenale	38.044		
331	StdExt1_208_Ad_D7_R10	A. duodenale	Undetermined		
332	StdExt1_208_Ad_D7_R20	A. duodenale	39.620		

Table A6. 8: *1:10 Serial Dilution Ct Scores for T. trichiura. Note there are 10 replicates of each dilution point.*

First Dilution (1:10)					
Well	Sample Name	Target Name	CT		
A1	StdExt2_44_Tt_D1_R1	T.trichiura	26.349		
A2	StdExt2_44_Tt_D1_R2	T.trichiura	26.527		
A3	StdExt2_44_Tt_D1_R3	T.trichiura	26.340		
A4	StdExt2_44_Tt_D1_R4	T.trichiura	26.325		
A5	StdExt2_44_Tt_D1_R5	T.trichiura	26.568		
A6	StdExt2_44_Tt_D1_R6	T.trichiura	26.479		
A7	StdExt2_44_Tt_D1_R7	T.trichiura	26.597		
A8	StdExt2_44_Tt_D1_R8	T.trichiura	26.482		
A9	StdExt2_44_Tt_D1_R9	T.trichiura	26.556		
A10	StdExt2_44_Tt_D1_R10	T.trichiura	26.629	1.00E-01	1st Dilution
C1	StdExt2_44_Tt_D2_R1	T.trichiura	29.823		
C2	StdExt2_44_Tt_D2_R2	T.trichiura	29.700		
C3	StdExt2_44_Tt_D2_R3	T.trichiura	29.641		
C4	StdExt2_44_Tt_D2_R4	T.trichiura	29.797		
C5	StdExt2_44_Tt_D2_R5	T.trichiura	29.688		
C6	StdExt2_44_Tt_D2_R6	T.trichiura	29.944		
C7	StdExt2_44_Tt_D2_R7	T.trichiura	29.749		
C8	StdExt2_44_Tt_D2_R8	T.trichiura	29.873		
C9	StdExt2_44_Tt_D2_R9	T.trichiura	29.981		
C10	StdExt2_44_Tt_D2_R10	T.trichiura	30.079	1.00E-02	2nd Dilution
E1	StdExt2_44_Tt_D3_R1	T.trichiura	33.091		
E2	StdExt2_44_Tt_D3_R2	T.trichiura	32.781		
E3	StdExt2_44_Tt_D3_R3	T.trichiura	33.021		
E4	StdExt2_44_Tt_D3_R4	T.trichiura	33.369		
E5	StdExt2_44_Tt_D3_R5	T.trichiura	32.837		
E6	StdExt2_44_Tt_D3_R6	T.trichiura	33.038		
E7	StdExt2_44_Tt_D3_R7	T.trichiura	32.483		
E8	StdExt2_44_Tt_D3_R8	T.trichiura	33.102		
E9	StdExt2_44_Tt_D3_R9	T.trichiura	32.490		
E10	StdExt2_44_Tt_D3_R10	T.trichiura	33.286	1.00E-03	3rd Dilution
G1	StdExt2_44_Tt_D4_R1	T.trichiura	36.585		
G2	StdExt2_44_Tt_D4_R2	T.trichiura	35.552		
G3	StdExt2_44_Tt_D4_R3	T.trichiura	36.66449		
G4	StdExt2_44_Tt_D4_R4	T.trichiura	38.441		
G5	StdExt2_44_Tt_D4_R5	T.trichiura	37.228886		
G6	StdExt2_44_Tt_D4_R6	T.trichiura	Undetermined		
G7	StdExt2_44_Tt_D4_R7	T.trichiura	35.310		
G8	StdExt2_44_Tt_D4_R8	T.trichiura	37.101		
G9	StdExt2_44_Tt_D4_R9	T.trichiura	37.229		
G10	StdExt2_44_Tt_D4_R10	T.trichiura	37.217	1.00E-04	4th Dilution
I1	StdExt2_44_Tt_D5_R1	T.trichiura	Undetermined		
I2	StdExt2_44_Tt_D5_R2	T.trichiura	Undetermined		
I3	StdExt2_44_Tt_D5_R3	T.trichiura	Undetermined		
I4	StdExt2_44_Tt_D5_R4	T.trichiura	Undetermined		
I5	StdExt2_44_Tt_D5_R5	T.trichiura	Undetermined		
I6	StdExt2_44_Tt_D5_R6	T.trichiura	Undetermined		
I7	StdExt2_44_Tt_D5_R7	T.trichiura	Undetermined		
I8	StdExt2_44_Tt_D5_R8	T.trichiura	Undetermined		
I9	StdExt2_44_Tt_D5_R9	T.trichiura	Undetermined		
I10	StdExt2_44_Tt_D5_R10	T.trichiura	Undetermined	1.00E-05	5th Dilution

Table A6. 9: *1:2 Serial Dilution Ct Scores for T. trichiura. Note there are 20 replicates of each dilution point*

Second Dilution (1:2)					
Well	Sample Name	Target Name	CT		
A23	NTC	T.trichiura	Undetermined		
A24	Pos. PCR	T.trichiura	28.669		
A1	StdExt2_44_Tt_D1_R1	T.trichiura	31.790	1.00E-03	1st Dilution
A2	StdExt2_44_Tt_D1_R2	T.trichiura	32.764		
A3	StdExt2_44_Tt_D1_R3	T.trichiura	32.411		
A4	StdExt2_44_Tt_D1_R4	T.trichiura	32.039		
A5	StdExt2_44_Tt_D1_R5	T.trichiura	32.055		
A6	StdExt2_44_Tt_D1_R6	T.trichiura	32.404		
A7	StdExt2_44_Tt_D1_R7	T.trichiura	32.443		
A8	StdExt2_44_Tt_D1_R8	T.trichiura	32.753		
A9	StdExt2_44_Tt_D1_R9	T.trichiura	32.029		
A10	StdExt2_44_Tt_D1_R10	T.trichiura	32.185		
A11	StdExt2_44_Tt_D1_R11	T.trichiura	33.364		
A12	StdExt2_44_Tt_D1_R12	T.trichiura	32.505		
A13	StdExt2_44_Tt_D1_R13	T.trichiura	32.119		
A14	StdExt2_44_Tt_D1_R14	T.trichiura	31.844		
A15	StdExt2_44_Tt_D1_R15	T.trichiura	32.863		
A16	StdExt2_44_Tt_D1_R16	T.trichiura	32.717		
A17	StdExt2_44_Tt_D1_R17	T.trichiura	32.586		
A18	StdExt2_44_Tt_D1_R18	T.trichiura	32.012		
A19	StdExt2_44_Tt_D1_R19	T.trichiura	32.257		
A20	StdExt2_44_Tt_D1_R20	T.trichiura	32.331		
C1	StdExt2_44_Tt_D2_R1	T.trichiura	33.484	5.00E-04	2nd Dilution
C2	StdExt2_44_Tt_D2_R2	T.trichiura	34.339		
C3	StdExt2_44_Tt_D2_R3	T.trichiura	32.372		
C4	StdExt2_44_Tt_D2_R4	T.trichiura	Well Failure		
C5	StdExt2_44_Tt_D2_R5	T.trichiura	33.952		
C6	StdExt2_44_Tt_D2_R6	T.trichiura	33.536		
C7	StdExt2_44_Tt_D2_R7	T.trichiura	33.064		
C8	StdExt2_44_Tt_D2_R8	T.trichiura	35.002		
C9	StdExt2_44_Tt_D2_R9	T.trichiura	33.176		
C10	StdExt2_44_Tt_D2_R10	T.trichiura	34.306		
C11	StdExt2_44_Tt_D2_R11	T.trichiura	33.744		
C12	StdExt2_44_Tt_D2_R12	T.trichiura	35.296		
C13	StdExt2_44_Tt_D2_R13	T.trichiura	33.319		
C14	StdExt2_44_Tt_D2_R14	T.trichiura	33.081		
C15	StdExt2_44_Tt_D2_R15	T.trichiura	33.607		
C16	StdExt2_44_Tt_D2_R16	T.trichiura	34.105		
C17	StdExt2_44_Tt_D2_R17	T.trichiura	33.260		
C18	StdExt2_44_Tt_D2_R18	T.trichiura	35.116		
C19	StdExt2_44_Tt_D2_R19	T.trichiura	33.547		
C20	StdExt2_44_Tt_D2_R20	T.trichiura	33.956		
E1	StdExt2_44_Tt_D3_R1	T.trichiura	34.817	2.50E-04	3rd Dilution
E2	StdExt2_44_Tt_D3_R2	T.trichiura	35.495		
E3	StdExt2_44_Tt_D3_R3	T.trichiura	34.640		
E4	StdExt2_44_Tt_D3_R4	T.trichiura	34.201		
E5	StdExt2_44_Tt_D3_R5	T.trichiura	34.845		
E6	StdExt2_44_Tt_D3_R6	T.trichiura	35.112		
E7	StdExt2_44_Tt_D3_R7	T.trichiura	34.524		
E8	StdExt2_44_Tt_D3_R8	T.trichiura	33.390		
E9	StdExt2_44_Tt_D3_R9	T.trichiura	33.927		
E10	StdExt2_44_Tt_D3_R10	T.trichiura	34.316		
E11	StdExt2_44_Tt_D3_R11	T.trichiura	34.227		
E12	StdExt2_44_Tt_D3_R12	T.trichiura	34.327		
E13	StdExt2_44_Tt_D3_R13	T.trichiura	32.972		
E14	StdExt2_44_Tt_D3_R14	T.trichiura	34.413		
E15	StdExt2_44_Tt_D3_R15	T.trichiura	33.647		
E16	StdExt2_44_Tt_D3_R16	T.trichiura	34.444		
E17	StdExt2_44_Tt_D3_R17	T.trichiura	33.599		
E18	StdExt2_44_Tt_D3_R18	T.trichiura	35.071		
E19	StdExt2_44_Tt_D3_R19	T.trichiura	34.542		
E20	StdExt2_44_Tt_D3_R20	T.trichiura	34.128		

Second Dilution (1:2)					
Well	Sample Name	Target Name	CT		
G1	StdExt2_44_Tt_D4_R1	T.trichiura	34.668	1.25E-04	4th Dilution
G2	StdExt2_44_Tt_D4_R2	T.trichiura	37.239		
G3	StdExt2_44_Tt_D4_R3	T.trichiura	35.905		
G4	StdExt2_44_Tt_D4_R4	T.trichiura	34.152		
G5	StdExt2_44_Tt_D4_R5	T.trichiura	36.237		
G6	StdExt2_44_Tt_D4_R6	T.trichiura	35.862		
G7	StdExt2_44_Tt_D4_R7	T.trichiura	35.479		
G8	StdExt2_44_Tt_D4_R8	T.trichiura	Undetermined		
G9	StdExt2_44_Tt_D4_R9	T.trichiura	38.417		
G10	StdExt2_44_Tt_D4_R10	T.trichiura	34.398		
G11	StdExt2_44_Tt_D4_R11	T.trichiura	37.293		
G12	StdExt2_44_Tt_D4_R12	T.trichiura	35.716		
G13	StdExt2_44_Tt_D4_R13	T.trichiura	35.967		
G14	StdExt2_44_Tt_D4_R14	T.trichiura	37.410		
G15	StdExt2_44_Tt_D4_R15	T.trichiura	36.004		
G16	StdExt2_44_Tt_D4_R16	T.trichiura	37.509		
G17	StdExt2_44_Tt_D4_R17	T.trichiura	35.416		
G18	StdExt2_44_Tt_D4_R18	T.trichiura	37.199		
G19	StdExt2_44_Tt_D4_R19	T.trichiura	36.049		
G20	StdExt2_44_Tt_D4_R20	T.trichiura	36.510		
I1	StdExt2_44_Tt_D5_R1	T.trichiura	Undetermined	6.25E-05	5th Dilution
I2	StdExt2_44_Tt_D5_R2	T.trichiura	35.935963		
I3	StdExt2_44_Tt_D5_R3	T.trichiura	Undetermined		
I4	StdExt2_44_Tt_D5_R4	T.trichiura	36.615772		
I5	StdExt2_44_Tt_D5_R5	T.trichiura	37.701		
I6	StdExt2_44_Tt_D5_R6	T.trichiura	34.70892		
I7	StdExt2_44_Tt_D5_R7	T.trichiura	36.367		
I8	StdExt2_44_Tt_D5_R8	T.trichiura	36.382		
I9	StdExt2_44_Tt_D5_R9	T.trichiura	37.301		
I10	StdExt2_44_Tt_D5_R10	T.trichiura	Undetermined		
I11	StdExt2_44_Tt_D5_R11	T.trichiura	37.311		
I12	StdExt2_44_Tt_D5_R12	T.trichiura	35.766		
I13	StdExt2_44_Tt_D5_R13	T.trichiura	35.665		
I14	StdExt2_44_Tt_D5_R14	T.trichiura	34.397		
I15	StdExt2_44_Tt_D5_R15	T.trichiura	37.209		
I16	StdExt2_44_Tt_D5_R16	T.trichiura	35.798		
I17	StdExt2_44_Tt_D5_R17	T.trichiura	37.20936		
I18	StdExt2_44_Tt_D5_R18	T.trichiura	Undetermined		
I19	StdExt2_44_Tt_D5_R19	T.trichiura	34.081		
I20	StdExt2_44_Tt_D5_R20	T.trichiura	36.526		
K1	StdExt2_44_Tt_D6_R1	T.trichiura	35.507	3.13E-05	6th Dilution
K2	StdExt2_44_Tt_D6_R2	T.trichiura	Undetermined		
K3	StdExt2_44_Tt_D6_R3	T.trichiura	Undetermined		
K4	StdExt2_44_Tt_D6_R4	T.trichiura	34.44711		
K5	StdExt2_44_Tt_D6_R5	T.trichiura	37.265686		
K6	StdExt2_44_Tt_D6_R6	T.trichiura	36.959		
K7	StdExt2_44_Tt_D6_R7	T.trichiura	Undetermined		
K8	StdExt2_44_Tt_D6_R8	T.trichiura	36.441128		
K9	StdExt2_44_Tt_D6_R9	T.trichiura	Undetermined		
K10	StdExt2_44_Tt_D6_R10	T.trichiura	37.383		
K11	StdExt2_44_Tt_D6_R11	T.trichiura	37.075		
K12	StdExt2_44_Tt_D6_R12	T.trichiura	Undetermined		
K13	StdExt2_44_Tt_D6_R13	T.trichiura	37.114407		
K14	StdExt2_44_Tt_D6_R14	T.trichiura	Undetermined		
K15	StdExt2_44_Tt_D6_R15	T.trichiura	Undetermined		
K16	StdExt2_44_Tt_D6_R16	T.trichiura	Undetermined		
K17	StdExt2_44_Tt_D6_R17	T.trichiura	Undetermined		
K18	StdExt2_44_Tt_D6_R18	T.trichiura	37.123684		
K19	StdExt2_44_Tt_D6_R19	T.trichiura	37.234425		
K20	StdExt2_44_Tt_D6_R20	T.trichiura	Undetermined		
M1	StdExt2_44_Tt_D7_R1	T.trichiura	Undetermined	1.56E-05	7th Dilution
M2	StdExt2_44_Tt_D7_R2	T.trichiura	36.758		
M3	StdExt2_44_Tt_D7_R3	T.trichiura	Undetermined		
M4	StdExt2_44_Tt_D7_R4	T.trichiura	37.67536		
M5	StdExt2_44_Tt_D7_R5	T.trichiura	37.371		
M6	StdExt2_44_Tt_D7_R6	T.trichiura	Undetermined		
M7	StdExt2_44_Tt_D7_R7	T.trichiura	36.840		
M8	StdExt2_44_Tt_D7_R8	T.trichiura	Undetermined		
M9	StdExt2_44_Tt_D7_R9	T.trichiura	Undetermined		
M10	StdExt2_44_Tt_D7_R10	T.trichiura	39.263245		
M11	StdExt2_44_Tt_D7_R11	T.trichiura	Undetermined		
M12	StdExt2_44_Tt_D7_R12	T.trichiura	Undetermined		
M13	StdExt2_44_Tt_D7_R13	T.trichiura	Undetermined		
M14	StdExt2_44_Tt_D7_R14	T.trichiura	Undetermined		
M15	StdExt2_44_Tt_D7_R15	T.trichiura	Undetermined		
M16	StdExt2_44_Tt_D7_R16	T.trichiura	Undetermined		
M17	StdExt2_44_Tt_D7_R17	T.trichiura	Undetermined		
M18	StdExt2_44_Tt_D7_R18	T.trichiura	Undetermined		
M19	StdExt2_44_Tt_D7_R19	T.trichiura	35.19326		
M20	StdExt2_44_Tt_D7_R20	T.trichiura	38.251		

Appendix A7: 200 Sample Experiment Data

Figure A7.1: *The 200 sample experiment found 48 positive samples across 4 extractions. Within these samples, there was discordance, both between sites and within sites.*

		Quantigen		Smith			
	**Species**	**Extract 1**	**Extract 2**	**Extract 3**	**Extract 4**	**Quantigen**	**Smith**
1	*N. americanus*	25.3	24.2	18.13	18.41	Positive	Positive
	T.trichiura	Undetermined	Undetermined	Undetermined	Undetermined		
	A. lumbricoides	Undetermined	Undetermined	Undetermined	Undetermined		
2	*N. americanus*	36.3	35.9	32.4	26.7	Positive	Positive
	T.trichiura	Undetermined	Undetermined	Undetermined	Undetermined		
	A. lumbricoides	Undetermined	Undetermined	Undetermined	Undetermined		
3	*N. americanus*	23.6	23.5	21.11	17.29	Positive	Positive
	T.trichiura	Undetermined	Undetermined	Undetermined	Undetermined		
	A. lumbricoides	Undetermined	Undetermined	Undetermined	Undetermined		
4	*N. americanus*	28.4	28.6	23.91	32.14	Positive	Positive
	T.trichiura	Undetermined	Undetermined	Undetermined	Undetermined		
	A. lumbricoides	Undetermined	Undetermined	Undetermined	Undetermined		
5	*N. americanus*	28.3	27.2	23.68	24.36	Positive	Positive
	T.trichiura	Undetermined	Undetermined	Undetermined	Undetermined		
	A. lumbricoides	Undetermined	Undetermined	Undetermined	Undetermined		
6	*N. americanus*	26.9	28.5	20.77	22.83	Positive	Positive
	T.trichiura	Undetermined	Undetermined	Undetermined	Undetermined		
	A. lumbricoides	Undetermined	Undetermined	Undetermined	Undetermined		
7	*N. americanus*	36.7	36.7	36.2	29.1	Positive	Positive
	T.trichiura	Undetermined	Undetermined	Undetermined	Undetermined		
	A. lumbricoides	Undetermined	Undetermined	Undetermined	Undetermined		
8	*N. americanus*	33.3	34.1	30.46	29.98	Positive	Positive
	T.trichiura	Undetermined	Undetermined	Undetermined	Undetermined		
	A. lumbricoides	Undetermined	Undetermined	Undetermined	Undetermined		
9	*N. americanus*	Undetermined	Undetermined	Undetermined	Undetermined	Positive	Positive
	T.trichiura	Undetermined	Undetermined	Undetermined	Undetermined		
	A. lumbricoides	19.8	20.8	15.6	15.2		
10	*N. americanus*	24.7	22.9	17.30	20.57	Positive	Positive
	T.trichiura	Undetermined	Undetermined	Undetermined	Undetermined		
	A. lumbricoides	Undetermined	Undetermined	Undetermined	Undetermined		

**11**	***N. americanus***	**Undetermined**	**Undetermined**	**Undetermined**	**Undetermined**	**Positive**	**Positive**
	T.trichiura	**Undetermined**	**Undetermined**	**Undetermined**	**Undetermined**		
	A. lumbricoides	**18.5**	**19.1**	**14.4**	**14.9**		
**12**	***N. americanus***	**Undetermined**	**Undetermined**	**Undetermined**	**Undetermined**	**Positive**	**Positive**
	T.trichiura	**Undetermined**	**Undetermined**	**Undetermined**	**Undetermined**		
	A. lumbricoides	**34.7**	**37.0**	**32.6**	**32.1**		
**13**	***N. americanus***	**25.1**	**31.0**	**19.69**	**18.99**	**Positive**	**Positive**
	T.trichiura	**Undetermined**	**Undetermined**	**Undetermined**	**Undetermined**		
	A. lumbricoides	**Undetermined**	**Undetermined**	**Undetermined**	**Undetermined**		
**14**	***N. americanus***	**Undetermined**	**Undetermined**	**Undetermined**	**Undetermined**	**Positive**	**Positive**
	T.trichiura	**Undetermined**	**Undetermined**	**Undetermined**	**Undetermined**		
	A. lumbricoides	**11.7**	**12.3**	**7.8**	**9.3**		
**15**	***N. americanus***	**Undetermined**	**Undetermined**	**Undetermined**	**Undetermined**	**Positive**	**Positive**
	T.trichiura	**Undetermined**	**Undetermined**	**Undetermined**	**Undetermined**		
	A. lumbricoides	**18.3**	**17.7**	**14.1**	**15.0**		
**16**	***N. americanus***	**Undetermined**	**Undetermined**	**Undetermined**	**Undetermined**	**Positive**	**Positive**
	T.trichiura	**Undetermined**	**Undetermined**	**Undetermined**	**Undetermined**		
	A. lumbricoides	**18.0**	**19.8**	**14.5**	**14.3**		
**17**	***N. americanus***	**Undetermined**	**Undetermined**	**Undetermined**	**Undetermined**	**Positive**	**Positive**
	T.trichiura	**Undetermined**	**Undetermined**	**Undetermined**	**Undetermined**		
	A. lumbricoides	**12.6**	**12.5**	**9.0**	**8.5**		
**18**	***N. americanus***	**Undetermined**	**Undetermined**	**Undetermined**	**Undetermined**	**Positive**	**Positive**
	T.trichiura	**Undetermined**	**Undetermined**	**Undetermined**	**Undetermined**		
	A. lumbricoides	**15.1**	**15.7**	**10.4**	**9.9**		
**19**	***N. americanus***	**Undetermined**	**Undetermined**	**Undetermined**	**Undetermined**	**Positive**	**Positive**
	T.trichiura	**Undetermined**	**Undetermined**	**Undetermined**	**Undetermined**		
	A. lumbricoides	**19.5**	**21.8**	**14.5**	**14.7**		
**20**	***N. americanus***	**Undetermined**	**Undetermined**	**Undetermined**	**Undetermined**	**Positive**	**Positive**
	T. trichiura	**Undetermined**	**Undetermined**	**28.6**	**26.8**		
	A. lumbricoides	**13.9**	**16.6**	**9.9**	**10.2**		
**21**	***N. americanus***	**31.3**	**Undetermined**	**23.8**	**30.7**	**Positive**	**Positive**
	T. trichiura	**Undetermined**	**Undetermined**	**Undetermined**	**Undetermined**		
	A. lumbricoides	**Undetermined**	**36.2**	**Undetermined**	**Undetermined**		
**22**	***N. americanus***	**Undetermined**	**Undetermined**	**Undetermined**	**Undetermined**	**Positive**	**Positive**
	T.trichiura	**Undetermined**	**Undetermined**	**Undetermined**	**Undetermined**		
	A. lumbricoides	**13.2**	**15.2**	**9.8**	**10.3**		
**23**	***N. americanus***	**22.2**	**23.7**	**17.91**	**18.65**	**Positive**	**Positive**
	T.trichiura	**Undetermined**	**Undetermined**	**Undetermined**	**Undetermined**		
	A. lumbricoides	**19.3**	**19.2**	**15.1**	**15.4**		

**24**	***N. americanus***	**Undetermined**	**Undetermined**	**Undetermined**	**Undetermined**	**Positive**	**Positive**
	T.trichiura	**Undetermined**	**Undetermined**	**Undetermined**	**Undetermined**		
	A. lumbricoides	**13.1**	**13.0**	**8.8**	**8.9**		
**25**	***N. americanus***	**27.6**	**Undetermined**	**18.7**	**20.6**	**Positive**	**Positive**
	T.trichiura	**Undetermined**	**Undetermined**	**Undetermined**	**Undetermined**		
	A. lumbricoides	**18.8**	**19.2**	**11.3**	**12.4**		
**26**	***N. americanus***	**Undetermined**	**Undetermined**	**Undetermined**	**Undetermined**	**Positive**	**Positive**
	T.trichiura	**Undetermined**	**Undetermined**	**Undetermined**	**Undetermined**		
	A. lumbricoides	**34.4**	**31.1**	**31.6**	**26.6**		
**27**	***N. americanus***	**26.2**	**26.1**	**18.93**	**19.07**	**Positive**	**Positive**
	T.trichiura	**Undetermined**	**Undetermined**	**Undetermined**	**Undetermined**		
	A. lumbricoides	**Undetermined**	**Undetermined**	**Undetermined**	**Undetermined**		
**28**	***N. americanus***	**23.4**	**25.9**	**16.65**	**17.80**	**Positive**	**Positive**
	T.trichiura	**Undetermined**	**Undetermined**	**28.7**	**29.4**		
	A. lumbricoides	**Undetermined**	**Undetermined**	**Undetermined**	**Undetermined**		
**29**	***N. americanus***	**22.8**	**23.7**	**17.79**	**17.40**	**Positive**	**Positive**
	T.trichiura	**Undetermined**	**Undetermined**	**Undetermined**	**Undetermined**		
	A. lumbricoides	**Undetermined**	**Undetermined**	**Undetermined**	**Undetermined**		
**30**	***N. americanus***	**18.5**	**17.8**	**13.89**	**14.34**	**Positive**	**Positive**
	T.trichiura	**Undetermined**	**Undetermined**	**Undetermined**	**Undetermined**		
	A. lumbricoides	**Undetermined**	**31.2**	**Undetermined**	**32.4**		
**31**	***N. americanus***	**Undetermined**	**Undetermined**	**Undetermined**	**Undetermined**	**Positive**	**Positive**
	T.trichiura	**37.1**	**34.1**	**Undetermined**	**32.3**		
	A. lumbricoides	**Undetermined**	**Undetermined**	**Undetermined**	**Undetermined**		
**32**	***N. americanus***	**27.4**	**25.6**	**23.55**	**27.21**	**Positive**	**Positive**
	T.trichiura	**Undetermined**	**Undetermined**	**Undetermined**	**Undetermined**		
	A. lumbricoides	**Undetermined**	**Undetermined**	**Undetermined**	**Undetermined**		
**33**	***N. americanus***	**Undetermined**	**Undetermined**	**Undetermined**	**Undetermined**	**Positive**	**Positive**
	T.trichiura	**Undetermined**	**Undetermined**	**Undetermined**	**Undetermined**		
	A. lumbricoides	**19.0**	**22.9**	**14.9**	**14.7**		
**34**	***N. americanus***	**22.5**	**27.1**	**20.49**	**17.30**	**Positive**	**Positive**
	T.trichiura	**Undetermined**	**Undetermined**	**Undetermined**	**Undetermined**		
	A. lumbricoides	**Undetermined**	**Undetermined**	**Undetermined**	**Undetermined**		
**35**	***N. americanus***	**Undetermined**	**Undetermined**	**Undetermined**	**Undetermined**	**Negative**	**Positive**
	T.trichiura	**Undetermined**	**Undetermined**	**Undetermined**	**Undetermined**		
	A. lumbricoides	**Undetermined**	**Undetermined**	**33.9**	**32.2**		
**36**	***N. americanus***	**Undetermined**	**Undetermined**	**Undetermined**	**Undetermined**	**Positive (1/2)**	**Negative**
	T.trichiura	**Undetermined**	**Undetermined**	**Undetermined**	**Undetermined**		
	A. lumbricoides	**Undetermined**	**34.2**	**Undetermined**	**Undetermined**		

37	*N. americanus*	Undetermined	Undetermined	Undetermined	Undetermined	Negative	Positive (1/2)
	T.trichiura	Undetermined	Undetermined	Undetermined	Undetermined		
	A. lumbricoides	Undetermined	Undetermined	Undetermined	31.9		
38	*N. americanus*	28.8	25.7	28.4	Undetermined	Positive	Positive (1/2)
	T.trichiura	Undetermined	Undetermined	Undetermined	Undetermined		
	A. lumbricoides	Undetermined	Undetermined	Undetermined	Undetermined		
39	*N. americanus*	Undetermined	36.9	Undetermined	Undetermined	Positive (1/2)	Negative
	T.trichiura	Undetermined	Undetermined	Undetermined	Undetermined		
	A. lumbricoides	Undetermined	Undetermined	Undetermined	Undetermined		
40	*N. americanus*	Undetermined	Undetermined	Undetermined	Undetermined	Positive (1/2)	Negative
	T.trichiura	Undetermined	Undetermined	Undetermined	Undetermined		
	A. lumbricoides	Undetermined	36.2	Undetermined	Undetermined		
41	*N. americanus*	Undetermined	Undetermined	Undetermined	Undetermined	Positive (1/2)	Negative
	T.trichiura	Undetermined	Undetermined	Undetermined	Undetermined		
	A. lumbricoides	Undetermined	33.8	Undetermined	Undetermined		
42	*N. americanus*	Undetermined	32.5	Undetermined	Undetermined	Positive (1/2)	Negative
	T.trichiura	Undetermined	Undetermined	Undetermined	Undetermined		
	A. lumbricoides	Undetermined	Undetermined	Undetermined	Undetermined		
43	*N. americanus*	36.5	Undetermined	Undetermined	Undetermined	Positive (1/2)	Negative
	T.trichiura	Undetermined	Undetermined	Undetermined	Undetermined		
	A. lumbricoides	Undetermined	Undetermined	Undetermined	Undetermined		
44	*N. americanus*	Undetermined	Undetermined	Undetermined	Undetermined	Positive (1/2)	Negative
	T.trichiura	Undetermined	Undetermined	Undetermined	Undetermined		
	A. lumbricoides	Undetermined	31.5	Undetermined	Undetermined		
45	*N. americanus*	Undetermined	Undetermined	Undetermined	Undetermined	Positive (1/2)	Negative
	T.trichiura	Undetermined	Undetermined	Undetermined	Undetermined		
	A. lumbricoides	Undetermined	20.0	Undetermined	Undetermined		
46	*N. americanus*	Undetermined	Undetermined	33.2	Undetermined	Negative	Positive (1/2)
	T.trichiura	Undetermined	Undetermined	Undetermined	Undetermined		
	A. lumbricoides	Undetermined	Undetermined	Undetermined	Undetermined		
47	*N. americanus*	Undetermined	Undetermined	Undetermined	Undetermined	Negative	Positive (1/2)
	T.trichiura	Undetermined	Undetermined	Undetermined	Undetermined		
	A. lumbricoides	Undetermined	Undetermined	Undetermined	35.2		
48	*N. americanus*	Undetermined	36.9	Undetermined	Undetermined	Positive(1/2)	Negative
	T.trichiura	Undetermined	Undetermined	Undetermined	Undetermined		
	A. lumbricoides	Undetermined	Undetermined	Undetermined	Undetermined		

Figure A7.2: *All line data for the 200 sample experiment extractions*

		Quantigen		Smith	
**Sample ID**	**Species**	**Extract 1**	**Extract 2**	**Extract 3**	**Extract 4**
1	*N. americanus*	25.3	24.2	18.13	18.41
	T.trichiura	Undetermined	Undetermined	Undetermined	Undetermined
	A. lumbricoides	Undetermined	Undetermined	Undetermined	Undetermined
2	*N. americanus*	36.3	35.9	32.4	26.7
	T.trichiura	Undetermined	Undetermined	Undetermined	Undetermined
	A. lumbricoides	Undetermined	Undetermined	Undetermined	Undetermined
3	*N. americanus*	23.6	23.5	21.11	17.29
	T.trichiura	Undetermined	Undetermined	Undetermined	Undetermined
	A. lumbricoides	Undetermined	Undetermined	Undetermined	Undetermined
4	*N. americanus*	28.4	28.6	23.91	32.14
	T.trichiura	Undetermined	Undetermined	Undetermined	Undetermined
	A. lumbricoides	Undetermined	Undetermined	Undetermined	Undetermined
5	*N. americanus*	28.3	27.2	23.68	24.36
	T.trichiura	Undetermined	Undetermined	Undetermined	Undetermined
	A. lumbricoides	Undetermined	Undetermined	Undetermined	Undetermined
6	*N. americanus*	26.9	28.5	20.77	22.83
	T.trichiura	Undetermined	Undetermined	Undetermined	Undetermined
	A. lumbricoides	Undetermined	Undetermined	Undetermined	Undetermined
7	*N. americanus*	36.7	36.7	36.2	29.1
	T.trichiura	Undetermined	Undetermined	Undetermined	Undetermined
	A. lumbricoides	Undetermined	Undetermined	Undetermined	Undetermined
8	*N. americanus*	33.3	34.1	30.46	29.98
	T.trichiura	Undetermined	Undetermined	Undetermined	Undetermined
	A. lumbricoides	Undetermined	Undetermined	Undetermined	Undetermined
9	*N. americanus*	Undetermined	Undetermined	Undetermined	Undetermined
	T.trichiura	Undetermined	Undetermined	Undetermined	Undetermined
	A. lumbricoides	19.8	20.8	15.6	15.2
10	*N. americanus*	24.7	22.9	17.30	20.57
	T.trichiura	Undetermined	Undetermined	Undetermined	Undetermined
	A. lumbricoides	Undetermined	Undetermined	Undetermined	Undetermined
11	*N. americanus*	Undetermined	Undetermined	Undetermined	Undetermined
	T.trichiura	Undetermined	Undetermined	Undetermined	Undetermined
	A. lumbricoides	18.5	19.1	14.4	14.9
12	*N. americanus*	Undetermined	Undetermined	Undetermined	Undetermined
	T.trichiura	Undetermined	Undetermined	Undetermined	Undetermined
	A. lumbricoides	34.7	37.0	32.6	32.1

**13**	***N. americanus***	**25.1**	**31.0**	**19.69**	**18.99**
	T.trichiura	**Undetermined**	**Undetermined**	**Undetermined**	**Undetermined**
	A. lumbricoides	**Undetermined**	**Undetermined**	**Undetermined**	**Undetermined**
**14**	***N. americanus***	**Undetermined**	**Undetermined**	**Undetermined**	**Undetermined**
	T.trichiura	**Undetermined**	**Undetermined**	**Undetermined**	**Undetermined**
	A. lumbricoides	**11.7**	**12.3**	**7.8**	**9.3**
**15**	***N. americanus***	**Undetermined**	**Undetermined**	**Undetermined**	**Undetermined**
	T.trichiura	**Undetermined**	**Undetermined**	**Undetermined**	**Undetermined**
	A. lumbricoides	**18.3**	**17.7**	**14.1**	**15.0**
**16**	***N. americanus***	**Undetermined**	**Undetermined**	**Undetermined**	**Undetermined**
	T.trichiura	**Undetermined**	**Undetermined**	**Undetermined**	**Undetermined**
	A. lumbricoides	**18.0**	**19.8**	**14.5**	**14.3**
**17**	***N. americanus***	**Undetermined**	**Undetermined**	**Undetermined**	**Undetermined**
	T.trichiura	**Undetermined**	**Undetermined**	**Undetermined**	**Undetermined**
	A. lumbricoides	**12.6**	**12.5**	**9.0**	**8.5**
**18**	***N. americanus***	**Undetermined**	**Undetermined**	**Undetermined**	**Undetermined**
	T.trichiura	**Undetermined**	**Undetermined**	**Undetermined**	**Undetermined**
	A. lumbricoides	**15.1**	**15.7**	**10.4**	**9.9**
**19**	***N. americanus***	**Undetermined**	**Undetermined**	**Undetermined**	**Undetermined**
	T.trichiura	**Undetermined**	**Undetermined**	**Undetermined**	**Undetermined**
	A. lumbricoides	**19.5**	**21.8**	**14.5**	**14.7**
**20**	***N. americanus***	**Undetermined**	**Undetermined**	**Undetermined**	**Undetermined**
	T.trichiura	**Undetermined**	**Undetermined**	**28.6**	**26.8**
	A. lumbricoides	**13.9**	**16.6**	**9.9**	**10.2**
**21**	***N. americanus***	**31.3**	**Undetermined**	**23.8**	**30.7**
	T.trichiura	**Undetermined**	**Undetermined**	**Undetermined**	**Undetermined**
	A. lumbricoides	**Undetermined**	**36.2**	**Undetermined**	**Undetermined**
**22**	***N. americanus***	**Undetermined**	**Undetermined**	**Undetermined**	**Undetermined**
	T.trichiura	**Undetermined**	**Undetermined**	**Undetermined**	**Undetermined**
	A. lumbricoides	**13.2**	**15.2**	**9.8**	**10.3**
**23**	***N. americanus***	**22.2**	**23.7**	**17.91**	**18.65**
	T.trichiura	**Undetermined**	**Undetermined**	**Undetermined**	**Undetermined**
	A. lumbricoides	**19.3**	**19.2**	**15.1**	**15.4**
**24**	***N. americanus***	**Undetermined**	**Undetermined**	**Undetermined**	**Undetermined**
	T.trichiura	**Undetermined**	**Undetermined**	**Undetermined**	**Undetermined**
	A. lumbricoides	**13.1**	**13.0**	**8.8**	**8.9**
**25**	***N. americanus***	**27.6**	**Undetermined**	**18.7**	**20.6**
	T.trichiura	**Undetermined**	**Undetermined**	**Undetermined**	**Undetermined**
	A. lumbricoides	**18.8**	**19.2**	**11.3**	**12.4**
**26**	***N. americanus***	**Undetermined**	**Undetermined**	**Undetermined**	**Undetermined**
	T.trichiura	**Undetermined**	**Undetermined**	**Undetermined**	**Undetermined**
	A. lumbricoides	**34.4**	**31.1**	**31.6**	**26.6**

27	*N. americanus*	26.2	26.1	18.93	19.07
	T.trichiura	Undetermined	Undetermined	Undetermined	Undetermined
	A. lumbricoides	Undetermined	Undetermined	Undetermined	Undetermined
28	*N. americanus*	23.4	25.9	16.65	17.80
	T.trichiura	Undetermined	Undetermined	28.7	29.4
	A. lumbricoides	Undetermined	Undetermined	Undetermined	Undetermined
29	*N. americanus*	22.8	23.7	17.79	17.40
	T.trichiura	Undetermined	Undetermined	Undetermined	Undetermined
	A. lumbricoides	Undetermined	Undetermined	Undetermined	Undetermined
30	*N. americanus*	18.5	17.8	13.89	14.34
	T.trichiura	Undetermined	Undetermined	Undetermined	Undetermined
	A. lumbricoides	Undetermined	31.2	Undetermined	32.4
31	*N. americanus*	Undetermined	Undetermined	Undetermined	Undetermined
	T.trichiura	37.1	34.1	Undetermined	32.3
	A. lumbricoides	Undetermined	Undetermined	Undetermined	Undetermined
32	*N. americanus*	27.4	25.6	23.55	27.21
	T.trichiura	Undetermined	Undetermined	Undetermined	Undetermined
	A. lumbricoides	Undetermined	Undetermined	Undetermined	Undetermined
33	*N. americanus*	Undetermined	Undetermined	Undetermined	Undetermined
	T.trichiura	Undetermined	Undetermined	Undetermined	Undetermined
	A. lumbricoides	19.0	22.9	14.9	14.7
34	*N. americanus*	22.5	27.1	20.49	17.30
	T.trichiura	Undetermined	Undetermined	Undetermined	Undetermined
	A. lumbricoides	Undetermined	Undetermined	Undetermined	Undetermined
35	*N. americanus*	Undetermined	Undetermined	Undetermined	Undetermined
	T.trichiura	Undetermined	Undetermined	Undetermined	Undetermined
	A. lumbricoides	Undetermined	Undetermined	33.9	32.2
36	*N. americanus*	Undetermined	Undetermined	Undetermined	Undetermined
	T.trichiura	Undetermined	Undetermined	Undetermined	Undetermined
	A. lumbricoides	Undetermined	34.2	Undetermined	Undetermined
37	*N. americanus*	Undetermined	Undetermined	Undetermined	Undetermined
	T.trichiura	Undetermined	Undetermined	Undetermined	Undetermined
	A. lumbricoides	Undetermined	Undetermined	Undetermined	31.9
38	*N. americanus*	28.8	25.7	28.4	Undetermined
	T.trichiura	Undetermined	Undetermined	Undetermined	Undetermined
	A. lumbricoides	Undetermined	Undetermined	Undetermined	Undetermined
39	*N. americanus*	Undetermined	36.9	Undetermined	Undetermined
	T.trichiura	Undetermined	Undetermined	Undetermined	Undetermined
	A. lumbricoides	Undetermined	Undetermined	Undetermined	Undetermined

40	*N. americanus*	Undetermined	Undetermined	Undetermined	Undetermined
	T.trichiura	Undetermined	Undetermined	Undetermined	Undetermined
	A. lumbricoides	Undetermined	36.2	Undetermined	Undetermined
41	*N. americanus*	Undetermined	Undetermined	Undetermined	Undetermined
	T.trichiura	Undetermined	Undetermined	Undetermined	Undetermined
	A. lumbricoides	Undetermined	33.8	Undetermined	Undetermined
42	*N. americanus*	Undetermined	32.5	Undetermined	Undetermined
	T.trichiura	Undetermined	Undetermined	Undetermined	Undetermined
	A. lumbricoides	Undetermined	Undetermined	Undetermined	Undetermined
43	*N. americanus*	36.5	Undetermined	Undetermined	Undetermined
	T.trichiura	Undetermined	Undetermined	Undetermined	Undetermined
	A. lumbricoides	Undetermined	Undetermined	Undetermined	Undetermined
44	*N. americanus*	Undetermined	Undetermined	Undetermined	Undetermined
	T.trichiura	Undetermined	Undetermined	Undetermined	Undetermined
	A. lumbricoides	Undetermined	31.5	Undetermined	Undetermined
45	*N. americanus*	Undetermined	Undetermined	Undetermined	Undetermined
	T.trichiura	Undetermined	Undetermined	Undetermined	Undetermined
	A. lumbricoides	Undetermined	20.0	Undetermined	Undetermined
46	*N. americanus*	Undetermined	Undetermined	33.2	Undetermined
	T.trichiura	Undetermined	Undetermined	Undetermined	Undetermined
	A. lumbricoides	Undetermined	Undetermined	Undetermined	Undetermined
47	*N. americanus*	Undetermined	Undetermined	Undetermined	Undetermined
	T.trichiura	Undetermined	Undetermined	Undetermined	Undetermined
	A. lumbricoides	Undetermined	Undetermined	Undetermined	35.2
48	*N. americanus*	Undetermined	36.9	Undetermined	Undetermined
	T.trichiura	Undetermined	Undetermined	Undetermined	Undetermined
	A. lumbricoides	Undetermined	Undetermined	Undetermined	Undetermined
49	*N. americanus*	Undetermined	Undetermined	Undetermined	Undetermined
	T.trichiura	Undetermined	Undetermined	Undetermined	Undetermined
	A. lumbricoides	Undetermined	Undetermined	Undetermined	Undetermined
50	*N. americanus*	Undetermined	Undetermined	Undetermined	Undetermined
	T.trichiura	Undetermined	Undetermined	Undetermined	Undetermined
	A. lumbricoides	Undetermined	Undetermined	Undetermined	Undetermined
51	*N. americanus*	Undetermined	Undetermined	Undetermined	Undetermined
	T.trichiura	Undetermined	Undetermined	Undetermined	Undetermined
	A. lumbricoides	Undetermined	Undetermined	Undetermined	Undetermined
52	*N. americanus*	Undetermined	Undetermined	Undetermined	Undetermined
	T.trichiura	Undetermined	Undetermined	Undetermined	Undetermined
	A. lumbricoides	Undetermined	Undetermined	Undetermined	Undetermined

53	***N. americanus***	**Undetermined**	**Undetermined**	**Undetermined**	**Undetermined**
	T.trichiura	**Undetermined**	**Undetermined**	**Undetermined**	**Undetermined**
	A. lumbricoides	**Undetermined**	**Undetermined**	**Undetermined**	**Undetermined**
54	***N. americanus***	**Undetermined**	**Undetermined**	**Undetermined**	**Undetermined**
	T.trichiura	**Undetermined**	**Undetermined**	**Undetermined**	**Undetermined**
	A. lumbricoides	**Undetermined**	**Undetermined**	**Undetermined**	**Undetermined**
55	***N. americanus***	**Undetermined**	**Undetermined**	**Undetermined**	**Undetermined**
	T.trichiura	**Undetermined**	**Undetermined**	**Undetermined**	**Undetermined**
	A. lumbricoides	**Undetermined**	**Undetermined**	**Undetermined**	**Undetermined**
56	***N. americanus***	**Undetermined**	**Undetermined**	**Undetermined**	**Undetermined**
	T.trichiura	**Undetermined**	**Undetermined**	**Undetermined**	**Undetermined**
	A. lumbricoides	**Undetermined**	**Undetermined**	**Undetermined**	**Undetermined**
57	***N. americanus***	**Undetermined**	**Undetermined**	**Undetermined**	**Undetermined**
	T.trichiura	**Undetermined**	**Undetermined**	**Undetermined**	**Undetermined**
	A. lumbricoides	**Undetermined**	**Undetermined**	**Undetermined**	**Undetermined**
58	***N. americanus***	**Undetermined**	**Undetermined**	**Undetermined**	**Undetermined**
	T.trichiura	**Undetermined**	**Undetermined**	**Undetermined**	**Undetermined**
	A. lumbricoides	**Undetermined**	**Undetermined**	**Undetermined**	**Undetermined**
59	***N. americanus***	**Undetermined**	**Undetermined**	**Undetermined**	**Undetermined**
	T.trichiura	**Undetermined**	**Undetermined**	**Undetermined**	**Undetermined**
	A. lumbricoides	**Undetermined**	**Undetermined**	**Undetermined**	**Undetermined**
60	***N. americanus***	**Undetermined**	**Undetermined**	**Undetermined**	**Undetermined**
	T.trichiura	**Undetermined**	**Undetermined**	**Undetermined**	**Undetermined**
	A. lumbricoides	**Undetermined**	**Undetermined**	**Undetermined**	**Undetermined**
61	***N. americanus***	**Undetermined**	**Undetermined**	**Undetermined**	**Undetermined**
	T.trichiura	**Undetermined**	**Undetermined**	**Undetermined**	**Undetermined**
	A. lumbricoides	**Undetermined**	**Undetermined**	**Undetermined**	**Undetermined**
62	***N. americanus***	**Undetermined**	**Undetermined**	**Undetermined**	**Undetermined**
	T.trichiura	**Undetermined**	**Undetermined**	**Undetermined**	**Undetermined**
	A. lumbricoides	**Undetermined**	**Undetermined**	**Undetermined**	**Undetermined**
63	***N. americanus***	**Undetermined**	**Undetermined**	**Undetermined**	**Undetermined**
	T.trichiura	**Undetermined**	**Undetermined**	**Undetermined**	**Undetermined**
	A. lumbricoides	**Undetermined**	**Undetermined**	**Undetermined**	**Undetermined**
64	***N. americanus***	**Undetermined**	**Undetermined**	**Undetermined**	**Undetermined**
	T.trichiura	**Undetermined**	**Undetermined**	**Undetermined**	**Undetermined**
	A. lumbricoides	**Undetermined**	**Undetermined**	**Undetermined**	**Undetermined**
65	***N. americanus***	**Undetermined**	**Undetermined**	**Undetermined**	**Undetermined**
	T.trichiura	**Undetermined**	**Undetermined**	**Undetermined**	**Undetermined**
	A. lumbricoides	**Undetermined**	**Undetermined**	**Undetermined**	**Undetermined**

**66**	***N. americanus***	**Undetermined**	**Undetermined**	**Undetermined**	**Undetermined**
	T.trichiura	**Undetermined**	**Undetermined**	**Undetermined**	**Undetermined**
	A. lumbricoides	**Undetermined**	**Undetermined**	**Undetermined**	**Undetermined**
**67**	***N. americanus***	**Undetermined**	**Undetermined**	**Undetermined**	**Undetermined**
	T.trichiura	**Undetermined**	**Undetermined**	**Undetermined**	**Undetermined**
	A. lumbricoides	**Undetermined**	**Undetermined**	**Undetermined**	**Undetermined**
**68**	***N. americanus***	**Undetermined**	**Undetermined**	**Undetermined**	**Undetermined**
	T.trichiura	**Undetermined**	**Undetermined**	**Undetermined**	**Undetermined**
	A. lumbricoides	**Undetermined**	**Undetermined**	**Undetermined**	**Undetermined**
**69**	***N. americanus***	**Undetermined**	**Undetermined**	**Undetermined**	**Undetermined**
	T.trichiura	**Undetermined**	**Undetermined**	**Undetermined**	**Undetermined**
	A. lumbricoides	**Undetermined**	**Undetermined**	**Undetermined**	**Undetermined**
**70**	***N. americanus***	**Undetermined**	**Undetermined**	**Undetermined**	**Undetermined**
	T.trichiura	**Undetermined**	**Undetermined**	**Undetermined**	**Undetermined**
	A. lumbricoides	**Undetermined**	**Undetermined**	**Undetermined**	**Undetermined**
**71**	***N. americanus***	**Undetermined**	**Undetermined**	**Undetermined**	**Undetermined**
	T.trichiura	**Undetermined**	**Undetermined**	**Undetermined**	**Undetermined**
	A. lumbricoides	**Undetermined**	**Undetermined**	**Undetermined**	**Undetermined**
**72**	***N. americanus***	**Undetermined**	**Undetermined**	**Undetermined**	**Undetermined**
	T.trichiura	**Undetermined**	**Undetermined**	**Undetermined**	**Undetermined**
	A. lumbricoides	**Undetermined**	**Undetermined**	**Undetermined**	**Undetermined**
**73**	***N. americanus***	**Undetermined**	**Undetermined**	**Undetermined**	**Undetermined**
	T.trichiura	**Undetermined**	**Undetermined**	**Undetermined**	**Undetermined**
	A. lumbricoides	**Undetermined**	**Undetermined**	**Undetermined**	**Undetermined**
**74**	***N. americanus***	**Undetermined**	**Undetermined**	**Undetermined**	**Undetermined**
	T.trichiura	**Undetermined**	**Undetermined**	**Undetermined**	**Undetermined**
	A. lumbricoides	**Undetermined**	**Undetermined**	**Undetermined**	**Undetermined**
**75**	***N. americanus***	**Undetermined**	**Undetermined**	**Undetermined**	**Undetermined**
	T.trichiura	**Undetermined**	**Undetermined**	**Undetermined**	**Undetermined**
	A. lumbricoides	**Undetermined**	**Undetermined**	**Undetermined**	**Undetermined**
**76**	***N. americanus***	**Undetermined**	**Undetermined**	**Undetermined**	**Undetermined**
	T.trichiura	**Undetermined**	**Undetermined**	**Undetermined**	**Undetermined**
	A. lumbricoides	**Undetermined**	**Undetermined**	**Undetermined**	**Undetermined**
**77**	***N. americanus***	**Undetermined**	**Undetermined**	**Undetermined**	**Undetermined**
	T.trichiura	**Undetermined**	**Undetermined**	**Undetermined**	**Undetermined**
	A. lumbricoides	**Undetermined**	**Undetermined**	**Undetermined**	**Undetermined**
**78**	***N. americanus***	**Undetermined**	**Undetermined**	**Undetermined**	**Undetermined**
	T.trichiura	**Undetermined**	**Undetermined**	**Undetermined**	**Undetermined**
	A. lumbricoides	**Undetermined**	**Undetermined**	**Undetermined**	**Undetermined**

**79**	***N. americanus***	**Undetermined**	**Undetermined**	**Undetermined**	**Undetermined**
	T.trichiura	**Undetermined**	**Undetermined**	**Undetermined**	**Undetermined**
	A. lumbricoides	**Undetermined**	**Undetermined**	**Undetermined**	**Undetermined**
**80**	***N. americanus***	**Undetermined**	**Undetermined**	**Undetermined**	**Undetermined**
	T.trichiura	**Undetermined**	**Undetermined**	**Undetermined**	**Undetermined**
	A. lumbricoides	**Undetermined**	**Undetermined**	**Undetermined**	**Undetermined**
**81**	***N. americanus***	**Undetermined**	**Undetermined**	**Undetermined**	**Undetermined**
	T.trichiura	**Undetermined**	**Undetermined**	**Undetermined**	**Undetermined**
	A. lumbricoides	**Undetermined**	**Undetermined**	**Undetermined**	**Undetermined**
**82**	***N. americanus***	**Undetermined**	**Undetermined**	**Undetermined**	**Undetermined**
	T.trichiura	**Undetermined**	**Undetermined**	**Undetermined**	**Undetermined**
	A. lumbricoides	**Undetermined**	**Undetermined**	**Undetermined**	**Undetermined**
**83**	***N. americanus***	**Undetermined**	**Undetermined**	**Undetermined**	**Undetermined**
	T.trichiura	**Undetermined**	**Undetermined**	**Undetermined**	**Undetermined**
	A. lumbricoides	**Undetermined**	**Undetermined**	**Undetermined**	**Undetermined**
**84**	***N. americanus***	**Undetermined**	**Undetermined**	**Undetermined**	**Undetermined**
	T.trichiura	**Undetermined**	**Undetermined**	**Undetermined**	**Undetermined**
	A. lumbricoides	**Undetermined**	**Undetermined**	**Undetermined**	**Undetermined**
**85**	***N. americanus***	**Undetermined**	**Undetermined**	**Undetermined**	**Undetermined**
	T.trichiura	**Undetermined**	**Undetermined**	**Undetermined**	**Undetermined**
	A. lumbricoides	**Undetermined**	**Undetermined**	**Undetermined**	**Undetermined**
**86**	***N. americanus***	**Undetermined**	**Undetermined**	**Undetermined**	**Undetermined**
	T.trichiura	**Undetermined**	**Undetermined**	**Undetermined**	**Undetermined**
	A. lumbricoides	**Undetermined**	**Undetermined**	**Undetermined**	**Undetermined**
**87**	***N. americanus***	**Undetermined**	**Undetermined**	**Undetermined**	**Undetermined**
	T.trichiura	**Undetermined**	**Undetermined**	**Undetermined**	**Undetermined**
	A. lumbricoides	**Undetermined**	**Undetermined**	**Undetermined**	**Undetermined**
**88**	***N. americanus***	**Undetermined**	**Undetermined**	**Undetermined**	**Undetermined**
	T.trichiura	**Undetermined**	**Undetermined**	**Undetermined**	**Undetermined**
	A. lumbricoides	**Undetermined**	**Undetermined**	**Undetermined**	**Undetermined**
**89**	***N. americanus***	**Undetermined**	**Undetermined**	**Undetermined**	**Undetermined**
	T.trichiura	**Undetermined**	**Undetermined**	**Undetermined**	**Undetermined**
	A. lumbricoides	**Undetermined**	**Undetermined**	**Undetermined**	**Undetermined**
**90**	***N. americanus***	**Undetermined**	**Undetermined**	**Undetermined**	**Undetermined**
	T.trichiura	**Undetermined**	**Undetermined**	**Undetermined**	**Undetermined**
	A. lumbricoides	**Undetermined**	**Undetermined**	**Undetermined**	**Undetermined**
**91**	***N. americanus***	**Undetermined**	**Undetermined**	**Undetermined**	**Undetermined**
	T.trichiura	**Undetermined**	**Undetermined**	**Undetermined**	**Undetermined**
	A. lumbricoides	**Undetermined**	**Undetermined**	**Undetermined**	**Undetermined**

**92**	***N. americanus***	**Undetermined**	**Undetermined**	**Undetermined**	**Undetermined**
	T.trichiura	**Undetermined**	**Undetermined**	**Undetermined**	**Undetermined**
	A. lumbricoides	**Undetermined**	**Undetermined**	**Undetermined**	**Undetermined**
**93**	***N. americanus***	**Undetermined**	**Undetermined**	**Undetermined**	**Undetermined**
	T.trichiura	**Undetermined**	**Undetermined**	**Undetermined**	**Undetermined**
	A. lumbricoides	**Undetermined**	**Undetermined**	**Undetermined**	**Undetermined**
**94**	***N. americanus***	**Undetermined**	**Undetermined**	**Undetermined**	**Undetermined**
	T.trichiura	**Undetermined**	**Undetermined**	**Undetermined**	**Undetermined**
	A. lumbricoides	**Undetermined**	**Undetermined**	**Undetermined**	**Undetermined**
**95**	***N. americanus***	**Undetermined**	**Undetermined**	**Undetermined**	**Undetermined**
	T.trichiura	**Undetermined**	**Undetermined**	**Undetermined**	**Undetermined**
	A. lumbricoides	**Undetermined**	**Undetermined**	**Undetermined**	**Undetermined**
**96**	***N. americanus***	**Undetermined**	**Undetermined**	**Undetermined**	**Undetermined**
	T.trichiura	**Undetermined**	**Undetermined**	**Undetermined**	**Undetermined**
	A. lumbricoides	**Undetermined**	**Undetermined**	**Undetermined**	**Undetermined**
**97**	***N. americanus***	**Undetermined**	**Undetermined**	**Undetermined**	**Undetermined**
	T.trichiura	**Undetermined**	**Undetermined**	**Undetermined**	**Undetermined**
	A. lumbricoides	**Undetermined**	**Undetermined**	**Undetermined**	**Undetermined**
**98**	***N. americanus***	**Undetermined**	**Undetermined**	**Undetermined**	**Undetermined**
	T.trichiura	**Undetermined**	**Undetermined**	**Undetermined**	**Undetermined**
	A. lumbricoides	**Undetermined**	**Undetermined**	**Undetermined**	**Undetermined**
**99**	***N. americanus***	**Undetermined**	**Undetermined**	**Undetermined**	**Undetermined**
	T.trichiura	**Undetermined**	**Undetermined**	**Undetermined**	**Undetermined**
	A. lumbricoides	**Undetermined**	**Undetermined**	**Undetermined**	**Undetermined**
**100**	***N. americanus***	**Undetermined**	**Undetermined**	**Undetermined**	**Undetermined**
	T.trichiura	**Undetermined**	**Undetermined**	**Undetermined**	**Undetermined**
	A. lumbricoides	**Undetermined**	**Undetermined**	**Undetermined**	**Undetermined**
**101**	***N. americanus***	**Undetermined**	**Undetermined**	**Undetermined**	**Undetermined**
	T.trichiura	**Undetermined**	**Undetermined**	**Undetermined**	**Undetermined**
	A. lumbricoides	**Undetermined**	**Undetermined**	**Undetermined**	**Undetermined**
**102**	***N. americanus***	**Undetermined**	**Undetermined**	**Undetermined**	**Undetermined**
	T.trichiura	**Undetermined**	**Undetermined**	**Undetermined**	**Undetermined**
	A. lumbricoides	**Undetermined**	**Undetermined**	**Undetermined**	**Undetermined**
**103**	***N. americanus***	**Undetermined**	**Undetermined**	**Undetermined**	**Undetermined**
	T.trichiura	**Undetermined**	**Undetermined**	**Undetermined**	**Undetermined**
	A. lumbricoides	**Undetermined**	**Undetermined**	**Undetermined**	**Undetermined**
**104**	***N. americanus***	**Undetermined**	**Undetermined**	**Undetermined**	**Undetermined**
	T.trichiura	**Undetermined**	**Undetermined**	**Undetermined**	**Undetermined**
	A. lumbricoides	**Undetermined**	**Undetermined**	**Undetermined**	**Undetermined**

**105**	***N. americanus***	**Undetermined**	**Undetermined**	**Undetermined**	**Undetermined**
	T.trichiura	**Undetermined**	**Undetermined**	**Undetermined**	**Undetermined**
	A. lumbricoides	**Undetermined**	**Undetermined**	**Undetermined**	**Undetermined**
**106**	***N. americanus***	**Undetermined**	**Undetermined**	**Undetermined**	**Undetermined**
	T.trichiura	**Undetermined**	**Undetermined**	**Undetermined**	**Undetermined**
	A. lumbricoides	**Undetermined**	**Undetermined**	**Undetermined**	**Undetermined**
**107**	***N. americanus***	**Undetermined**	**Undetermined**	**Undetermined**	**Undetermined**
	T.trichiura	**Undetermined**	**Undetermined**	**Undetermined**	**Undetermined**
	A. lumbricoides	**Undetermined**	**Undetermined**	**Undetermined**	**Undetermined**
**108**	***N. americanus***	**Undetermined**	**Undetermined**	**Undetermined**	**Undetermined**
	T.trichiura	**Undetermined**	**Undetermined**	**Undetermined**	**Undetermined**
	A. lumbricoides	**Undetermined**	**Undetermined**	**Undetermined**	**Undetermined**
**109**	***N. americanus***	**Undetermined**	**Undetermined**	**Undetermined**	**Undetermined**
	T.trichiura	**Undetermined**	**Undetermined**	**Undetermined**	**Undetermined**
	A. lumbricoides	**Undetermined**	**Undetermined**	**Undetermined**	**Undetermined**
**110**	***N. americanus***	**Undetermined**	**Undetermined**	**Undetermined**	**Undetermined**
	T.trichiura	**Undetermined**	**Undetermined**	**Undetermined**	**Undetermined**
	A. lumbricoides	**Undetermined**	**Undetermined**	**Undetermined**	**Undetermined**
**111**	***N. americanus***	**Undetermined**	**Undetermined**	**Undetermined**	**Undetermined**
	T.trichiura	**Undetermined**	**Undetermined**	**Undetermined**	**Undetermined**
	A. lumbricoides	**Undetermined**	**Undetermined**	**Undetermined**	**Undetermined**
**112**	***N. americanus***	**Undetermined**	**Undetermined**	**Undetermined**	**Undetermined**
	T.trichiura	**Undetermined**	**Undetermined**	**Undetermined**	**Undetermined**
	A. lumbricoides	**Undetermined**	**Undetermined**	**Undetermined**	**Undetermined**
**113**	***N. americanus***	**Undetermined**	**Undetermined**	**Undetermined**	**Undetermined**
	T.trichiura	**Undetermined**	**Undetermined**	**Undetermined**	**Undetermined**
	A. lumbricoides	**Undetermined**	**Undetermined**	**Undetermined**	**Undetermined**
**114**	***N. americanus***	**Undetermined**	**Undetermined**	**Undetermined**	**Undetermined**
	T.trichiura	**Undetermined**	**Undetermined**	**Undetermined**	**Undetermined**
	A. lumbricoides	**Undetermined**	**Undetermined**	**Undetermined**	**Undetermined**
**115**	***N. americanus***	**Undetermined**	**Undetermined**	**Undetermined**	**Undetermined**
	T.trichiura	**Undetermined**	**Undetermined**	**Undetermined**	**Undetermined**
	A. lumbricoides	**Undetermined**	**Undetermined**	**Undetermined**	**Undetermined**
**116**	***N. americanus***	**Undetermined**	**Undetermined**	**Undetermined**	**Undetermined**
	T.trichiura	**Undetermined**	**Undetermined**	**Undetermined**	**Undetermined**
	A. lumbricoides	**Undetermined**	**Undetermined**	**Undetermined**	**Undetermined**
**117**	***N. americanus***	**Undetermined**	**Undetermined**	**Undetermined**	**Undetermined**
	T.trichiura	**Undetermined**	**Undetermined**	**Undetermined**	**Undetermined**
	A. lumbricoides	**Undetermined**	**Undetermined**	**Undetermined**	**Undetermined**

**118**	***N. americanus***	**Undetermined**	**Undetermined**	**Undetermined**	**Undetermined**
	T.trichiura	**Undetermined**	**Undetermined**	**Undetermined**	**Undetermined**
	A. lumbricoides	**Undetermined**	**Undetermined**	**Undetermined**	**Undetermined**
**119**	***N. americanus***	**Undetermined**	**Undetermined**	**Undetermined**	**Undetermined**
	T.trichiura	**Undetermined**	**Undetermined**	**Undetermined**	**Undetermined**
	A. lumbricoides	**Undetermined**	**Undetermined**	**Undetermined**	**Undetermined**
**120**	***N. americanus***	**Undetermined**	**Undetermined**	**Undetermined**	**Undetermined**
	T.trichiura	**Undetermined**	**Undetermined**	**Undetermined**	**Undetermined**
	A. lumbricoides	**Undetermined**	**Undetermined**	**Undetermined**	**Undetermined**
**121**	***N. americanus***	**Undetermined**	**Undetermined**	**Undetermined**	**Undetermined**
	T.trichiura	**Undetermined**	**Undetermined**	**Undetermined**	**Undetermined**
	A. lumbricoides	**Undetermined**	**Undetermined**	**Undetermined**	**Undetermined**
**122**	***N. americanus***	**Undetermined**	**Undetermined**	**Undetermined**	**Undetermined**
	T.trichiura	**Undetermined**	**Undetermined**	**Undetermined**	**Undetermined**
	A. lumbricoides	**Undetermined**	**Undetermined**	**Undetermined**	**Undetermined**
**123**	***N. americanus***	**Undetermined**	**Undetermined**	**Undetermined**	**Undetermined**
	T.trichiura	**Undetermined**	**Undetermined**	**Undetermined**	**Undetermined**
	A. lumbricoides	**Undetermined**	**Undetermined**	**Undetermined**	**Undetermined**
**124**	***N. americanus***	**Undetermined**	**Undetermined**	**Undetermined**	**Undetermined**
	T.trichiura	**Undetermined**	**Undetermined**	**Undetermined**	**Undetermined**
	A. lumbricoides	**Undetermined**	**Undetermined**	**Undetermined**	**Undetermined**
**125**	***N. americanus***	**Undetermined**	**Undetermined**	**Undetermined**	**Undetermined**
	T.trichiura	**Undetermined**	**Undetermined**	**Undetermined**	**Undetermined**
	A. lumbricoides	**Undetermined**	**Undetermined**	**Undetermined**	**Undetermined**
**126**	***N. americanus***	**Undetermined**	**Undetermined**	**Undetermined**	**Undetermined**
	T.trichiura	**Undetermined**	**Undetermined**	**Undetermined**	**Undetermined**
	A. lumbricoides	**Undetermined**	**Undetermined**	**Undetermined**	**Undetermined**
**127**	***N. americanus***	**Undetermined**	**Undetermined**	**Undetermined**	**Undetermined**
	T.trichiura	**Undetermined**	**Undetermined**	**Undetermined**	**Undetermined**
	A. lumbricoides	**Undetermined**	**Undetermined**	**Undetermined**	**Undetermined**
**128**	***N. americanus***	**Undetermined**	**Undetermined**	**Undetermined**	**Undetermined**
	T.trichiura	**Undetermined**	**Undetermined**	**Undetermined**	**Undetermined**
	A. lumbricoides	**Undetermined**	**Undetermined**	**Undetermined**	**Undetermined**
**129**	***N. americanus***	**Undetermined**	**Undetermined**	**Undetermined**	**Undetermined**
	T.trichiura	**Undetermined**	**Undetermined**	**Undetermined**	**Undetermined**
	A. lumbricoides	**Undetermined**	**Undetermined**	**Undetermined**	**Undetermined**
**130**	***N. americanus***	**Undetermined**	**Undetermined**	**Undetermined**	**Undetermined**
	T.trichiura	**Undetermined**	**Undetermined**	**Undetermined**	**Undetermined**
	A. lumbricoides	**Undetermined**	**Undetermined**	**Undetermined**	**Undetermined**

131	*N. americanus*	Undetermined	Undetermined	Undetermined	Undetermined
	T.trichiura	Undetermined	Undetermined	Undetermined	Undetermined
	A. lumbricoides	Undetermined	Undetermined	Undetermined	Undetermined
132	*N. americanus*	Undetermined	Undetermined	Undetermined	Undetermined
	T.trichiura	Undetermined	Undetermined	Undetermined	Undetermined
	A. lumbricoides	Undetermined	Undetermined	Undetermined	Undetermined
133	*N. americanus*	Undetermined	Undetermined	Undetermined	Undetermined
	T.trichiura	Undetermined	Undetermined	Undetermined	Undetermined
	A. lumbricoides	Undetermined	Undetermined	Undetermined	Undetermined
134	*N. americanus*	Undetermined	Undetermined	Undetermined	Undetermined
	T.trichiura	Undetermined	Undetermined	Undetermined	Undetermined
	A. lumbricoides	Undetermined	Undetermined	Undetermined	Undetermined
135	*N. americanus*	Undetermined	Undetermined	Undetermined	Undetermined
	T.trichiura	Undetermined	Undetermined	Undetermined	Undetermined
	A. lumbricoides	Undetermined	Undetermined	Undetermined	Undetermined
136	*N. americanus*	Undetermined	Undetermined	Undetermined	Undetermined
	T.trichiura	Undetermined	Undetermined	Undetermined	Undetermined
	A. lumbricoides	Undetermined	Undetermined	Undetermined	Undetermined
137	*N. americanus*	Undetermined	Undetermined	Undetermined	Undetermined
	T.trichiura	Undetermined	Undetermined	Undetermined	Undetermined
	A. lumbricoides	Undetermined	Undetermined	Undetermined	Undetermined
138	*N. americanus*	Undetermined	Undetermined	Undetermined	Undetermined
	T.trichiura	Undetermined	Undetermined	Undetermined	Undetermined
	A. lumbricoides	Undetermined	Undetermined	Undetermined	Undetermined
139	*N. americanus*	Undetermined	Undetermined	Undetermined	Undetermined
	T.trichiura	Undetermined	Undetermined	Undetermined	Undetermined
	A. lumbricoides	Undetermined	Undetermined	Undetermined	Undetermined
140	*N. americanus*	Undetermined	Undetermined	Undetermined	Undetermined
	T.trichiura	Undetermined	Undetermined	Undetermined	Undetermined
	A. lumbricoides	Undetermined	Undetermined	Undetermined	Undetermined
141	*N. americanus*	Undetermined	Undetermined	Undetermined	Undetermined
	T.trichiura	Undetermined	Undetermined	Undetermined	Undetermined
	A. lumbricoides	Undetermined	Undetermined	Undetermined	Undetermined
142	*N. americanus*	Undetermined	Undetermined	Undetermined	Undetermined
	T.trichiura	Undetermined	Undetermined	Undetermined	Undetermined
	A. lumbricoides	Undetermined	Undetermined	Undetermined	Undetermined
143	*N. americanus*	Undetermined	Undetermined	Undetermined	Undetermined
	T.trichiura	Undetermined	Undetermined	Undetermined	Undetermined
	A. lumbricoides	Undetermined	Undetermined	Undetermined	Undetermined

144	***N. americanus***	**Undetermined**	**Undetermined**	**Undetermined**	**Undetermined**
	T.trichiura	**Undetermined**	**Undetermined**	**Undetermined**	**Undetermined**
	A. lumbricoides	**Undetermined**	**Undetermined**	**Undetermined**	**Undetermined**
145	***N. americanus***	**Undetermined**	**Undetermined**	**Undetermined**	**Undetermined**
	T.trichiura	**Undetermined**	**Undetermined**	**Undetermined**	**Undetermined**
	A. lumbricoides	**Undetermined**	**Undetermined**	**Undetermined**	**Undetermined**
146	***N. americanus***	**Undetermined**	**Undetermined**	**Undetermined**	**Undetermined**
	T.trichiura	**Undetermined**	**Undetermined**	**Undetermined**	**Undetermined**
	A. lumbricoides	**Undetermined**	**Undetermined**	**Undetermined**	**Undetermined**
147	***N. americanus***	**Undetermined**	**Undetermined**	**Undetermined**	**Undetermined**
	T.trichiura	**Undetermined**	**Undetermined**	**Undetermined**	**Undetermined**
	A. lumbricoides	**Undetermined**	**Undetermined**	**Undetermined**	**Undetermined**
148	***N. americanus***	**Undetermined**	**Undetermined**	**Undetermined**	**Undetermined**
	T.trichiura	**Undetermined**	**Undetermined**	**Undetermined**	**Undetermined**
	A. lumbricoides	**Undetermined**	**Undetermined**	**Undetermined**	**Undetermined**
149	***N. americanus***	**Undetermined**	**Undetermined**	**Undetermined**	**Undetermined**
	T.trichiura	**Undetermined**	**Undetermined**	**Undetermined**	**Undetermined**
	A. lumbricoides	**Undetermined**	**Undetermined**	**Undetermined**	**Undetermined**
150	***N. americanus***	**Undetermined**	**Undetermined**	**Undetermined**	**Undetermined**
	T.trichiura	**Undetermined**	**Undetermined**	**Undetermined**	**Undetermined**
	A. lumbricoides	**Undetermined**	**Undetermined**	**Undetermined**	**Undetermined**
151	***N. americanus***	**Undetermined**	**Undetermined**	**Undetermined**	**Undetermined**
	T.trichiura	**Undetermined**	**Undetermined**	**Undetermined**	**Undetermined**
	A. lumbricoides	**Undetermined**	**Undetermined**	**Undetermined**	**Undetermined**
152	***N. americanus***	**Undetermined**	**Undetermined**	**Undetermined**	**Undetermined**
	T.trichiura	**Undetermined**	**Undetermined**	**Undetermined**	**Undetermined**
	A. lumbricoides	**Undetermined**	**Undetermined**	**Undetermined**	**Undetermined**
153	***N. americanus***	**Undetermined**	**Undetermined**	**Undetermined**	**Undetermined**
	T.trichiura	**Undetermined**	**Undetermined**	**Undetermined**	**Undetermined**
	A. lumbricoides	**Undetermined**	**Undetermined**	**Undetermined**	**Undetermined**
154	***N. americanus***	**Undetermined**	**Undetermined**	**Undetermined**	**Undetermined**
	T.trichiura	**Undetermined**	**Undetermined**	**Undetermined**	**Undetermined**
	A. lumbricoides	**Undetermined**	**Undetermined**	**Undetermined**	**Undetermined**
155	***N. americanus***	**Undetermined**	**Undetermined**	**Undetermined**	**Undetermined**
	T.trichiura	**Undetermined**	**Undetermined**	**Undetermined**	**Undetermined**
	A. lumbricoides	**Undetermined**	**Undetermined**	**Undetermined**	**Undetermined**
156	***N. americanus***	**Undetermined**	**Undetermined**	**Undetermined**	**Undetermined**
	T.trichiura	**Undetermined**	**Undetermined**	**Undetermined**	**Undetermined**
	A. lumbricoides	**Undetermined**	**Undetermined**	**Undetermined**	**Undetermined**

**157**	***N. americanus***	**Undetermined**	**Undetermined**	**Undetermined**	**Undetermined**
	T.trichiura	**Undetermined**	**Undetermined**	**Undetermined**	**Undetermined**
	A. lumbricoides	**Undetermined**	**Undetermined**	**Undetermined**	**Undetermined**
**158**	***N. americanus***	**Undetermined**	**Undetermined**	**Undetermined**	**Undetermined**
	T.trichiura	**Undetermined**	**Undetermined**	**Undetermined**	**Undetermined**
	A. lumbricoides	**Undetermined**	**Undetermined**	**Undetermined**	**Undetermined**
**159**	***N. americanus***	**Undetermined**	**Undetermined**	**Undetermined**	**Undetermined**
	T.trichiura	**Undetermined**	**Undetermined**	**Undetermined**	**Undetermined**
	A. lumbricoides	**Undetermined**	**Undetermined**	**Undetermined**	**Undetermined**
**160**	***N. americanus***	**Undetermined**	**Undetermined**	**Undetermined**	**Undetermined**
	T.trichiura	**Undetermined**	**Undetermined**	**Undetermined**	**Undetermined**
	A. lumbricoides	**Undetermined**	**Undetermined**	**Undetermined**	**Undetermined**
**161**	***N. americanus***	**Undetermined**	**Undetermined**	**Undetermined**	**Undetermined**
	T.trichiura	**Undetermined**	**Undetermined**	**Undetermined**	**Undetermined**
	A. lumbricoides	**Undetermined**	**Undetermined**	**Undetermined**	**Undetermined**
**162**	***N. americanus***	**Undetermined**	**Undetermined**	**Undetermined**	**Undetermined**
	T.trichiura	**Undetermined**	**Undetermined**	**Undetermined**	**Undetermined**
	A. lumbricoides	**Undetermined**	**Undetermined**	**Undetermined**	**Undetermined**
**163**	***N. americanus***	**Undetermined**	**Undetermined**	**Undetermined**	**Undetermined**
	T.trichiura	**Undetermined**	**Undetermined**	**Undetermined**	**Undetermined**
	A. lumbricoides	**Undetermined**	**Undetermined**	**Undetermined**	**Undetermined**
**164**	***N. americanus***	**Undetermined**	**Undetermined**	**Undetermined**	**Undetermined**
	T.trichiura	**Undetermined**	**Undetermined**	**Undetermined**	**Undetermined**
	A. lumbricoides	**Undetermined**	**Undetermined**	**Undetermined**	**Undetermined**
**165**	***N. americanus***	**Undetermined**	**Undetermined**	**Undetermined**	**Undetermined**
	T.trichiura	**Undetermined**	**Undetermined**	**Undetermined**	**Undetermined**
	A. lumbricoides	**Undetermined**	**Undetermined**	**Undetermined**	**Undetermined**
**166**	***N. americanus***	**Undetermined**	**Undetermined**	**Undetermined**	**Undetermined**
	T.trichiura	**Undetermined**	**Undetermined**	**Undetermined**	**Undetermined**
	A. lumbricoides	**Undetermined**	**Undetermined**	**Undetermined**	**Undetermined**
**167**	***N. americanus***	**Undetermined**	**Undetermined**	**Undetermined**	**Undetermined**
	T.trichiura	**Undetermined**	**Undetermined**	**Undetermined**	**Undetermined**
	A. lumbricoides	**Undetermined**	**Undetermined**	**Undetermined**	**Undetermined**
**168**	***N. americanus***	**Undetermined**	**Undetermined**	**Undetermined**	**Undetermined**
	T.trichiura	**Undetermined**	**Undetermined**	**Undetermined**	**Undetermined**
	A. lumbricoides	**Undetermined**	**Undetermined**	**Undetermined**	**Undetermined**
**169**	***N. americanus***	**Undetermined**	**Undetermined**	**Undetermined**	**Undetermined**
	T.trichiura	**Undetermined**	**Undetermined**	**Undetermined**	**Undetermined**
	A. lumbricoides	**Undetermined**	**Undetermined**	**Undetermined**	**Undetermined**

**170**	***N. americanus***	**Undetermined**	**Undetermined**	**Undetermined**	**Undetermined**
	T.trichiura	**Undetermined**	**Undetermined**	**Undetermined**	**Undetermined**
	A. lumbricoides	**Undetermined**	**Undetermined**	**Undetermined**	**Undetermined**
**171**	***N. americanus***	**Undetermined**	**Undetermined**	**Undetermined**	**Undetermined**
	T.trichiura	**Undetermined**	**Undetermined**	**Undetermined**	**Undetermined**
	A. lumbricoides	**Undetermined**	**Undetermined**	**Undetermined**	**Undetermined**
**172**	***N. americanus***	**Undetermined**	**Undetermined**	**Undetermined**	**Undetermined**
	T.trichiura	**Undetermined**	**Undetermined**	**Undetermined**	**Undetermined**
	A. lumbricoides	**Undetermined**	**Undetermined**	**Undetermined**	**Undetermined**
**173**	***N. americanus***	**Undetermined**	**Undetermined**	**Undetermined**	**Undetermined**
	T.trichiura	**Undetermined**	**Undetermined**	**Undetermined**	**Undetermined**
	A. lumbricoides	**Undetermined**	**Undetermined**	**Undetermined**	**Undetermined**
**174**	***N. americanus***	**Undetermined**	**Undetermined**	**Undetermined**	**Undetermined**
	T.trichiura	**Undetermined**	**Undetermined**	**Undetermined**	**Undetermined**
	A. lumbricoides	**Undetermined**	**Undetermined**	**Undetermined**	**Undetermined**
**175**	***N. americanus***	**Undetermined**	**Undetermined**	**Undetermined**	**Undetermined**
	T.trichiura	**Undetermined**	**Undetermined**	**Undetermined**	**Undetermined**
	A. lumbricoides	**Undetermined**	**Undetermined**	**Undetermined**	**Undetermined**
**176**	***N. americanus***	**Undetermined**	**Undetermined**	**Undetermined**	**Undetermined**
	T.trichiura	**Undetermined**	**Undetermined**	**Undetermined**	**Undetermined**
	A. lumbricoides	**Undetermined**	**Undetermined**	**Undetermined**	**Undetermined**
**177**	***N. americanus***	**Undetermined**	**Undetermined**	**Undetermined**	**Undetermined**
	T.trichiura	**Undetermined**	**Undetermined**	**Undetermined**	**Undetermined**
	A. lumbricoides	**Undetermined**	**Undetermined**	**Undetermined**	**Undetermined**
**178**	***N. americanus***	**Undetermined**	**Undetermined**	**Undetermined**	**Undetermined**
	T.trichiura	**Undetermined**	**Undetermined**	**Undetermined**	**Undetermined**
	A. lumbricoides	**Undetermined**	**Undetermined**	**Undetermined**	**Undetermined**
**179**	***N. americanus***	**Undetermined**	**Undetermined**	**Undetermined**	**Undetermined**
	T.trichiura	**Undetermined**	**Undetermined**	**Undetermined**	**Undetermined**
	A. lumbricoides	**Undetermined**	**Undetermined**	**Undetermined**	**Undetermined**
**180**	***N. americanus***	**Undetermined**	**Undetermined**	**Undetermined**	**Undetermined**
	T.trichiura	**Undetermined**	**Undetermined**	**Undetermined**	**Undetermined**
	A. lumbricoides	**Undetermined**	**Undetermined**	**Undetermined**	**Undetermined**
**181**	***N. americanus***	**Undetermined**	**Undetermined**	**Undetermined**	**Undetermined**
	T.trichiura	**Undetermined**	**Undetermined**	**Undetermined**	**Undetermined**
	A. lumbricoides	**Undetermined**	**Undetermined**	**Undetermined**	**Undetermined**
**182**	***N. americanus***	**Undetermined**	**Undetermined**	**Undetermined**	**Undetermined**
	T.trichiura	**Undetermined**	**Undetermined**	**Undetermined**	**Undetermined**
	A. lumbricoides	**Undetermined**	**Undetermined**	**Undetermined**	**Undetermined**

**183**	***N. americanus***	**Undetermined**	**Undetermined**	**Undetermined**	**Undetermined**
	T.trichiura	**Undetermined**	**Undetermined**	**Undetermined**	**Undetermined**
	A. lumbricoides	**Undetermined**	**Undetermined**	**Undetermined**	**Undetermined**
**184**	***N. americanus***	**Undetermined**	**Undetermined**	**Undetermined**	**Undetermined**
	T.trichiura	**Undetermined**	**Undetermined**	**Undetermined**	**Undetermined**
	A. lumbricoides	**Undetermined**	**Undetermined**	**Undetermined**	**Undetermined**
**185**	***N. americanus***	**Undetermined**	**Undetermined**	**Undetermined**	**Undetermined**
	T.trichiura	**Undetermined**	**Undetermined**	**Undetermined**	**Undetermined**
	A. lumbricoides	**Undetermined**	**Undetermined**	**Undetermined**	**Undetermined**
**186**	***N. americanus***	**Undetermined**	**Undetermined**	**Undetermined**	**Undetermined**
	T.trichiura	**Undetermined**	**Undetermined**	**Undetermined**	**Undetermined**
	A. lumbricoides	**Undetermined**	**Undetermined**	**Undetermined**	**Undetermined**
**187**	***N. americanus***	**Undetermined**	**Undetermined**	**Undetermined**	**Undetermined**
	T.trichiura	**Undetermined**	**Undetermined**	**Undetermined**	**Undetermined**
	A. lumbricoides	**Undetermined**	**Undetermined**	**Undetermined**	**Undetermined**
**188**	***N. americanus***	**Undetermined**	**Undetermined**	**Undetermined**	**Undetermined**
	T.trichiura	**Undetermined**	**Undetermined**	**Undetermined**	**Undetermined**
	A. lumbricoides	**Undetermined**	**Undetermined**	**Undetermined**	**Undetermined**
**189**	***N. americanus***	**Undetermined**	**Undetermined**	**Undetermined**	**Undetermined**
	T.trichiura	**Undetermined**	**Undetermined**	**Undetermined**	**Undetermined**
	A. lumbricoides	**Undetermined**	**Undetermined**	**Undetermined**	**Undetermined**
**190**	***N. americanus***	**Undetermined**	**Undetermined**	**Undetermined**	**Undetermined**
	T.trichiura	**Undetermined**	**Undetermined**	**Undetermined**	**Undetermined**
	A. lumbricoides	**Undetermined**	**Undetermined**	**Undetermined**	**Undetermined**
**191**	***N. americanus***	**Undetermined**	**Undetermined**	**Undetermined**	**Undetermined**
	T.trichiura	**Undetermined**	**Undetermined**	**Undetermined**	**Undetermined**
	A. lumbricoides	**Undetermined**	**Undetermined**	**Undetermined**	**Undetermined**
**192**	***N. americanus***	**Undetermined**	**Undetermined**	**Undetermined**	**Undetermined**
	T.trichiura	**Undetermined**	**Undetermined**	**Undetermined**	**Undetermined**
	A. lumbricoides	**Undetermined**	**Undetermined**	**Undetermined**	**Undetermined**
**193**	***N. americanus***	**Undetermined**	**Undetermined**	**Undetermined**	**Undetermined**
	T.trichiura	**Undetermined**	**Undetermined**	**Undetermined**	**Undetermined**
	A. lumbricoides	**Undetermined**	**Undetermined**	**Undetermined**	**Undetermined**
**194**	***N. americanus***	**Undetermined**	**Undetermined**	**Undetermined**	**Undetermined**
	T.trichiura	**Undetermined**	**Undetermined**	**Undetermined**	**Undetermined**
	A. lumbricoides	**Undetermined**	**Undetermined**	**Undetermined**	**Undetermined**
**195**	***N. americanus***	**Undetermined**	**Undetermined**	**Undetermined**	**Undetermined**
	T.trichiura	**Undetermined**	**Undetermined**	**Undetermined**	**Undetermined**
	A. lumbricoides	**Undetermined**	**Undetermined**	**Undetermined**	**Undetermined**

**196**	***N. americanus***	**Undetermined**	**Undetermined**	**Undetermined**	**Undetermined**
	T.trichiura	**Undetermined**	**Undetermined**	**Undetermined**	**Undetermined**
	A. lumbricoides	**Undetermined**	**Undetermined**	**Undetermined**	**Undetermined**
**197**	***N. americanus***	**Undetermined**	**Undetermined**	**Undetermined**	**Undetermined**
	T.trichiura	**Undetermined**	**Undetermined**	**Undetermined**	**Undetermined**
	A. lumbricoides	**Undetermined**	**Undetermined**	**Undetermined**	**Undetermined**
**198**	***N. americanus***	**Undetermined**	**Undetermined**	**Undetermined**	**Undetermined**
	T.trichiura	**Undetermined**	**Undetermined**	**Undetermined**	**Undetermined**
	A. lumbricoides	**Undetermined**	**Undetermined**	**Undetermined**	**Undetermined**
**199**	***N. americanus***	**Undetermined**	**Undetermined**	**Undetermined**	**Undetermined**
	T.trichiura	**Undetermined**	**Undetermined**	**Undetermined**	**Undetermined**
	A. lumbricoides	**Undetermined**	**Undetermined**	**Undetermined**	**Undetermined**
**200**	***N. americanus***	**Undetermined**	**Undetermined**	**Undetermined**	**Undetermined**
	T.trichiura	**Undetermined**	**Undetermined**	**Undetermined**	**Undetermined**
	A. lumbricoides	**Undetermined**	**Undetermined**	**Undetermined**	**Undetermined**

Figure A7.3: *DNA line data for N. americanus at Quantigen and Smith*

	Extract 1		Extract 2		Extract 3		Extract 4	
Sample ID	Quantigen	Smith	Quantigen	Smith	Smith	Quantigen	Smith	Quantigen
1	Undetermined	Undetermined	Undetermined	Undetermined	Undetermined	Undetermined	Undetermined	Undetermined
2	Undetermined	Undetermined	Undetermined	Undetermined	Undetermined	Undetermined	Undetermined	Undetermined
3	Undetermined	Undetermined	Undetermined	Undetermined	Undetermined	Undetermined	Undetermined	Undetermined
4	Undetermined	Undetermined	Undetermined	Undetermined	Undetermined	Undetermined	Undetermined	Undetermined
5	Undetermined	Undetermined	Undetermined	Undetermined	Undetermined	Undetermined	Undetermined	Undetermined
6	25.3	24.90	24.2	22.83	18.13	18.83	18.41	18.73
7	Undetermined	Undetermined	Undetermined	Undetermined	Undetermined	Undetermined	Undetermined	Undetermined
8	Undetermined	Undetermined	Undetermined	Undetermined	Undetermined	Undetermined	Undetermined	Undetermined
9	Undetermined	Undetermined	Undetermined	Undetermined	Undetermined	36.7	Undetermined	Undetermined
10	Undetermined	Undetermined	Undetermined	Undetermined	Undetermined	Undetermined	Undetermined	Undetermined
11	Undetermined	Undetermined	Undetermined	Undetermined	Undetermined	Undetermined	Undetermined	Undetermined
12	36.3	Undetermined	35.9	Undetermined	32.4	32.1	26.7	27.6
13	Undetermined	Undetermined	Undetermined	Undetermined	Undetermined	Undetermined	Undetermined	Undetermined
14	28.8	27.7	25.7	25.7	28.4	27.8	Undetermined	Undetermined
15	23.6	23.13	23.5	21.57	21.11	16.90	17.29	21.13
16	28.4	28.09	28.6	28.45	23.91	26.17	32.14	35.48
17	Undetermined	Undetermined	Undetermined	Undetermined	Undetermined	Undetermined	Undetermined	Undetermined
18	Undetermined	Undetermined	Undetermined	Undetermined	Undetermined	Undetermined	Undetermined	Undetermined
19	Undetermined	Undetermined	Undetermined	Undetermined	Undetermined	Undetermined	Undetermined	Undetermined
20	Undetermined	Undetermined	Undetermined	Undetermined	Undetermined	Undetermined	Undetermined	Undetermined
21	Undetermined	Undetermined	Undetermined	Undetermined	Undetermined	Undetermined	Undetermined	Undetermined
22	Undetermined	Undetermined	Undetermined	Undetermined	Undetermined	Undetermined	Undetermined	Undetermined
23	28.3	27.89	27.2	26.10	23.68	24.33	24.36	25.50
24	Undetermined	Undetermined	Undetermined	Undetermined	Undetermined	Undetermined	Undetermined	Undetermined
25	Undetermined	Undetermined	Undetermined	Undetermined	Undetermined	Undetermined	Undetermined	Undetermined

26	Undetermined	Undetermined	Undetermined	Undetermined	Undetermined	Undetermined	Undetermined	Undetermined
27	Undetermined	Undetermined	Undetermined	Undetermined	Undetermined	Undetermined	Undetermined	Undetermined
28	Undetermined	Undetermined	Undetermined	Undetermined	Undetermined	Undetermined	Undetermined	Undetermined
29	Undetermined	Undetermined	Undetermined	Undetermined	Undetermined	Undetermined	Undetermined	Undetermined
30	Undetermined	Undetermined	Undetermined	Undetermined	Undetermined	Undetermined	Undetermined	Undetermined
31	Undetermined	Undetermined	Undetermined	Undetermined	Undetermined	Undetermined	Undetermined	Undetermined
32	Undetermined	Undetermined	Undetermined	Undetermined	Undetermined	Undetermined	Undetermined	Undetermined
33	Undetermined	32.0	Undetermined	Undetermined	Undetermined	Undetermined	Undetermined	Undetermined
34	Undetermined	Undetermined	Undetermined	Undetermined	Undetermined	Undetermined	Undetermined	Undetermined
35	Undetermined	Undetermined	Undetermined	Undetermined	Undetermined	Undetermined	Undetermined	36.6
36	Undetermined	Undetermined	Undetermined	Undetermined	Undetermined	Undetermined	Undetermined	Undetermined
37	Undetermined	Undetermined	Undetermined	Undetermined	Undetermined	Undetermined	Undetermined	Undetermined
38	Undetermined	Undetermined	Undetermined	Undetermined	Undetermined	Undetermined	Undetermined	Undetermined
39	26.9	25.92	28.5	26.25	20.77	24.00	22.83	25.96
40	Undetermined	Undetermined	32.5	30.1	Undetermined	Undetermined	Undetermined	29.5
41	36.5	35.2	Undetermined	Undetermined	Undetermined	Undetermined	Undetermined	Undetermined
42	Undetermined	Undetermined	Undetermined	Undetermined	Undetermined	Undetermined	Undetermined	Undetermined
43	Undetermined	Undetermined	Undetermined	Undetermined	Undetermined	Undetermined	Undetermined	Undetermined
44	Undetermined	Undetermined	Undetermined	Undetermined	Undetermined	Undetermined	Undetermined	Undetermined
45	Undetermined	Undetermined	Undetermined	Undetermined	Undetermined	Undetermined	Undetermined	Undetermined
46	Undetermined	Undetermined	Undetermined	Undetermined	Undetermined	Undetermined	Undetermined	Undetermined
47	Undetermined	Undetermined	Undetermined	Undetermined	Undetermined	Undetermined	Undetermined	Undetermined
48	36.7	Undetermined	36.7	Undetermined	36.2	Undetermined	29.1	30.5
49	Undetermined	Undetermined	Undetermined	Undetermined	Undetermined	Undetermined	Undetermined	Undetermined
50	33.3	32.53	34.1	32.31	30.46	31.16	29.98	31.48
51	Undetermined	Undetermined	Undetermined	Undetermined	Undetermined	Undetermined	Undetermined	Undetermined
52	Undetermined	Undetermined	Undetermined	Undetermined	Undetermined	Undetermined	Undetermined	Undetermined
53	Undetermined	Undetermined	Undetermined	Undetermined	Undetermined	Undetermined	Undetermined	Undetermined
54	Undetermined	Undetermined	Undetermined	Undetermined	Undetermined	Undetermined	Undetermined	Undetermined
55	Undetermined	Undetermined	Undetermined	Undetermined	Undetermined	Undetermined	Undetermined	Undetermined

56	Undetermined	Undetermined	Undetermined	Undetermined	Undetermined	Undetermined	Undetermined	Undetermined
57	Undetermined	Undetermined	Undetermined	Undetermined	Undetermined	Undetermined	Undetermined	Undetermined
58	Undetermined	Undetermined	Undetermined	Undetermined	Undetermined	Undetermined	Undetermined	Undetermined
59	Undetermined	Undetermined	Undetermined	Undetermined	Undetermined	Undetermined	Undetermined	Undetermined
60	24.7	24.90	22.9	21.26	17.30	18.24	20.57	21.22
61	Undetermined	Undetermined	Undetermined	Undetermined	Undetermined	Undetermined	Undetermined	Undetermined
62	Undetermined	Undetermined	Undetermined	Undetermined	Undetermined	Undetermined	Undetermined	Undetermined
63	Undetermined	Undetermined	Undetermined	Undetermined	Undetermined	Undetermined	Undetermined	Undetermined
64	Undetermined	Undetermined	Undetermined	Undetermined	Undetermined	Undetermined	Undetermined	Undetermined
65	Undetermined	Undetermined	Undetermined	Undetermined	Undetermined	Undetermined	Undetermined	Undetermined
66	Undetermined	Undetermined	Undetermined	Undetermined	Undetermined	Undetermined	Undetermined	Undetermined
67	Undetermined	Undetermined	Undetermined	Undetermined	Undetermined	Undetermined	Undetermined	Undetermined
68	25.1	25.03	31.0	29.63	19.69	19.32	18.99	19.94
69	Undetermined	Undetermined	Undetermined	Undetermined	Undetermined	Undetermined	Undetermined	Undetermined
70	Undetermined	Undetermined	Undetermined	Undetermined	Undetermined	Undetermined	Undetermined	Undetermined
71	Undetermined	Undetermined	Undetermined	Undetermined	Undetermined	Undetermined	Undetermined	Undetermined
72	Undetermined	Undetermined	Undetermined	Undetermined	Undetermined	Undetermined	Undetermined	Undetermined
73	Undetermined	Undetermined	Undetermined	Undetermined	Undetermined	Undetermined	Undetermined	Undetermined
74	Undetermined	Undetermined	Undetermined	Undetermined	Undetermined	Undetermined	Undetermined	Undetermined
75	Undetermined	Undetermined	Undetermined	Undetermined	Undetermined	Undetermined	Undetermined	Undetermined
76	Undetermined	Undetermined	Undetermined	Undetermined	Undetermined	Undetermined	Undetermined	Undetermined
77	Undetermined	Undetermined	Undetermined	Undetermined	Undetermined	Undetermined	Undetermined	Undetermined
78	Undetermined	Undetermined	Undetermined	Undetermined	Undetermined	Undetermined	Undetermined	Undetermined
79	Undetermined	Undetermined	Undetermined	Undetermined	Undetermined	Undetermined	Undetermined	Undetermined
80	Undetermined	Undetermined	Undetermined	Undetermined	Undetermined	Undetermined	Undetermined	Undetermined
81	Undetermined	Undetermined	Undetermined	Undetermined	Undetermined	Undetermined	Undetermined	Undetermined
82	Undetermined	Undetermined	Undetermined	Undetermined	Undetermined	Undetermined	Undetermined	Undetermined
83	Undetermined	Undetermined	Undetermined	Undetermined	Undetermined	Undetermined	Undetermined	Undetermined
84	Undetermined	Undetermined	Undetermined	Undetermined	Undetermined	Undetermined	Undetermined	Undetermined
85	Undetermined	Undetermined	Undetermined	Undetermined	Undetermined	Undetermined	Undetermined	Undetermined

86	Undetermined	Undetermined	Undetermined	Undetermined	Undetermined	Undetermined	Undetermined	Undetermined
87	Undetermined	Undetermined	Undetermined	Undetermined	Undetermined	Undetermined	Undetermined	Undetermined
88	Undetermined	Undetermined	Undetermined	Undetermined	Undetermined	Undetermined	Undetermined	Undetermined
89	Undetermined	Undetermined	Undetermined	Undetermined	Undetermined	Undetermined	Undetermined	Undetermined
90	Undetermined	Undetermined	Undetermined	Undetermined	Undetermined	Undetermined	Undetermined	Undetermined
91	Undetermined	Undetermined	Undetermined	Undetermined	Undetermined	Undetermined	Undetermined	Undetermined
92	Undetermined	Undetermined	Undetermined	Undetermined	Undetermined	Undetermined	Undetermined	Undetermined
93	Undetermined	Undetermined	Undetermined	Undetermined	Undetermined	Undetermined	Undetermined	Undetermined
94	Undetermined	Undetermined	Undetermined	Undetermined	Undetermined	Undetermined	Undetermined	Undetermined
95	Undetermined	Undetermined	Undetermined	Undetermined	Undetermined	Undetermined	Undetermined	Undetermined
96	Undetermined	Undetermined	Undetermined	Undetermined	Undetermined	Undetermined	Undetermined	Undetermined
97	Undetermined	Undetermined	Undetermined	Undetermined	Undetermined	Undetermined	Undetermined	Undetermined
98	Undetermined	Undetermined	Undetermined	Undetermined	Undetermined	Undetermined	Undetermined	Undetermined
99	Undetermined	Undetermined	Undetermined	Undetermined	Undetermined	Undetermined	Undetermined	Undetermined
100	Undetermined	Undetermined	36.9	Undetermined	Undetermined	Undetermined	Undetermined	Undetermined
101	Undetermined	Undetermined	Undetermined	Undetermined	Undetermined	Undetermined	Undetermined	Undetermined
102	Undetermined	Undetermined	Undetermined	Undetermined	Undetermined	Undetermined	Undetermined	Undetermined
103	Undetermined	Undetermined	Undetermined	Undetermined	Undetermined	Undetermined	Undetermined	Undetermined
104	Undetermined	Undetermined	Undetermined	Undetermined	Undetermined	Undetermined	Undetermined	Undetermined
105	Undetermined	Undetermined	Undetermined	Undetermined	Undetermined	Undetermined	Undetermined	Undetermined
106	Undetermined	Undetermined	Undetermined	Undetermined	Undetermined	Undetermined	Undetermined	Undetermined
107	Undetermined	Undetermined	Undetermined	Undetermined	Undetermined	Undetermined	Undetermined	Undetermined
108	Undetermined	Undetermined	Undetermined	Undetermined	Undetermined	Undetermined	Undetermined	Undetermined
109	Undetermined	Undetermined	Undetermined	Undetermined	Undetermined	Undetermined	Undetermined	Undetermined
110	Undetermined	Undetermined	Undetermined	Undetermined	Undetermined	Undetermined	Undetermined	Undetermined
111	Undetermined	Undetermined	Undetermined	Undetermined	Undetermined	Undetermined	Undetermined	Undetermined
112	Undetermined	Undetermined	Undetermined	Undetermined	Undetermined	Undetermined	Undetermined	Undetermined
113	Undetermined	Undetermined	Undetermined	Undetermined	Undetermined	Undetermined	Undetermined	Undetermined
114	Undetermined	Undetermined	Undetermined	Undetermined	Undetermined	Undetermined	Undetermined	Undetermined
115	Undetermined	Undetermined	Undetermined	Undetermined	Undetermined	Undetermined	Undetermined	Undetermined

116	Undetermined	Undetermined	Undetermined	Undetermined	Undetermined	Undetermined	Undetermined	Undetermined
117	Undetermined	Undetermined	Undetermined	Undetermined	Undetermined	Undetermined	Undetermined	Undetermined
118	31.3	30.3	Undetermined	Undetermined	23.8	25.5	30.7	33.2
119	Undetermined	Undetermined	Undetermined	Undetermined	Undetermined	Undetermined	Undetermined	Undetermined
120	Undetermined	Undetermined	Undetermined	Undetermined	Undetermined	Undetermined	Undetermined	Undetermined
121	Undetermined	Undetermined	Undetermined	Undetermined	Undetermined	Undetermined	Undetermined	Undetermined
122	Undetermined	Undetermined	Undetermined	Undetermined	Undetermined	Undetermined	Undetermined	Undetermined
123	Undetermined	Undetermined	Undetermined	Undetermined	Undetermined	Undetermined	Undetermined	Undetermined
124	Undetermined	Undetermined	Undetermined	Undetermined	Undetermined	Undetermined	Undetermined	Undetermined
125	22.2	21.77	23.7	22.56	17.91	19.61	18.65	20.15
126	Undetermined	Undetermined	Undetermined	Undetermined	Undetermined	Undetermined	Undetermined	Undetermined
127	Undetermined	Undetermined	Undetermined	Undetermined	Undetermined	Undetermined	Undetermined	Undetermined
128	Undetermined	Undetermined	Undetermined	Undetermined	Undetermined	Undetermined	Undetermined	Undetermined
129	27.6	27.2	Undetermined	Undetermined	18.7	20.0	20.6	18.6
130	Undetermined	Undetermined	Undetermined	Undetermined	Undetermined	Undetermined	Undetermined	Undetermined
131	Undetermined	Undetermined	Undetermined	Undetermined	Undetermined	Undetermined	Undetermined	Undetermined
132	Undetermined	Undetermined	Undetermined	Undetermined	Undetermined	Undetermined	Undetermined	Undetermined
133	Undetermined	Undetermined	Undetermined	Undetermined	Undetermined	Undetermined	Undetermined	Undetermined
134	Undetermined	Undetermined	Undetermined	Undetermined	Undetermined	Undetermined	Undetermined	Undetermined
135	Undetermined	Undetermined	Undetermined	Undetermined	Undetermined	Undetermined	Undetermined	Undetermined
136	Undetermined	Undetermined	Undetermined	Undetermined	Undetermined	Undetermined	Undetermined	Undetermined
137	Undetermined	Undetermined	Undetermined	Undetermined	Undetermined	Undetermined	Undetermined	Undetermined
138	Undetermined	Undetermined	Undetermined	Undetermined	Undetermined	Undetermined	Undetermined	Undetermined
139	Undetermined	Undetermined	Undetermined	Undetermined	Undetermined	Undetermined	Undetermined	Undetermined
140	Undetermined	Undetermined	Undetermined	Undetermined	Undetermined	Undetermined	Undetermined	Undetermined
141	Undetermined	Undetermined	Undetermined	Undetermined	Undetermined	Undetermined	Undetermined	Undetermined
142	Undetermined	Undetermined	Undetermined	Undetermined	Undetermined	Undetermined	Undetermined	Undetermined
143	Undetermined	Undetermined	Undetermined	Undetermined	Undetermined	Undetermined	Undetermined	Undetermined
144	Undetermined	Undetermined	Undetermined	Undetermined	Undetermined	Undetermined	Undetermined	Undetermined
145	Undetermined	Undetermined	Undetermined	Undetermined	Undetermined	Undetermined	Undetermined	Undetermined

146	Undetermined	Undetermined	Undetermined	Undetermined	Undetermined	Undetermined	Undetermined	Undetermined
147	Undetermined	Undetermined	Undetermined	Undetermined	Undetermined	Undetermined	Undetermined	Undetermined
148	Undetermined	Undetermined	Undetermined	Undetermined	33.2	Undetermined	Undetermined	Undetermined
149	Undetermined	Undetermined	Undetermined	Undetermined	Undetermined	Undetermined	Undetermined	Undetermined
150	Undetermined	Undetermined	Undetermined	Undetermined	Undetermined	Undetermined	Undetermined	Undetermined
151	Undetermined	Undetermined	Undetermined	Undetermined	Undetermined	Undetermined	Undetermined	Undetermined
152	Undetermined	Undetermined	Undetermined	Undetermined	Undetermined	Undetermined	Undetermined	Undetermined
153	26.2	28.80	26.1	23.87	18.93	19.29	19.07	19.22
154	Undetermined	Undetermined	Undetermined	Undetermined	Undetermined	Undetermined	Undetermined	Undetermined
155	Undetermined	Undetermined	Undetermined	Undetermined	Undetermined	Undetermined	Undetermined	Undetermined
156	Undetermined	Undetermined	Undetermined	Undetermined	Undetermined	Undetermined	Undetermined	Undetermined
157	Undetermined	Undetermined	Undetermined	Undetermined	Undetermined	Undetermined	Undetermined	Undetermined
158	Undetermined	Undetermined	Undetermined	Undetermined	Undetermined	Undetermined	Undetermined	Undetermined
159	Undetermined	Undetermined	Undetermined	Undetermined	Undetermined	Undetermined	Undetermined	Undetermined
160	Undetermined	Undetermined	Undetermined	Undetermined	Undetermined	Undetermined	Undetermined	Undetermined
161	Undetermined	Undetermined	Undetermined	Undetermined	Undetermined	Undetermined	Undetermined	Undetermined
162	Undetermined	Undetermined	Undetermined	Undetermined	Undetermined	Undetermined	Undetermined	Undetermined
163	Undetermined	Undetermined	Undetermined	Undetermined	Undetermined	Undetermined	Undetermined	Undetermined
164	Undetermined	Undetermined	Undetermined	Undetermined	Undetermined	Undetermined	Undetermined	Undetermined
165	Undetermined	Undetermined	Undetermined	Undetermined	Undetermined	Undetermined	Undetermined	Undetermined
166	Undetermined	Undetermined	Undetermined	Undetermined	Undetermined	Undetermined	Undetermined	Undetermined
167	23.4	23.87	25.9	22.86	16.65	18.19	17.80	17.34
168	22.8	22.13	23.7	22.72	17.79	18.63	17.40	18.13
169	18.5	17.41	17.8	17.71	13.89	14.58	14.34	14.95
170	Undetermined	Undetermined	Undetermined	Undetermined	Undetermined	Undetermined	Undetermined	Undetermined
171	Undetermined	Undetermined	Undetermined	Undetermined	Undetermined	Undetermined	Undetermined	Undetermined
172	Undetermined	Undetermined	Undetermined	Undetermined	Undetermined	Undetermined	Undetermined	Undetermined
173	Undetermined	Undetermined	Undetermined	Undetermined	Undetermined	Undetermined	Undetermined	Undetermined
174	Undetermined	Undetermined	Undetermined	Undetermined	Undetermined	Undetermined	Undetermined	Undetermined
175	Undetermined	Undetermined	Undetermined	Undetermined	Undetermined	Undetermined	Undetermined	Undetermined

176	27.4	27.29	25.6	25.16	23.55	24.70	27.21	28.20
177	Undetermined	Undetermined	Undetermined	Undetermined	Undetermined	Undetermined	Undetermined	Undetermined
178	Undetermined	Undetermined	Undetermined	Undetermined	Undetermined	Undetermined	Undetermined	Undetermined
179	Undetermined	Undetermined	Undetermined	Undetermined	Undetermined	Undetermined	Undetermined	Undetermined
180	Undetermined	Undetermined	Undetermined	Undetermined	Undetermined	Undetermined	Undetermined	Undetermined
181	Undetermined	Undetermined	Undetermined	Undetermined	Undetermined	Undetermined	Undetermined	Undetermined
182	Undetermined	Undetermined	Undetermined	Undetermined	Undetermined	Undetermined	Undetermined	Undetermined
183	Undetermined	Undetermined	Undetermined	Undetermined	Undetermined	Undetermined	Undetermined	Undetermined
184	Undetermined	Undetermined	Undetermined	Undetermined	Undetermined	Undetermined	Undetermined	Undetermined
185	Undetermined	Undetermined	Undetermined	Undetermined	Undetermined	Undetermined	Undetermined	Undetermined
186	Undetermined	Undetermined	Undetermined	Undetermined	Undetermined	Undetermined	Undetermined	Undetermined
187	Undetermined	Undetermined	Undetermined	Undetermined	Undetermined	Undetermined	Undetermined	Undetermined
188	Undetermined	Undetermined	Undetermined	Undetermined	Undetermined	Undetermined	Undetermined	Undetermined
189	Undetermined	Undetermined	Undetermined	Undetermined	Undetermined	Undetermined	Undetermined	Undetermined
190	Undetermined	Undetermined	Undetermined	Undetermined	Undetermined	Undetermined	Undetermined	Undetermined
191	Undetermined	Undetermined	36.9	Undetermined	Undetermined	Undetermined	Undetermined	Undetermined
192	22.5	22.84	27.1	23.91	20.49	21.65	17.30	18.89
193	Undetermined	Undetermined	Undetermined	Undetermined	Undetermined	Undetermined	Undetermined	Undetermined
194	Undetermined	Undetermined	Undetermined	Undetermined	Undetermined	Undetermined	Undetermined	Undetermined
195	Undetermined	Undetermined	Undetermined	Undetermined	Undetermined	Undetermined	Undetermined	Undetermined
196	Undetermined	Undetermined	Undetermined	Undetermined	Undetermined	Undetermined	Undetermined	Undetermined
197	Undetermined	Undetermined	Undetermined	Undetermined	Undetermined	Undetermined	Undetermined	Undetermined
198	Undetermined	Undetermined	Undetermined	Undetermined	Undetermined	Undetermined	Undetermined	Undetermined
199	Undetermined	Undetermined	Undetermined	Undetermined	Undetermined	Undetermined	Undetermined	Undetermined
200	Undetermined	Undetermined	Undetermined	Undetermined	Undetermined	Undetermined	Undetermined	Undetermined

Figure A7.4: *DNA line data for T. trichiura at Quantigen and Smith*

	Extract 1		Extract 2		Extract 3		Extract 4	
**Sample ID**	**Quantigen**	**Smith**	**Quantigen**	**Smith**	**Smith**	**Quantigen**	**Smith**	**Quantigen**
1	Undetermined	Undetermined	Undetermined	Undetermined	Undetermined	Undetermined	Undetermined	Undetermined
2	Undetermined	Undetermined	Undetermined	Undetermined	Undetermined	Undetermined	Undetermined	Undetermined
3	Undetermined	Undetermined	Undetermined	Undetermined	Undetermined	Undetermined	Undetermined	Undetermined
4	Undetermined	Undetermined	Undetermined	Undetermined	Undetermined	Undetermined	Undetermined	Undetermined
5	Undetermined	Undetermined	Undetermined	Undetermined	Undetermined	Undetermined	Undetermined	Undetermined
6	Undetermined	Undetermined	Undetermined	Undetermined	Undetermined	Undetermined	Undetermined	Undetermined
7	Undetermined	Undetermined	Undetermined	Undetermined	Undetermined	Undetermined	Undetermined	Undetermined
8	Undetermined	Undetermined	Undetermined	Undetermined	Undetermined	Undetermined	Undetermined	Undetermined
9	Undetermined	Undetermined	Undetermined	Undetermined	Undetermined	Undetermined	Undetermined	Undetermined
10	Undetermined	Undetermined	Undetermined	Undetermined	Undetermined	Undetermined	Undetermined	Undetermined
11	Undetermined	Undetermined	Undetermined	Undetermined	Undetermined	Undetermined	Undetermined	Undetermined
12	Undetermined	Undetermined	Undetermined	Undetermined	Undetermined	Undetermined	Undetermined	Undetermined
13	Undetermined	Undetermined	Undetermined	Undetermined	Undetermined	Undetermined	Undetermined	Undetermined
14	Undetermined	Undetermined	Undetermined	Undetermined	Undetermined	Undetermined	Undetermined	Undetermined
15	Undetermined	Undetermined	Undetermined	Undetermined	Undetermined	Undetermined	Undetermined	Undetermined
16	Undetermined	Undetermined	Undetermined	Undetermined	Undetermined	Undetermined	Undetermined	Undetermined
17	Undetermined	Undetermined	Undetermined	Undetermined	Undetermined	Undetermined	Undetermined	Undetermined
18	Undetermined	Undetermined	Undetermined	Undetermined	Undetermined	Undetermined	Undetermined	Undetermined
19	Undetermined	Undetermined	Undetermined	Undetermined	Undetermined	Undetermined	Undetermined	Undetermined
20	Undetermined	Undetermined	Undetermined	Undetermined	Undetermined	Undetermined	Undetermined	Undetermined
21	Undetermined	Undetermined	Undetermined	Undetermined	Undetermined	Undetermined	Undetermined	Undetermined
22	Undetermined	Undetermined	Undetermined	Undetermined	Undetermined	Undetermined	Undetermined	Undetermined
23	Undetermined	Undetermined	Undetermined	Undetermined	Undetermined	Undetermined	Undetermined	Undetermined
24	Undetermined	Undetermined	Undetermined	Undetermined	Undetermined	Undetermined	Undetermined	Undetermined
25	Undetermined	Undetermined	Undetermined	Undetermined	Undetermined	Undetermined	Undetermined	Undetermined

26	Undetermined	Undetermined	Undetermined	Undetermined	Undetermined	Undetermined	Undetermined	Undetermined
27	Undetermined	Undetermined	Undetermined	Undetermined	Undetermined	Undetermined	Undetermined	Undetermined
28	Undetermined	Undetermined	Undetermined	Undetermined	Undetermined	Undetermined	Undetermined	Undetermined
29	Undetermined	Undetermined	Undetermined	Undetermined	Undetermined	Undetermined	Undetermined	Undetermined
30	Undetermined	Undetermined	Undetermined	Undetermined	Undetermined	Undetermined	Undetermined	Undetermined
31	Undetermined	Undetermined	Undetermined	Undetermined	Undetermined	Undetermined	Undetermined	Undetermined
32	Undetermined	Undetermined	Undetermined	Undetermined	Undetermined	Undetermined	Undetermined	Undetermined
33	Undetermined	Undetermined	Undetermined	Undetermined	Undetermined	Undetermined	Undetermined	Undetermined
34	Undetermined	Undetermined	Undetermined	Undetermined	Undetermined	Undetermined	Undetermined	Undetermined
35	Undetermined	Undetermined	Undetermined	Undetermined	Undetermined	Undetermined	Undetermined	Undetermined
36	Undetermined	Undetermined	Undetermined	Undetermined	Undetermined	Undetermined	Undetermined	Undetermined
37	Undetermined	Undetermined	Undetermined	Undetermined	Undetermined	Undetermined	Undetermined	Undetermined
38	Undetermined	Undetermined	Undetermined	Undetermined	Undetermined	Undetermined	Undetermined	Undetermined
39	Undetermined	Undetermined	Undetermined	Undetermined	Undetermined	Undetermined	Undetermined	Undetermined
40	Undetermined	Undetermined	Undetermined	Undetermined	Undetermined	Undetermined	Undetermined	Undetermined
41	Undetermined	Undetermined	Undetermined	Undetermined	Undetermined	Undetermined	Undetermined	Undetermined
42	Undetermined	Undetermined	Undetermined	Undetermined	Undetermined	Undetermined	Undetermined	Undetermined
43	Undetermined	Undetermined	Undetermined	Undetermined	Undetermined	Undetermined	Undetermined	Undetermined
44	Undetermined	Undetermined	Undetermined	Undetermined	Undetermined	Undetermined	Undetermined	Undetermined
45	Undetermined	Undetermined	Undetermined	Undetermined	Undetermined	Undetermined	Undetermined	Undetermined
46	Undetermined	Undetermined	Undetermined	Undetermined	Undetermined	Undetermined	Undetermined	Undetermined
47	Undetermined	Undetermined	Undetermined	Undetermined	Undetermined	Undetermined	Undetermined	Undetermined
48	Undetermined	Undetermined	Undetermined	Undetermined	Undetermined	Undetermined	Undetermined	Undetermined
49	Undetermined	Undetermined	Undetermined	Undetermined	Undetermined	Undetermined	Undetermined	Undetermined
50	Undetermined	Undetermined	Undetermined	Undetermined	Undetermined	Undetermined	Undetermined	Undetermined
51	Undetermined	Undetermined	Undetermined	Undetermined	Undetermined	Undetermined	Undetermined	Undetermined
52	Undetermined	Undetermined	Undetermined	Undetermined	Undetermined	Undetermined	Undetermined	Undetermined
53	Undetermined	Undetermined	Undetermined	Undetermined	Undetermined	Undetermined	Undetermined	Undetermined
54	Undetermined	Undetermined	Undetermined	Undetermined	Undetermined	Undetermined	Undetermined	Undetermined
55	Undetermined	Undetermined	Undetermined	Undetermined	Undetermined	Undetermined	Undetermined	Undetermined

56	Undetermined	Undetermined	Undetermined	Undetermined	Undetermined	Undetermined	Undetermined	Undetermined
57	Undetermined	Undetermined	Undetermined	Undetermined	Undetermined	Undetermined	Undetermined	Undetermined
58	Undetermined	Undetermined	Undetermined	Undetermined	Undetermined	Undetermined	Undetermined	Undetermined
59	Undetermined	Undetermined	Undetermined	Undetermined	Undetermined	Undetermined	Undetermined	Undetermined
60	Undetermined	Undetermined	Undetermined	Undetermined	Undetermined	Undetermined	Undetermined	Undetermined
61	Undetermined	Undetermined	Undetermined	Undetermined	Undetermined	Undetermined	Undetermined	Undetermined
62	Undetermined	Undetermined	Undetermined	Undetermined	Undetermined	Undetermined	Undetermined	Undetermined
63	Undetermined	Undetermined	Undetermined	Undetermined	Undetermined	Undetermined	Undetermined	Undetermined
64	Undetermined	Undetermined	Undetermined	Undetermined	Undetermined	Undetermined	Undetermined	Undetermined
65	Undetermined	Undetermined	Undetermined	Undetermined	Undetermined	Undetermined	Undetermined	Undetermined
66	Undetermined	Undetermined	Undetermined	Undetermined	Undetermined	Undetermined	Undetermined	Undetermined
67	Undetermined	Undetermined	Undetermined	Undetermined	Undetermined	Undetermined	Undetermined	Undetermined
68	Undetermined	Undetermined	Undetermined	Undetermined	Undetermined	Undetermined	Undetermined	Undetermined
69	Undetermined	Undetermined	Undetermined	Undetermined	Undetermined	Undetermined	Undetermined	Undetermined
70	Undetermined	Undetermined	Undetermined	Undetermined	Undetermined	Undetermined	Undetermined	Undetermined
71	Undetermined	Undetermined	Undetermined	Undetermined	Undetermined	Undetermined	Undetermined	Undetermined
72	Undetermined	Undetermined	Undetermined	Undetermined	Undetermined	Undetermined	Undetermined	Undetermined
73	Undetermined	Undetermined	Undetermined	Undetermined	Undetermined	Undetermined	Undetermined	Undetermined
74	Undetermined	Undetermined	Undetermined	Undetermined	Undetermined	Undetermined	Undetermined	Undetermined
75	Undetermined	Undetermined	Undetermined	Undetermined	Undetermined	Undetermined	Undetermined	Undetermined
76	Undetermined	Undetermined	Undetermined	Undetermined	Undetermined	Undetermined	Undetermined	Undetermined
77	Undetermined	Undetermined	Undetermined	Undetermined	Undetermined	Undetermined	Undetermined	Undetermined
78	Undetermined	Undetermined	Undetermined	Undetermined	Undetermined	Undetermined	Undetermined	Undetermined
79	Undetermined	Undetermined	Undetermined	Undetermined	Undetermined	Undetermined	Undetermined	Undetermined
80	Undetermined	Undetermined	Undetermined	Undetermined	Undetermined	Undetermined	Undetermined	Undetermined
81	Undetermined	Undetermined	Undetermined	Undetermined	Undetermined	Undetermined	Undetermined	Undetermined
82	Undetermined	Undetermined	Undetermined	Undetermined	Undetermined	Undetermined	Undetermined	Undetermined
83	Undetermined	Undetermined	Undetermined	Undetermined	Undetermined	Undetermined	Undetermined	Undetermined
84	Undetermined	Undetermined	Undetermined	Undetermined	Undetermined	Undetermined	Undetermined	Undetermined
85	Undetermined	Undetermined	Undetermined	Undetermined	Undetermined	Undetermined	Undetermined	Undetermined

86	Undetermined	Undetermined	Undetermined	Undetermined	Undetermined	Undetermined	Undetermined	Undetermined
87	Undetermined	Undetermined	Undetermined	Undetermined	Undetermined	Undetermined	Undetermined	Undetermined
88	Undetermined	Undetermined	Undetermined	Undetermined	Undetermined	Undetermined	Undetermined	Undetermined
89	Undetermined	Undetermined	Undetermined	Undetermined	Undetermined	Undetermined	Undetermined	Undetermined
90	Undetermined	Undetermined	Undetermined	Undetermined	Undetermined	Undetermined	Undetermined	Undetermined
91	Undetermined	Undetermined	Undetermined	Undetermined	Undetermined	Undetermined	Undetermined	Undetermined
92	Undetermined	Undetermined	Undetermined	Undetermined	Undetermined	Undetermined	Undetermined	Undetermined
93	Undetermined	Undetermined	Undetermined	Undetermined	Undetermined	Undetermined	Undetermined	Undetermined
94	Undetermined	Undetermined	Undetermined	Undetermined	Undetermined	Undetermined	Undetermined	Undetermined
95	Undetermined	Undetermined	Undetermined	Undetermined	Undetermined	Undetermined	Undetermined	Undetermined
96	Undetermined	Undetermined	Undetermined	Undetermined	Undetermined	Undetermined	Undetermined	Undetermined
97	Undetermined	Undetermined	Undetermined	Undetermined	Undetermined	Undetermined	Undetermined	Undetermined
98	Undetermined	Undetermined	Undetermined	Undetermined	Undetermined	Undetermined	Undetermined	Undetermined
99	Undetermined	Undetermined	Undetermined	Undetermined	Undetermined	Undetermined	Undetermined	Undetermined
100	Undetermined	Undetermined	Undetermined	Undetermined	Undetermined	Undetermined	Undetermined	Undetermined
101	Undetermined	Undetermined	Undetermined	Undetermined	Undetermined	Undetermined	Undetermined	Undetermined
102	Undetermined	Undetermined	Undetermined	Undetermined	Undetermined	Undetermined	Undetermined	Undetermined
103	Undetermined	Undetermined	Undetermined	Undetermined	Undetermined	Undetermined	Undetermined	Undetermined
104	Undetermined	Undetermined	Undetermined	Undetermined	28.6	31.3	26.8	28.1
105	Undetermined	Undetermined	Undetermined	Undetermined	Undetermined	Undetermined	Undetermined	Undetermined
106	Undetermined	Undetermined	Undetermined	Undetermined	Undetermined	Undetermined	Undetermined	Undetermined
107	Undetermined	Undetermined	Undetermined	Undetermined	Undetermined	Undetermined	Undetermined	Undetermined
108	Undetermined	Undetermined	Undetermined	Undetermined	Undetermined	Undetermined	Undetermined	Undetermined
109	Undetermined	Undetermined	Undetermined	Undetermined	Undetermined	Undetermined	Undetermined	Undetermined
110	Undetermined	Undetermined	Undetermined	Undetermined	Undetermined	Undetermined	Undetermined	Undetermined
111	Undetermined	Undetermined	Undetermined	Undetermined	Undetermined	Undetermined	Undetermined	Undetermined
112	Undetermined	Undetermined	Undetermined	Undetermined	Undetermined	Undetermined	Undetermined	Undetermined
113	Undetermined	Undetermined	Undetermined	Undetermined	Undetermined	Undetermined	Undetermined	Undetermined
114	Undetermined	Undetermined	Undetermined	Undetermined	Undetermined	Undetermined	Undetermined	Undetermined
115	Undetermined	Undetermined	Undetermined	Undetermined	Undetermined	Undetermined	Undetermined	Undetermined

116	Undetermined	Undetermined	Undetermined	Undetermined	Undetermined	Undetermined	Undetermined	Undetermined
117	Undetermined	Undetermined	Undetermined	Undetermined	Undetermined	Undetermined	Undetermined	Undetermined
118	Undetermined	Undetermined	Undetermined	Undetermined	Undetermined	Undetermined	Undetermined	Undetermined
119	Undetermined	Undetermined	Undetermined	Undetermined	Undetermined	Undetermined	Undetermined	Undetermined
120	Undetermined	Undetermined	Undetermined	Undetermined	Undetermined	Undetermined	Undetermined	Undetermined
121	Undetermined	Undetermined	Undetermined	Undetermined	Undetermined	Undetermined	Undetermined	Undetermined
122	Undetermined	Undetermined	Undetermined	Undetermined	Undetermined	Undetermined	Undetermined	Undetermined
123	Undetermined	Undetermined	Undetermined	Undetermined	Undetermined	Undetermined	Undetermined	Undetermined
124	Undetermined	Undetermined	Undetermined	Undetermined	Undetermined	Undetermined	Undetermined	Undetermined
125	Undetermined	Undetermined	Undetermined	Undetermined	Undetermined	Undetermined	Undetermined	Undetermined
126	Undetermined	Undetermined	Undetermined	Undetermined	Undetermined	Undetermined	Undetermined	Undetermined
127	Undetermined	Undetermined	Undetermined	Undetermined	Undetermined	Undetermined	Undetermined	Undetermined
128	Undetermined	Undetermined	Undetermined	Undetermined	Undetermined	Undetermined	Undetermined	Undetermined
129	Undetermined	Undetermined	Undetermined	Undetermined	Undetermined	Undetermined	Undetermined	Undetermined
130	Undetermined	Undetermined	Undetermined	Undetermined	Undetermined	Undetermined	Undetermined	Undetermined
131	Undetermined	Undetermined	Undetermined	Undetermined	Undetermined	Undetermined	Undetermined	Undetermined
132	Undetermined	Undetermined	Undetermined	Undetermined	Undetermined	Undetermined	Undetermined	Undetermined
133	Undetermined	Undetermined	Undetermined	Undetermined	Undetermined	Undetermined	Undetermined	Undetermined
134	Undetermined	Undetermined	Undetermined	Undetermined	Undetermined	Undetermined	Undetermined	Undetermined
135	Undetermined	Undetermined	Undetermined	Undetermined	Undetermined	Undetermined	Undetermined	Undetermined
136	Undetermined	Undetermined	Undetermined	Undetermined	Undetermined	Undetermined	Undetermined	Undetermined
137	Undetermined	Undetermined	Undetermined	Undetermined	Undetermined	Undetermined	Undetermined	Undetermined
138	Undetermined	Undetermined	Undetermined	Undetermined	Undetermined	Undetermined	Undetermined	Undetermined
139	Undetermined	Undetermined	Undetermined	Undetermined	Undetermined	Undetermined	Undetermined	Undetermined
140	Undetermined	Undetermined	Undetermined	Undetermined	Undetermined	Undetermined	Undetermined	Undetermined
141	Undetermined	Undetermined	Undetermined	Undetermined	Undetermined	Undetermined	Undetermined	Undetermined
142	Undetermined	Undetermined	Undetermined	Undetermined	Undetermined	Undetermined	Undetermined	Undetermined
143	Undetermined	Undetermined	Undetermined	Undetermined	Undetermined	Undetermined	Undetermined	Undetermined
144	Undetermined	Undetermined	Undetermined	Undetermined	Undetermined	Undetermined	Undetermined	Undetermined
145	Undetermined	Undetermined	Undetermined	Undetermined	Undetermined	Undetermined	Undetermined	Undetermined

146	Undetermined	Undetermined	Undetermined	Undetermined	Undetermined	Undetermined	Undetermined	Undetermined
147	Undetermined	Undetermined	Undetermined	Undetermined	Undetermined	Undetermined	Undetermined	Undetermined
148	Undetermined	Undetermined	Undetermined	Undetermined	Undetermined	Undetermined	Undetermined	Undetermined
149	Undetermined	Undetermined	Undetermined	Undetermined	Undetermined	Undetermined	Undetermined	Undetermined
150	Undetermined	Undetermined	Undetermined	Undetermined	Undetermined	Undetermined	Undetermined	Undetermined
151	Undetermined	Undetermined	Undetermined	Undetermined	Undetermined	Undetermined	Undetermined	Undetermined
152	Undetermined	Undetermined	Undetermined	Undetermined	Undetermined	Undetermined	Undetermined	Undetermined
153	Undetermined	Undetermined	Undetermined	Undetermined	Undetermined	Undetermined	Undetermined	Undetermined
154	Undetermined	Undetermined	Undetermined	Undetermined	Undetermined	Undetermined	Undetermined	Undetermined
155	Undetermined	Undetermined	Undetermined	Undetermined	Undetermined	Undetermined	Undetermined	Undetermined
156	Undetermined	Undetermined	Undetermined	Undetermined	Undetermined	Undetermined	Undetermined	Undetermined
157	Undetermined	Undetermined	Undetermined	Undetermined	Undetermined	Undetermined	Undetermined	Undetermined
158	Undetermined	Undetermined	Undetermined	Undetermined	Undetermined	Undetermined	Undetermined	Undetermined
159	Undetermined	Undetermined	Undetermined	Undetermined	Undetermined	Undetermined	Undetermined	Undetermined
160	Undetermined	Undetermined	Undetermined	Undetermined	Undetermined	Undetermined	Undetermined	Undetermined
161	Undetermined	Undetermined	Undetermined	Undetermined	Undetermined	Undetermined	Undetermined	Undetermined
162	Undetermined	Undetermined	Undetermined	Undetermined	Undetermined	Undetermined	Undetermined	Undetermined
163	Undetermined	Undetermined	Undetermined	Undetermined	Undetermined	Undetermined	Undetermined	Undetermined
164	Undetermined	Undetermined	Undetermined	Undetermined	Undetermined	Undetermined	Undetermined	Undetermined
165	Undetermined	Undetermined	Undetermined	Undetermined	Undetermined	Undetermined	Undetermined	Undetermined
166	Undetermined	Undetermined	Undetermined	Undetermined	Undetermined	Undetermined	Undetermined	Undetermined
167	Undetermined	Undetermined	Undetermined	Undetermined	28.7	30.3	29.4	30.5
168	Undetermined	Undetermined	Undetermined	Undetermined	Undetermined	Undetermined	Undetermined	Undetermined
169	Undetermined	Undetermined	Undetermined	Undetermined	Undetermined	Undetermined	Undetermined	Undetermined
170	Undetermined	Undetermined	Undetermined	Undetermined	Undetermined	Undetermined	Undetermined	Undetermined
171	Undetermined	Undetermined	Undetermined	Undetermined	Undetermined	Undetermined	Undetermined	Undetermined
172	Undetermined	Undetermined	Undetermined	Undetermined	Undetermined	Undetermined	Undetermined	Undetermined
173	Undetermined	Undetermined	Undetermined	Undetermined	Undetermined	Undetermined	Undetermined	Undetermined
174	37.1	32.8	34.1	38.1	Undetermined	Undetermined	32.3	35.7
175	Undetermined	Undetermined	Undetermined	Undetermined	Undetermined	Undetermined	Undetermined	Undetermined

176	Undetermined	Undetermined	Undetermined	Undetermined	Undetermined	Undetermined	Undetermined	Undetermined
177	Undetermined	Undetermined	Undetermined	Undetermined	Undetermined	Undetermined	Undetermined	Undetermined
178	Undetermined	Undetermined	Undetermined	Undetermined	Undetermined	Undetermined	Undetermined	Undetermined
179	Undetermined	Undetermined	Undetermined	Undetermined	Undetermined	Undetermined	Undetermined	Undetermined
180	Undetermined	Undetermined	Undetermined	Undetermined	Undetermined	Undetermined	Undetermined	Undetermined
181	Undetermined	Undetermined	Undetermined	Undetermined	Undetermined	Undetermined	Undetermined	Undetermined
182	Undetermined	Undetermined	Undetermined	Undetermined	Undetermined	Undetermined	Undetermined	Undetermined
183	Undetermined	Undetermined	Undetermined	Undetermined	Undetermined	Undetermined	Undetermined	Undetermined
184	Undetermined	Undetermined	Undetermined	Undetermined	Undetermined	Undetermined	Undetermined	Undetermined
185	Undetermined	Undetermined	Undetermined	Undetermined	Undetermined	Undetermined	Undetermined	Undetermined
186	Undetermined	Undetermined	Undetermined	Undetermined	Undetermined	Undetermined	Undetermined	Undetermined
187	Undetermined	Undetermined	Undetermined	Undetermined	Undetermined	Undetermined	Undetermined	Undetermined
188	Undetermined	Undetermined	Undetermined	Undetermined	Undetermined	Undetermined	Undetermined	Undetermined
189	Undetermined	Undetermined	Undetermined	Undetermined	Undetermined	Undetermined	Undetermined	Undetermined
190	Undetermined	Undetermined	Undetermined	Undetermined	Undetermined	Undetermined	Undetermined	Undetermined
191	Undetermined	Undetermined	Undetermined	Undetermined	Undetermined	Undetermined	Undetermined	Undetermined
192	Undetermined	Undetermined	Undetermined	Undetermined	Undetermined	Undetermined	Undetermined	Undetermined
193	Undetermined	Undetermined	Undetermined	Undetermined	Undetermined	Undetermined	Undetermined	Undetermined
194	Undetermined	Undetermined	Undetermined	Undetermined	Undetermined	Undetermined	Undetermined	Undetermined
195	Undetermined	Undetermined	Undetermined	Undetermined	Undetermined	Undetermined	Undetermined	Undetermined
196	Undetermined	Undetermined	Undetermined	Undetermined	Undetermined	Undetermined	Undetermined	Undetermined
197	Undetermined	Undetermined	Undetermined	Undetermined	Undetermined	Undetermined	Undetermined	Undetermined
198	Undetermined	Undetermined	Undetermined	Undetermined	Undetermined	Undetermined	Undetermined	Undetermined
199	Undetermined	Undetermined	Undetermined	Undetermined	Undetermined	Undetermined	Undetermined	Undetermined
200	Undetermined	Undetermined	Undetermined	Undetermined	Undetermined	Undetermined	Undetermined	Undetermined

Figure A7.5: *DNA line data for A. lumbricoides at Quantigen and Smith*

	Extract 1		Extract 2		Extract 3		Extract 4	
**Sample ID**	**Quantigen**	**Smith**	**Quantigen**	**Smith**	**Smith**	**Quantigen**	**Smith**	**Quantigen**
1	Undetermined	Undetermined	Undetermined	Undetermined	Undetermined	Undetermined	Undetermined	Undetermined
2	Undetermined	Undetermined	Undetermined	Undetermined	Undetermined	Undetermined	Undetermined	Undetermined
3	Undetermined	Undetermined	Undetermined	Undetermined	Undetermined	Undetermined	Undetermined	Undetermined
4	Undetermined	Undetermined	Undetermined	Undetermined	Undetermined	Undetermined	Undetermined	Undetermined
5	Undetermined	Undetermined	Undetermined	Undetermined	Undetermined	Undetermined	Undetermined	Undetermined
6	Undetermined	Undetermined	Undetermined	Undetermined	Undetermined	Undetermined	Undetermined	Undetermined
7	Undetermined	Undetermined	Undetermined	Undetermined	Undetermined	Undetermined	Undetermined	Undetermined
8	Undetermined	Undetermined	36.2	Undetermined	Undetermined	Undetermined	Undetermined	Undetermined
9	Undetermined	Undetermined	Undetermined	Undetermined	Undetermined	Undetermined	Undetermined	Undetermined
10	Undetermined	Undetermined	Undetermined	Undetermined	Undetermined	Undetermined	Undetermined	Undetermined
11	Undetermined	Undetermined	Undetermined	Undetermined	Undetermined	Undetermined	Undetermined	Undetermined
12	Undetermined	Undetermined	Undetermined	Undetermined	Undetermined	Undetermined	Undetermined	Undetermined
13	Undetermined	Undetermined	Undetermined	Undetermined	Undetermined	Undetermined	Undetermined	Undetermined
14	Undetermined	Undetermined	Undetermined	Undetermined	Undetermined	Undetermined	Undetermined	Undetermined
15	Undetermined	Undetermined	Undetermined	Undetermined	Undetermined	Undetermined	Undetermined	Undetermined
16	Undetermined	Undetermined	Undetermined	Undetermined	Undetermined	Undetermined	Undetermined	Undetermined
17	Undetermined	Undetermined	Undetermined	Undetermined	Undetermined	Undetermined	Undetermined	Undetermined
18	Undetermined	Undetermined	Undetermined	Undetermined	Undetermined	Undetermined	Undetermined	Undetermined
19	Undetermined	Undetermined	Undetermined	Undetermined	Undetermined	Undetermined	Undetermined	Undetermined
20	Undetermined	Undetermined	Undetermined	Undetermined	Undetermined	Undetermined	Undetermined	Undetermined
21	Undetermined	Undetermined	Undetermined	Undetermined	Undetermined	Undetermined	Undetermined	Undetermined
22	Undetermined	Undetermined	Undetermined	Undetermined	Undetermined	Undetermined	Undetermined	Undetermined
23	Undetermined	Undetermined	Undetermined	Undetermined	Undetermined	Undetermined	Undetermined	Undetermined
24	Undetermined	Undetermined	Undetermined	Undetermined	Undetermined	Undetermined	Undetermined	Undetermined
25	Undetermined	Undetermined	Undetermined	Undetermined	Undetermined	Undetermined	Undetermined	Undetermined

26	Undetermined	Undetermined	Undetermined	Undetermined	Undetermined	Undetermined	Undetermined	Undetermined
27	Undetermined	Undetermined	Undetermined	Undetermined	Undetermined	Undetermined	Undetermined	Undetermined
28	Undetermined	Undetermined	Undetermined	Undetermined	Undetermined	Undetermined	Undetermined	Undetermined
29	Undetermined	Undetermined	Undetermined	Undetermined	Undetermined	Undetermined	Undetermined	Undetermined
30	Undetermined	Undetermined	Undetermined	Undetermined	Undetermined	Undetermined	Undetermined	Undetermined
31	Undetermined	Undetermined	Undetermined	Undetermined	Undetermined	Undetermined	Undetermined	Undetermined
32	Undetermined	Undetermined	Undetermined	Undetermined	Undetermined	Undetermined	Undetermined	Undetermined
33	Undetermined	22.6	Undetermined	Undetermined	Undetermined	Undetermined	Undetermined	28.6
34	Undetermined	Undetermined	Undetermined	Undetermined	Undetermined	Undetermined	Undetermined	Undetermined
35	Undetermined	Undetermined	Undetermined	Undetermined	Undetermined	Undetermined	Undetermined	Undetermined
36	Undetermined	Undetermined	Undetermined	Undetermined	Undetermined	Undetermined	Undetermined	Undetermined
37	Undetermined	Undetermined	Undetermined	Undetermined	Undetermined	Undetermined	Undetermined	Undetermined
38	Undetermined	Undetermined	Undetermined	Undetermined	Undetermined	Undetermined	Undetermined	Undetermined
39	Undetermined	Undetermined	Undetermined	Undetermined	Undetermined	Undetermined	Undetermined	Undetermined
40	Undetermined	Undetermined	Undetermined	Undetermined	Undetermined	Undetermined	Undetermined	Undetermined
41	Undetermined	Undetermined	Undetermined	Undetermined	Undetermined	Undetermined	Undetermined	Undetermined
42	Undetermined	Undetermined	Undetermined	Undetermined	Undetermined	Undetermined	Undetermined	Undetermined
43	Undetermined	Undetermined	Undetermined	Undetermined	Undetermined	Undetermined	Undetermined	Undetermined
44	Undetermined	Undetermined	Undetermined	Undetermined	Undetermined	Undetermined	Undetermined	Undetermined
45	Undetermined	Undetermined	Undetermined	Undetermined	Undetermined	Undetermined	Undetermined	Undetermined
46	Undetermined	Undetermined	Undetermined	Undetermined	Undetermined	Undetermined	Undetermined	Undetermined
47	Undetermined	Undetermined	Undetermined	Undetermined	Undetermined	Undetermined	Undetermined	Undetermined
48	Undetermined	Undetermined	Undetermined	Undetermined	Undetermined	Undetermined	Undetermined	Undetermined
49	Undetermined	Undetermined	Undetermined	Undetermined	Undetermined	Undetermined	Undetermined	Undetermined
50	Undetermined	Undetermined	Undetermined	Undetermined	Undetermined	Undetermined	Undetermined	Undetermined
51	Undetermined	Undetermined	Undetermined	Undetermined	Undetermined	Undetermined	Undetermined	Undetermined
52	Undetermined	Undetermined	Undetermined	Undetermined	Undetermined	Undetermined	Undetermined	Undetermined
53	Undetermined	Undetermined	Undetermined	Undetermined	Undetermined	Undetermined	Undetermined	Undetermined
54	Undetermined	Undetermined	Undetermined	Undetermined	Undetermined	Undetermined	Undetermined	Undetermined
55	Undetermined	Undetermined	Undetermined	Undetermined	Undetermined	Undetermined	Undetermined	Undetermined

56	Undetermined	Undetermined	Undetermined	Undetermined	Undetermined	Undetermined	Undetermined	Undetermined
57	19.8	19.2	20.8	19.6	15.6	17.9	15.2	18.0
58	Undetermined	Undetermined	Undetermined	Undetermined	Undetermined	Undetermined	Undetermined	Undetermined
59	Undetermined	Undetermined	Undetermined	Undetermined	Undetermined	Undetermined	Undetermined	Undetermined
60	Undetermined	Undetermined	Undetermined	Undetermined	Undetermined	Undetermined	Undetermined	Undetermined
61	Undetermined	Undetermined	Undetermined	Undetermined	Undetermined	Undetermined	Undetermined	Undetermined
62	Undetermined	Undetermined	Undetermined	Undetermined	Undetermined	Undetermined	Undetermined	Undetermined
63	18.5	17.7	19.1	18.3	14.4	16.4	14.9	15.9
64	Undetermined	Undetermined	Undetermined	Undetermined	Undetermined	Undetermined	Undetermined	Undetermined
65	Undetermined	Undetermined	Undetermined	Undetermined	Undetermined	Undetermined	Undetermined	Undetermined
66	Undetermined	Undetermined	Undetermined	Undetermined	Undetermined	Undetermined	Undetermined	Undetermined
67	34.7	33.8	37.0	34.2	32.6	33.3	32.1	32.7
68	Undetermined	Undetermined	Undetermined	Undetermined	Undetermined	Undetermined	Undetermined	Undetermined
69	Undetermined	Undetermined	Undetermined	Undetermined	Undetermined	33.5	Undetermined	35.6
70	11.7	11.4	12.3	10.9	7.8	9.2	9.3	10.4
71	18.3	17.2	17.7	16.9	14.1	15.5	15.0	14.3
72	Undetermined	Undetermined	Undetermined	Undetermined	Undetermined	Undetermined	Undetermined	Undetermined
73	Undetermined	Undetermined	Undetermined	Undetermined	Undetermined	Undetermined	Undetermined	Undetermined
74	Undetermined	Undetermined	Undetermined	Undetermined	Undetermined	Undetermined	Undetermined	Undetermined
75	Undetermined	Undetermined	Undetermined	Undetermined	Undetermined	Undetermined	Undetermined	Undetermined
76	Undetermined	Undetermined	Undetermined	Undetermined	Undetermined	Undetermined	Undetermined	Undetermined
77	Undetermined	Undetermined	Undetermined	Undetermined	Undetermined	Undetermined	Undetermined	Undetermined
78	Undetermined	Undetermined	Undetermined	Undetermined	Undetermined	Undetermined	Undetermined	Undetermined
79	18.0	17.2	19.8	16.9	14.5	14.2	14.3	14.3
80	Undetermined	Undetermined	Undetermined	Undetermined	Undetermined	Undetermined	Undetermined	Undetermined
81	Undetermined	Undetermined	34.2	30.8	Undetermined	Undetermined	Undetermined	Undetermined
82	Undetermined	Undetermined	Undetermined	Undetermined	Undetermined	Undetermined	Undetermined	Undetermined
83	12.6	11.8	12.5	11.7	9.0	10.3	8.5	9.8
84	Undetermined	33.4	Undetermined	Undetermined	Undetermined	32.5	31.9	36.4
85	15.1	13.7	15.7	13.7	10.4	10.4	9.9	10.4

86	Undetermined	Undetermined	Undetermined	Undetermined	Undetermined	Undetermined	Undetermined	Undetermined
87	Undetermined	Undetermined	Undetermined	Undetermined	Undetermined	Undetermined	Undetermined	Undetermined
88	Undetermined	Undetermined	Undetermined	Undetermined	Undetermined	Undetermined	Undetermined	Undetermined
89	19.5	18.4	21.8	20.4	14.5	15.2	14.7	15.8
90	Undetermined	Undetermined	Undetermined	Undetermined	Undetermined	Undetermined	Undetermined	Undetermined
91	Undetermined	Undetermined	Undetermined	Undetermined	Undetermined	Undetermined	Undetermined	Undetermined
92	Undetermined	Undetermined	Undetermined	Undetermined	Undetermined	Undetermined	Undetermined	Undetermined
93	Undetermined	Undetermined	Undetermined	Undetermined	Undetermined	Undetermined	Undetermined	Undetermined
94	Undetermined	Undetermined	Undetermined	Undetermined	Undetermined	34.9	Undetermined	Undetermined
95	Undetermined	Undetermined	Undetermined	Undetermined	Undetermined	Undetermined	Undetermined	Undetermined
96	Undetermined	Undetermined	Undetermined	Undetermined	Undetermined	Undetermined	Undetermined	Undetermined
97	Undetermined	Undetermined	Undetermined	Undetermined	Undetermined	Undetermined	Undetermined	Undetermined
98	Undetermined	Undetermined	Undetermined	Undetermined	Undetermined	Undetermined	Undetermined	Undetermined
99	Undetermined	Undetermined	Undetermined	Undetermined	Undetermined	Undetermined	Undetermined	35.9
100	Undetermined	Undetermined	Undetermined	Undetermined	Undetermined	Undetermined	Undetermined	Undetermined
101	Undetermined	Undetermined	Undetermined	Undetermined	Undetermined	Undetermined	Undetermined	Undetermined
102	Undetermined	Undetermined	Undetermined	Undetermined	Undetermined	Undetermined	Undetermined	Undetermined
103	Undetermined	Undetermined	Undetermined	Undetermined	Undetermined	Undetermined	Undetermined	Undetermined
104	13.9	13.4	16.6	15.6	9.9	11.9	10.2	11.9
105	Undetermined	Undetermined	Undetermined	Undetermined	Undetermined	Undetermined	Undetermined	Undetermined
106	Undetermined	Undetermined	Undetermined	Undetermined	Undetermined	Undetermined	Undetermined	Undetermined
107	Undetermined	Undetermined	Undetermined	Undetermined	Undetermined	Undetermined	Undetermined	Undetermined
108	Undetermined	Undetermined	Undetermined	Undetermined	Undetermined	Undetermined	Undetermined	Undetermined
109	Undetermined	Undetermined	Undetermined	Undetermined	Undetermined	Undetermined	Undetermined	Undetermined
110	Undetermined	Undetermined	Undetermined	Undetermined	Undetermined	Undetermined	35.2	37.1
111	Undetermined	Undetermined	Undetermined	34.7	Undetermined	Undetermined	Undetermined	Undetermined
112	Undetermined	Undetermined	Undetermined	Undetermined	Undetermined	Undetermined	Undetermined	Undetermined
113	Undetermined	Undetermined	Undetermined	Undetermined	Undetermined	Undetermined	Undetermined	Undetermined
114	Undetermined	Undetermined	Undetermined	Undetermined	Undetermined	Undetermined	Undetermined	Undetermined
115	Undetermined	Undetermined	Undetermined	Undetermined	Undetermined	Undetermined	Undetermined	Undetermined

116	Undetermined	Undetermined	Undetermined	Undetermined	Undetermined	Undetermined	Undetermined	Undetermined
117	Undetermined	Undetermined	Undetermined	Undetermined	Undetermined	Undetermined	Undetermined	Undetermined
118	Undetermined	Undetermined	36.2	Undetermined	Undetermined	36.6	Undetermined	Undetermined
119	Undetermined	Undetermined	Undetermined	Undetermined	Undetermined	Undetermined	Undetermined	Undetermined
120	Undetermined	Undetermined	Undetermined	Undetermined	Undetermined	Undetermined	Undetermined	Undetermined
121	Undetermined	Undetermined	Undetermined	Undetermined	Undetermined	Undetermined	Undetermined	Undetermined
122	Undetermined	Undetermined	Undetermined	Undetermined	Undetermined	Undetermined	Undetermined	Undetermined
123	13.2	12.5	15.2	13.1	9.8	12.3	10.3	11.3
124	Undetermined	Undetermined	Undetermined	Undetermined	Undetermined	Undetermined	Undetermined	Undetermined
125	Undetermined	Undetermined	Undetermined	Undetermined	Undetermined	Undetermined	Undetermined	Undetermined
126	19.3	16.7	19.2	17.3	15.1	15.6	15.4	16.0
127	13.1	12.8	13.0	11.9	8.8	9.7	8.9	10.8
128	Undetermined	Undetermined	Undetermined	Undetermined	Undetermined	Undetermined	Undetermined	Undetermined
129	Undetermined	Undetermined	Undetermined	Undetermined	Undetermined	Undetermined	Undetermined	Undetermined
130	18.8	18.3	19.2	18.7	11.3	12.1	12.4	11.2
131	Undetermined	Undetermined	Undetermined	34.3	Undetermined	33.8	Undetermined	Undetermined
132	34.4	30.5	31.1	27.8	31.6	29.9	26.6	26.8
133	Undetermined	Undetermined	Undetermined	Undetermined	Undetermined	Undetermined	Undetermined	Undetermined
134	Undetermined	Undetermined	Undetermined	Undetermined	Undetermined	Undetermined	Undetermined	Undetermined
135	Undetermined	Undetermined	Undetermined	Undetermined	Undetermined	Undetermined	Undetermined	Undetermined
136	Undetermined	Undetermined	Undetermined	Undetermined	Undetermined	Undetermined	Undetermined	Undetermined
137	Undetermined	Undetermined	Undetermined	Undetermined	Undetermined	Undetermined	Undetermined	Undetermined
138	Undetermined	Undetermined	Undetermined	Undetermined	Undetermined	Undetermined	Undetermined	Undetermined
139	Undetermined	Undetermined	Undetermined	Undetermined	Undetermined	Undetermined	Undetermined	Undetermined
140	Undetermined	Undetermined	Undetermined	Undetermined	Undetermined	Undetermined	Undetermined	Undetermined
141	Undetermined	Undetermined	Undetermined	Undetermined	Undetermined	Undetermined	Undetermined	Undetermined
142	Undetermined	Undetermined	Undetermined	Undetermined	Undetermined	Undetermined	Undetermined	Undetermined
143	Undetermined	Undetermined	Undetermined	Undetermined	Undetermined	Undetermined	Undetermined	Undetermined
144	Undetermined	Undetermined	Undetermined	Undetermined	Undetermined	Undetermined	Undetermined	Undetermined
145	Undetermined	Undetermined	Undetermined	Undetermined	Undetermined	Undetermined	Undetermined	Undetermined

146	Undetermined	Undetermined	Undetermined	Undetermined	Undetermined	Undetermined	Undetermined	Undetermined
147	Undetermined	Undetermined	Undetermined	Undetermined	Undetermined	Undetermined	Undetermined	Undetermined
148	Undetermined	Undetermined	Undetermined	34.7	Undetermined	Undetermined	Undetermined	Undetermined
149	Undetermined	Undetermined	Undetermined	Undetermined	Undetermined	Undetermined	Undetermined	Undetermined
150	Undetermined	Undetermined	Undetermined	Undetermined	Undetermined	Undetermined	Undetermined	Undetermined
151	Undetermined	Undetermined	Undetermined	Undetermined	Undetermined	Undetermined	Undetermined	Undetermined
152	Undetermined	Undetermined	Undetermined	Undetermined	Undetermined	Undetermined	Undetermined	Undetermined
153	Undetermined	Undetermined	Undetermined	Undetermined	Undetermined	Undetermined	Undetermined	Undetermined
154	Undetermined	Undetermined	Undetermined	Undetermined	Undetermined	Undetermined	Undetermined	Undetermined
155	Undetermined	Undetermined	31.5	37.0	Undetermined	Undetermined	Undetermined	Undetermined
156	Undetermined	Undetermined	Undetermined	Undetermined	Undetermined	Undetermined	Undetermined	Undetermined
157	Undetermined	Undetermined	Undetermined	Undetermined	Undetermined	Undetermined	Undetermined	Undetermined
158	Undetermined	Undetermined	20.0	19.0	Undetermined	Undetermined	Undetermined	Undetermined
159	Undetermined	Undetermined	Undetermined	Undetermined	Undetermined	Undetermined	Undetermined	Undetermined
160	Undetermined	Undetermined	Undetermined	Undetermined	Undetermined	Undetermined	Undetermined	Undetermined
161	Undetermined	Undetermined	Undetermined	Undetermined	Undetermined	Undetermined	Undetermined	Undetermined
162	Undetermined	Undetermined	Undetermined	Undetermined	Undetermined	Undetermined	Undetermined	Undetermined
163	Undetermined	Undetermined	Undetermined	Undetermined	Undetermined	Undetermined	Undetermined	Undetermined
164	Undetermined	Undetermined	Undetermined	Undetermined	Undetermined	Undetermined	Undetermined	Undetermined
165	Undetermined	Undetermined	Undetermined	Undetermined	Undetermined	Undetermined	Undetermined	Undetermined
166	Undetermined	Undetermined	Undetermined	Undetermined	Undetermined	Undetermined	Undetermined	Undetermined
167	Undetermined	Undetermined	Undetermined	Undetermined	Undetermined	Undetermined	Undetermined	Undetermined
168	Undetermined	Undetermined	Undetermined	Undetermined	Undetermined	Undetermined	Undetermined	Undetermined
169	Undetermined	Undetermined	31.2	28.6	Undetermined	Undetermined	32.4	33.5
170	Undetermined	Undetermined	Undetermined	Undetermined	Undetermined	Undetermined	Undetermined	Undetermined
171	Undetermined	Undetermined	Undetermined	Undetermined	Undetermined	Undetermined	Undetermined	Undetermined
172	Undetermined	Undetermined	Undetermined	Undetermined	Undetermined	Undetermined	Undetermined	Undetermined
173	Undetermined	Undetermined	33.8	30.2	Undetermined	Undetermined	Undetermined	Undetermined
174	Undetermined	Undetermined	Undetermined	Undetermined	Undetermined	Undetermined	Undetermined	Undetermined
175	Undetermined	Undetermined	Undetermined	Undetermined	Undetermined	Undetermined	Undetermined	Undetermined

176	Undetermined	Undetermined	Undetermined	Undetermined	Undetermined	Undetermined	Undetermined	Undetermined
177	Undetermined	Undetermined	Undetermined	Undetermined	Undetermined	Undetermined	Undetermined	Undetermined
178	Undetermined	Undetermined	Undetermined	Undetermined	Undetermined	Undetermined	Undetermined	Undetermined
179	Undetermined	Undetermined	Undetermined	Undetermined	Undetermined	Undetermined	Undetermined	Undetermined
180	Undetermined	Undetermined	Undetermined	Undetermined	Undetermined	Undetermined	Undetermined	Undetermined
181	Undetermined	Undetermined	Undetermined	Undetermined	Undetermined	Undetermined	Undetermined	Undetermined
182	Undetermined	Undetermined	Undetermined	Undetermined	Undetermined	Undetermined	Undetermined	Undetermined
183	19.0	18.5	22.9	21.8	14.9	15.4	14.7	15.7
184	Undetermined	Undetermined	Undetermined	Undetermined	Undetermined	Undetermined	Undetermined	Undetermined
185	Undetermined	Undetermined	Undetermined	Undetermined	Undetermined	Undetermined	Undetermined	Undetermined
186	Undetermined	Undetermined	Undetermined	Undetermined	Undetermined	Undetermined	Undetermined	Undetermined
187	Undetermined	Undetermined	Undetermined	Undetermined	Undetermined	Undetermined	Undetermined	Undetermined
188	Undetermined	Undetermined	Undetermined	Undetermined	Undetermined	Undetermined	Undetermined	Undetermined
189	Undetermined	Undetermined	Undetermined	Undetermined	Undetermined	Undetermined	Undetermined	Undetermined
190	Undetermined	Undetermined	Undetermined	Undetermined	Undetermined	Undetermined	Undetermined	Undetermined
191	Undetermined	Undetermined	Undetermined	Undetermined	Undetermined	Undetermined	Undetermined	Undetermined
192	Undetermined	Undetermined	Undetermined	Undetermined	Undetermined	Undetermined	Undetermined	Undetermined
193	Undetermined	Undetermined	Undetermined	Undetermined	Undetermined	Undetermined	Undetermined	Undetermined
194	Undetermined	Undetermined	Undetermined	Undetermined	33.9	32.9	32.2	34.1
195	Undetermined	Undetermined	Undetermined	Undetermined	Undetermined	Undetermined	Undetermined	Undetermined
196	Undetermined	Undetermined	Undetermined	Undetermined	Undetermined	Undetermined	Undetermined	Undetermined
197	Undetermined	Undetermined	Undetermined	Undetermined	Undetermined	Undetermined	Undetermined	Undetermined
198	Undetermined	Undetermined	Undetermined	Undetermined	Undetermined	Undetermined	Undetermined	Undetermined
199	Undetermined	Undetermined	Undetermined	Undetermined	Undetermined	Undetermined	Undetermined	Undetermined
200	Undetermined	Undetermined	Undetermined	Undetermined	Undetermined	Undetermined	Undetermined	Undetermined

Appendix A8: Control Proposal

Purpose

The purpose of this proposal is to clearly outline the proposed changes to the current DeWorm3 Assay, specifically with respect to the controls included and how they are analyzed. This proposal will detail each control, the current standard, the new proposal, and the data and evidence to support the new proposal.

Internal Process Control: *Bacillus atrophaeus*

Current

The DeWorm3 Assay *B. atrophaeus* control is spiked into each sample at the time of extraction and serves as an internal process control. The *B. atrophaeus* Ct cut off is defined as 2 standard deviations from the mean Ct defined in the validation report (Ct $\leq$ 34, in the absence of STH amplification). Per the validation report, the official Ct score range for the whole of the DeWorm3 study will be defined by 2 standard deviations from all baseline samples.

B. atrophaeus Proposal

In the interest of solidifying a procedure for determining the *B. atrophaeus* Ct score range, the following proposal has been formulated:

(i) Use the 6,133 LMC baseline samples to define the mean, median, and standard deviation.

(ii) Quantigen to re-extract all samples with *B. atrophaeus* Ct Scores that fall outside of 2 standard deviations from the mean, determined in (i), in one or both assays/wells.

(iii) Comparative analysis of STH-status flips at 2, 3, and 4 standard deviations

(a) This will allow a standard to be set for the whole study, and subsequent studies in the field, as to how to evaluate Ct cut offs for an internal process control.

Finally, for a sample to have official results, *B. atrophaeus* or an STH target must amplify within the defined range in both sample wells. All samples that are queued for repeat based on *B. atrophaeus* status will be repeated at the completion of initial testing.

Data in support of B. atrophaeus Proposal

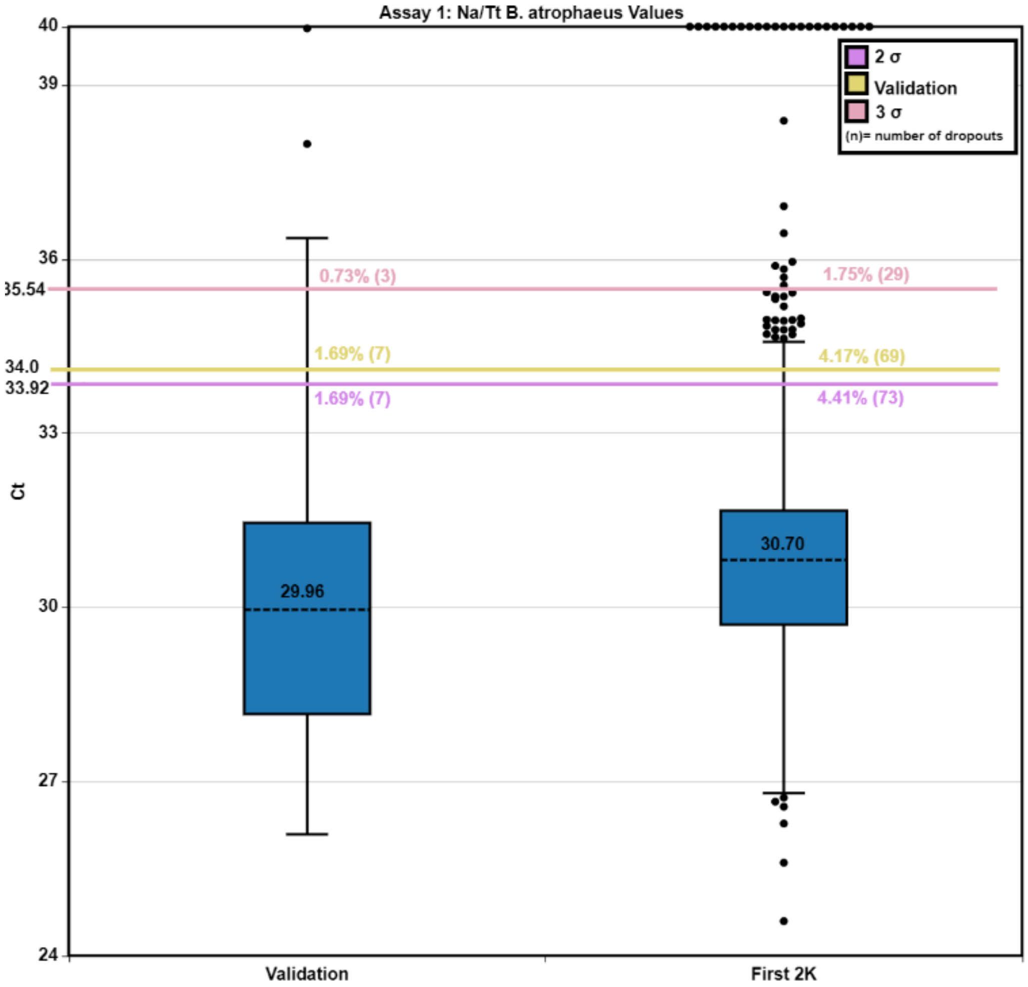

Figure A8.1 Assay 1 (Na/Tt) B. atrophaeus data from Validation and the First 2k, indicating repeats based on Validation cut off, 2 and 3 standard deviation cut offs (from the mean of the first 2,000 samples).

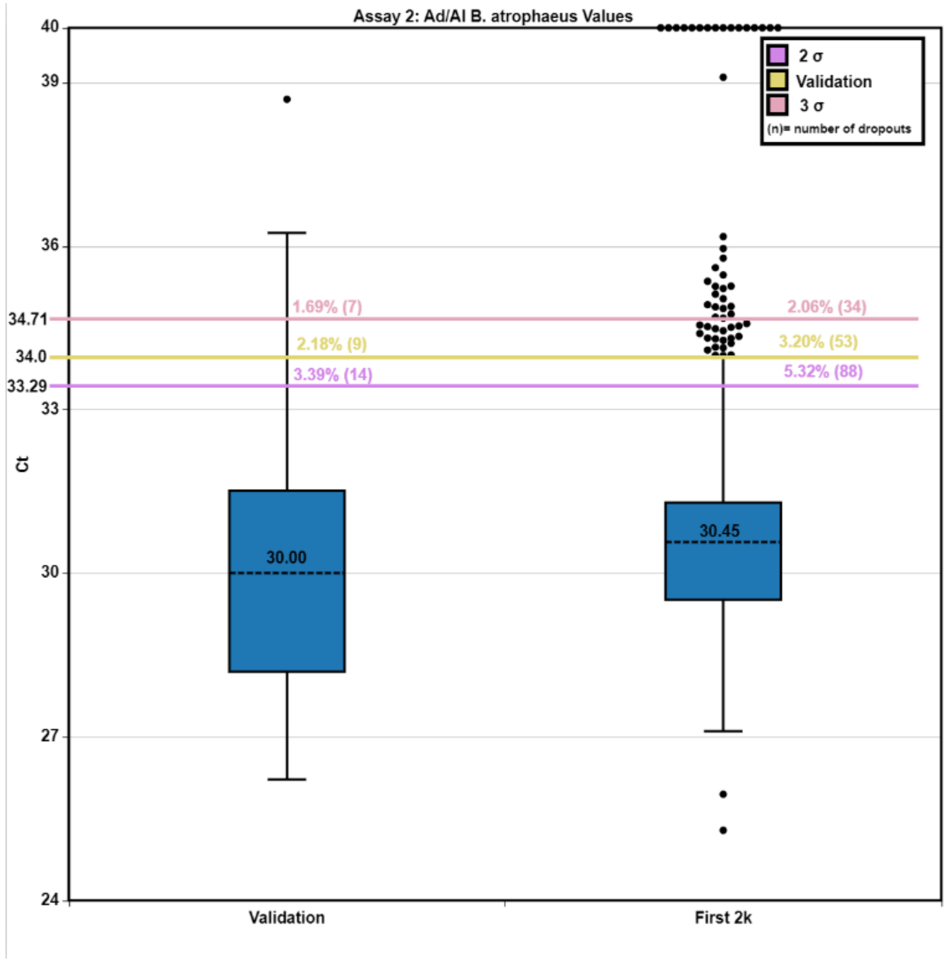

Figure A8.2: Assay 2 (Ad/Al) B. atrophaeus data from Validation and the First 2k, indicating repeats based on the Validation cut off, 2 and 3 standard deviation cut offs (from the mean of the first 2,000 samples).

Table A8.3: Sample Repeats by Bacillus Cut Offs

Bacillus Cut Off Breakdown			
	**Well**	**Extraction**	**Total Failures**
**2 σ**	71	42	**113**
**3 σ**	43	10	**53**
**Validation**	58	32	**90**
**Undetermined**	29	5	**34**

In Figure A8.1 and Figure A8.2, the differences in means (indicated by the dashed black line) between the *B. atrophaeus* scores in Validation (n=413) vs. the First 2,000 (n= 1653) samples can be seen. The

colored lines indicate the Ct score cut offs at Validation (yellow line; $\geq$ 34), 2 standard deviations (in reference to the first 2,000 samples) (purple line; $\geq$ 33.94 Assay 1, $\geq$ 33.28 Assay 2), and 3 standard deviations (pink line; $\geq$ 35.57 Assay 1, $\geq$ 34.68 Assay 2). Table 1. outlines the number of samples which require re-testing due to an unacceptable *B. atrophaeus* Ct score. Of importance, these data clearly show: (1) a shift in *B. atrophaeus* Ct score averages in the First 2k sample data (30.7 ± 1.61 Assay 1, 30.45 ± 1.42 Assay 2) compared to the validation (29.96 ± 2.02 Assay 1, 30.00 ± 2.00 Assay 2), and (2) the drastic drop in repeats when evaluating at a 3 standard deviation cut off (35.54 Assay 1, 34.71 Assay 2), compared to a 2 standard deviation cut off (33.92 Assay 1, 33.29 Assay 2) or the current validation cut off (34).

These data suggest that the means and cut offs for the whole DeWorm3 study will be better informed by all the Benin LMC data. These data also further support the piece of the proposal, outlined in B.II.ii-iii. As there is currently no gold-standard for how to evaluate internal controls in the STH field, the DeWorm3 project with its scope, sits in a unique position to inform the community at which deviation and thus, Ct value cut off is the most appropriate to pass or fail a sample. Evaluating all baseline sample data will set up a firm standard for the DeWorm3 project, the STH scientific community, and save money and resources by reducing the number of frivolous repeats, which occurs when cut offs are decided arbitrarily.

Supporting Material for the B. atrophaeus Proposal

Previous studies from the Williams Lab at Smith College provide evidence for how to determine Ct cut offs to repeat samples. In this study [1], it was found that repeating samples at a 3 standard deviation cut-off was most appropriate. This was decided after evaluating the STH status 'flips' at 2, 3, and 4 standard deviations. While this paper was able to determine this after all samples (n=2,800) were run, this is not feasible for the DeWorm3 project, both in terms of data evaluation and the likely high number of repeats at 2 standard deviations.

Again, this study was not done at the same scale of DeWorm3, but it indicates that setting of a Ct score cut off after all – or in the case of DeWorm3, some – samples have been run, as well as a comparative analysis of STH flips, is the current optimal way deal with internal controls cut offs. It may be important to note that this study used a plasmid-based control (IAC), compared to the DeWorm3 Assay, which uses a spiked bacterial control.

Other studies, again using IAC, did not set a Ct cut-off, but rather repeated samples based on whether the IAC was detected at all or whether it was simply 'Undetermined.' This Paper [2] provides some evidence for not setting a Ct at all, but rather only repeated those samples that show up 'Undetermined.'

These differences are most important when considering the number of repeats across the study. Within the first 2,000 samples, there would be a total of 113 samples repeated, based on 2 standard deviation cut offs; maintaining the Validation cutoff would result in 90 sample repeats. The most compelling data is the drop in the number of samples that are repeated when switching to a 3 standard deviation cut off or a pass/fail cut off. If 3 standard deviations were to be used, then 53 samples would be repeated, while if *B. atrophaeus* was evaluated on a pass/fail basis, only 34 samples would need to be repeated.

Extraction Control: *A. suum*

Current A. suum Control

Currently the DeWorm3 Assay, the *A. suum* control is contrived by spiking eggs into naïve stool. Laid out in the validation plan, the *A. suum* extraction control must have a Ct of $\leq$ 23. An *A. suum* extraction control failure (Ct >23) results in an extraction plate failure.

A. suum Proposal

The new proposal for determining the *A. suum* cut off is as follows:

(i) The Ct cut off for each lot of *A. suum* will be determined after all aliquots from that lot have been ran (~70/lot).[h]

(ii) The Ct cut off will be defined by 2 or 3 standard deviations away from the mean of that lot.[i]

(iii) Plates will be officially passed/failed based on what falls outside of that range.

---

[h] A Batch of 70 was a rough estimate by Dr. Pilotte and supported by Dr. Williams. Both agreed this number, while smaller, will provide the opportunity for better results.

[i] The standard deviation number will be determined upon comparative analyses of these, like the other controls.

Data in support of A. suum Proposal

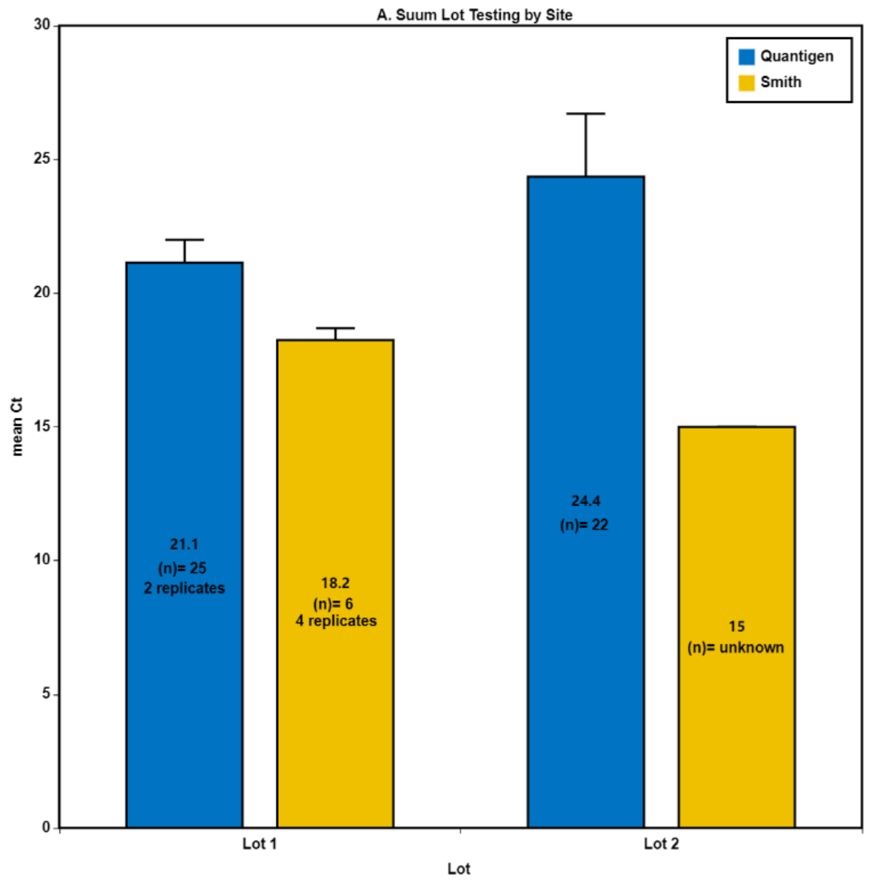

Figure A8.4: Mean comparison between A. suum lots. Results separated by Smith and Quantigen. Smith and Quantigen threshold are different (0.03 and 0.06) and can cause a 3 Ct shift.

Table A8.5: A. suum Lot-to-Lot Variation

A. suum (Pos. Extract)				
Lot #	***1***		***2***	
	**Validation**	**Smith**	**First 2K**	**Smith**
n	*50.0*	*24*	*22*	*x*
**Max**	23.3	18.9	29.8	n/a
**Min**	19.3	17.5	20.7	n/a
**Mean**	21.1	18.3	24.4	15.0
**σ**	0.86	0.44	2.46	n/a

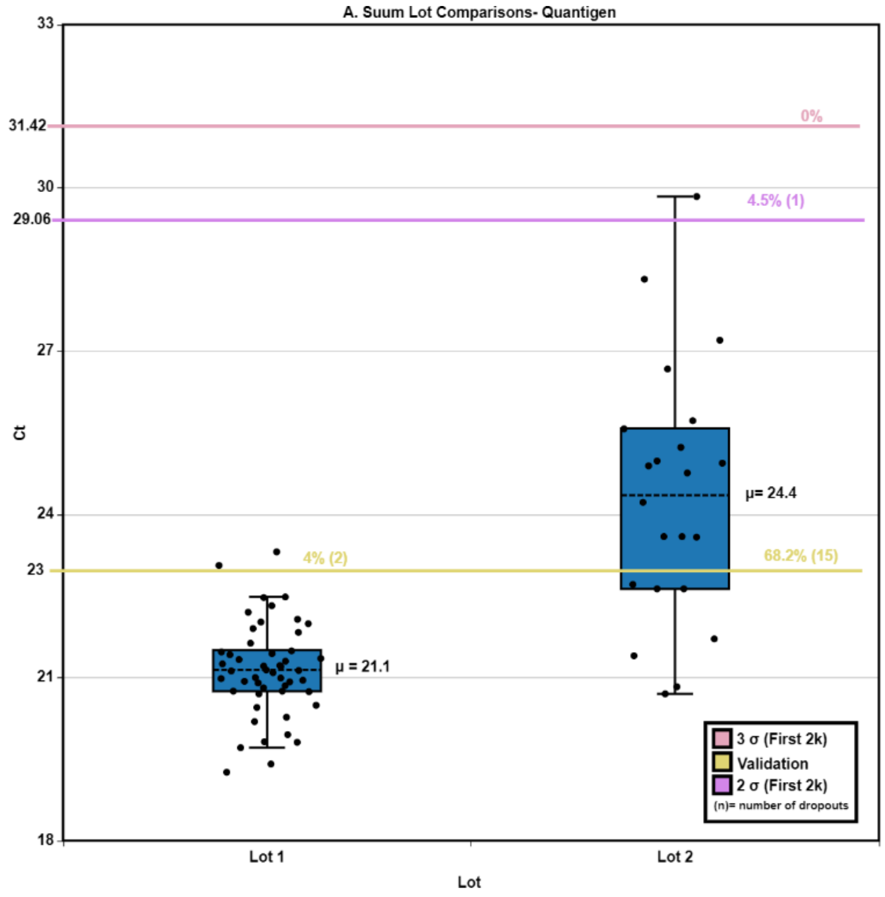

Figure A8.6: A. suum Lot 1 vs. Lot 2 Box Plot (Quantigen Data Only). Includes Cut Offs based on Validation (Ct ≤ 23; yellow line), 2 standard deviations (Lot 2 data Only) (Ct ≤ 29.06; purple line), and 3 standard deviations (Ct ≤ 31.42; orange line)

Table A8.7: First 2k A. Suum Failures

***A. Suum* Cut Off Breakdown (First 2k)**	
	**Total Failures**
**2 σ**	**1**
**3 σ**	**0**
**Validation**	**15**

Looking at all the above figures, the lot-to-lot differences become very apparent. All Validation samples were from Lot 1 while the First 2k samples were from Lot 2. The differences between Quantigen and Smith Ct scores for the *A. suum* are not of concern as the specific testing methods and data analysis is different and have previously shown to produce a shift in Ct scores.

The lot-to-lot differences also speak to the variability of the naïve stool that can be acquired and used. The nature of the sample type and the composition of each stool sample produces differences that further confound this control and dispels the idea that all lots of *A. suum* should be treated the same as the initial Validation lot.

In addition, the control is contrived and created by hand with variable results. Looking at the mean differences between Lots 1 and 2, the mean of Lot 2 is shifted above the Validation Cut Off. This clearly indicates that the mean, range, standard deviation, and cut off that was appropriate for Lot 1 will not be applicable to subsequent lots and will lead to an unsustainable repeat rate.

Further emphasizing this point in Figure 4, there are incredibly high repeat rates (n=15) for the first 22 plates, if the Validation Cut off and previous control standard, is to be followed. Readjusting the data, based on the proposal in C.II, the number of repeat rates drops to 1 with an adjusted 2 standard deviation cut off and drops to 0 with the adjusted 3 standard deviation cut off.

Supporting Material for the A. suum Proposal

An alternative to the proposal at hand could be to introduce rigorous lot testing which would include testing many samples. However, this is not a feasible solution for the contrived control. The amount of work that goes into creating the control limits the number of controls that can be made per batch. Additionally, the number of aliquots that would be required for extensive lot testing, to ensure that a true range is represented, would introduce the need to pool different aliquots of naïve stool, increasing the amount of variability in composition, and subsequently, Ct scores.

Additionally, the lack of lot testing at one site is not a concern in terms of the stability of the control itself. While there is no stability data on this control and egg, previous studies [3] have shown that even if the eggs break, the DNA itself is stable.

While setting a Ct score cut-off for batches after using a full lot of *A. suum* control is not ideal, the fact that this control is contrived and is not being produced in a QC manner must remain as part of the conversation. There is not a strong and proven precedent for using contrived controls in this manner, and there is certainly no evidence supporting using the first lot of a contrived control to set cut-off ranges for subsequent lots. This standard would be appropriate if the controls had been produced through a Quality Control Process, but as this is not possible for the STH community at this time, this is the best alternative.

Negative Controls: No Sample Control and No Template Control

Current Controls

No Sample Control (NSC)

The NSC control is an all-reagent control with no stool added, but with *B. atrophaeus* spiked in. This NSC serves as a negative process control, and the addition of *B. atrophaeus* serves as an extraction control. If the NSC *B. atrophaeus* score falls outside of the determined range (see 0, or any STH is amplified, then this results in an extraction plate failure (DeWorm3 Assay Validation Plan, *I.K. Description, Preparation, and Validation of Run Controls)*.

No Template Control (NTC)

The NTC control contains no stool or *B. atrophaeus* and is added directly to the qPCR plate. This is a PCR control to ensure that there is no contamination. If there is any amplification of STH or *B. atrophaeus*, then the NTC fails which results in a qPCR plate failure.

NSC/NTC Proposal

As there are both an NSC and NTC that serve the function of a contamination control, the general proposal is to eliminate the NTC, making the NSC the primary contamination control on the plate.

The more detailed proposal is as follows:

a) NSC will be "reagent-only," no *B. atrophaeus* spiked in.

b) Will double as an NTC in qPCR

Data to support NSC/NTC Proposal

Across the 22 plates of the first 2000 data, there have been no NSC failures due to STH contamination. Additionally, there have been no NTC failures due to contamination.

Looking at Figure A8.5: Plate Repeats for the first 2k samples based on NSC failures in Assay 1 (Na/Tt), there are various NSC repeats based on *B. atrophaeus* failures in Assay 1 (Na/Tt); 5 when using the Validation and 2 standard deviation cut off, and 1 using the 3 standard deviation cut off. In Figure 6, there are additional NSC repeats based purely on *B. atrophaeus* failures in Assay 2 (Ad/Al); 9 when using 2 standard deviations, 3 when using the Validation, and 1 using 3 standard deviations.

If this were a sample, there would be either 1 sample repeat or 5 samples repeats, but because this is a control that is tied to the status of the whole plate, all 92 samples would be failed and

queued for repeat, thus wasting enormous amounts of resources and time. A visual of these repeats can be seen in Table 4.

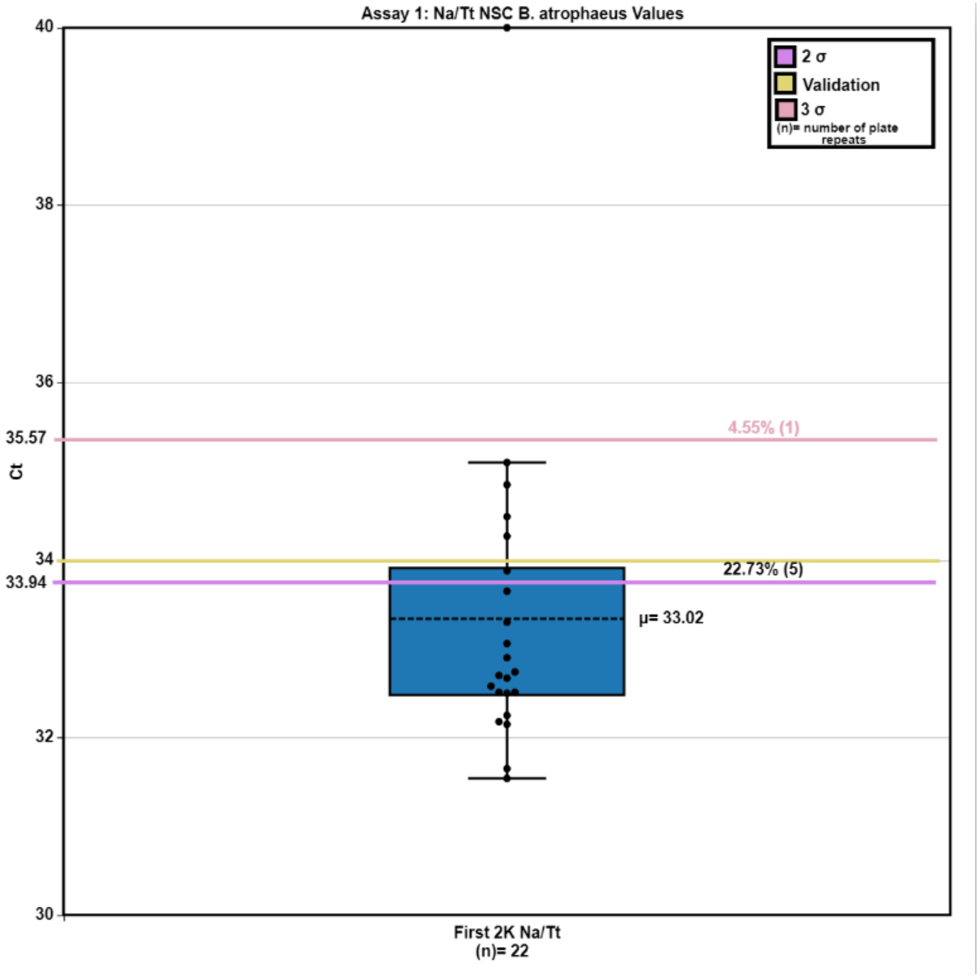

Figure A8.8: Plate Repeats for the first 2k samples based on NSC failures in Assay 1 (Na/Tt)

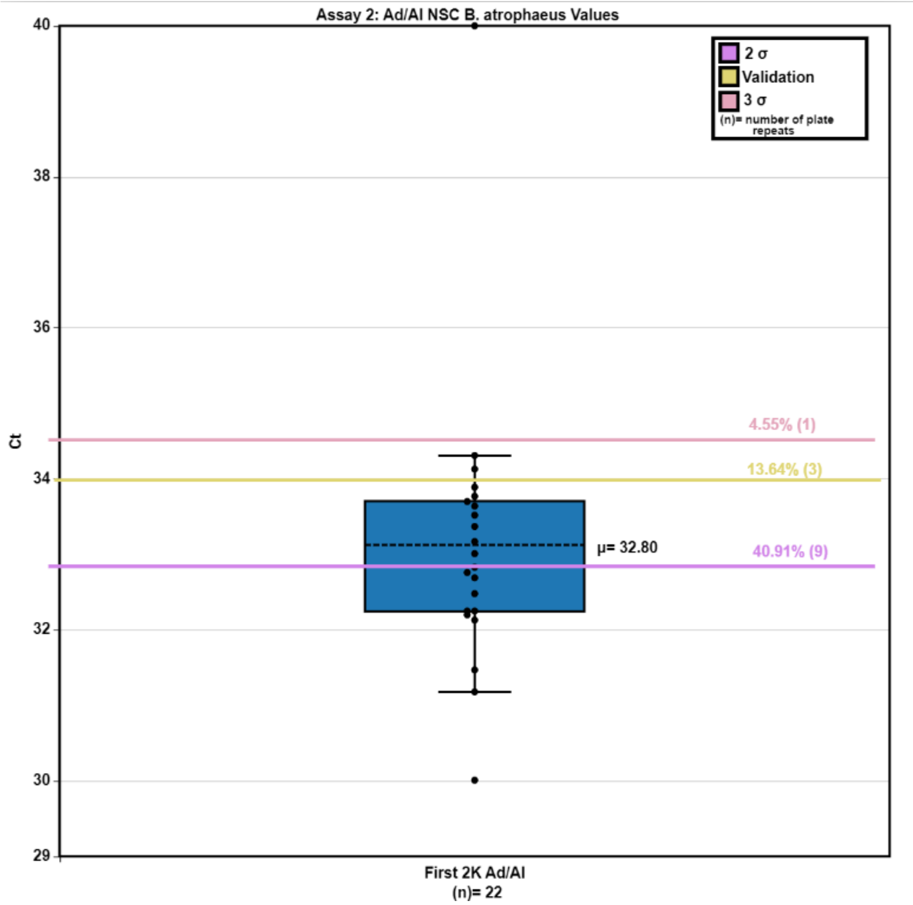

Figure A8.9: Plate Repeats for the first 2k samples based on NSC failures in Assay 2 (Ad/Al)

Table A8.10: Number of Samples Repeated due to NSC B. atrophaeus failures

Number of Sample Repeats from NSC Failures		
	**Plates Failed**	**Samples Queued for Repeat**
**2 σ**	14	1288
**3 σ**	2	184
**Validation**	8	736

To reiterate, all failures indicated in A8.10 are failures due strictly to *B. atrophaeus*. There have not been any NSC failures due to STH amplification.

Evidence to support NSC/NTC Proposal

Other assays do not have both NSC and NTC controls on a plate. As both of these are controls that are intended to monitor contamination, having both are redundant, and as seen in 0, there would be frivolous repeats by including both of these controls. The standard for other developed assays follows this and should be replicated in the DeWorm3 assay.

Furthermore, as the DeWorm3 Assay Plate is lyophilized, the primary opportunity to introduce contaminants (i.e., when the MasterMix is being prepared) has been eliminated. In typical liquid assays, the NTC would test for such contamination, but as the assay is lyophilized this control, again, becomes superfluous and ends up testing for the same contamination that the NSC could.

Positive PCR

Current Positive PCR Standard

Per the Validation Report, the current passing qualification for the DeWorm3 Positive PCR WGA STH Control is within 2 standard deviations from the validation-defined mean.

This control is a contrived control that was pooled and aliquoted out, as to limit any lot-to-lot deviations. Although there is technically only one lot of this control, the cocktail of WGA DNA was created in such a way as to target similar Cts across all the STH species. This creation considered the number of repeat sequences and adjusted the amount of input material to generate a particular Ct score. This concept has led to the creation of a control in which there is less WGA material for *N. americanus* and *A. lumbricoides* than the other targets.

Positive PCR Proposal

Transition to Plasmids

Quantigen proposes that the DeWorm3 Assay moves away from using a WGA cocktail, rather using a cocktail of single copy plasmids per target.

(i) If this proposal is accepted, Quantigen will validate this control to set a 3 standard deviation cut off to pass/fail the batch

(ii) If this proposal is accepted, Quantigen secondarily proposes that there are only two wells of positive PCR per 384 well batch (i.e. only on one of the 96-well plates will there be a reserved spot for positive PCR).

(iii) Quantigen will run the plasmid cocktail at 4 pg/PCR reaction (1 pg of plasmid/target).

Continue with WGA, with altered passing qualifications.

(i) If one of the two control wells amplifies all 4 targets in one batch, the batch will pass regardless of the status of the other Pos. PCR control

(ii) Ct range cut off to be expanded to include Cts seen in validation and First 2k data

(iii) Alternatively, we could create a new batch of WGA gDNA which is not titrated to specific Ct score

Data to Support Positive PCR Proposal

While conducting lot testing on sets of aliquots sent from Smith, *A. lumbricoides* exhibited fall out in 2/3 lots, with high Ct score ranges in 2/3 lots. This is summarized in Table 6 and illustrated in Figure A8.11.

Table A8.12 fall out numbers were calculated by using the validation cut offs. These are as follows: *N. americanus ≤31.6, T. trichiura ≤ 28.8, A. duodenale ≤ 32.7, A. lumbricoides ≤ 35.5.* Within these, there are 4 replicates of *A. lumbricoides* where there was no amplification; these Undetermined scores are distributed across all the lots.

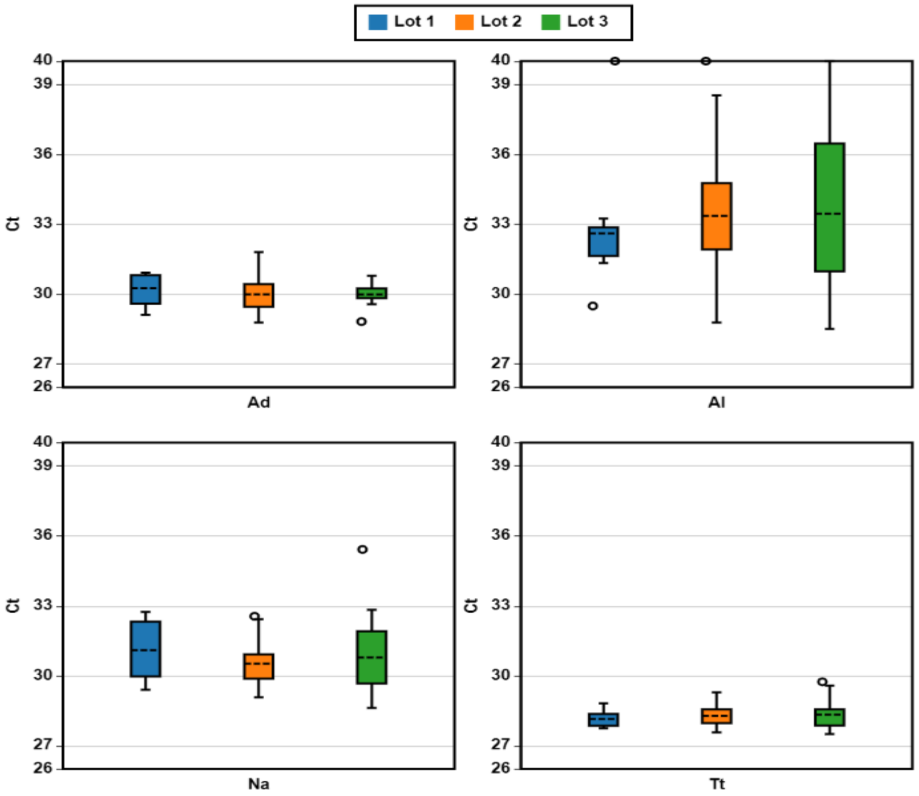

Figure A8.11: WGA STH gDNA Lot Comparisons

Table A8.12: Positive PCR Lot Testing

Comparison of Averages Across New Lots (1:4 Dilution)													
**Lot #**	**Tech. Reps.**	**Na**			**Tt**			**Ad**			**Al**		
		Avg.	*σ*	*# Fall Out*	*Avg.*	*σ*	*# Fall Out*	*Avg.*	*σ*	*# Fall Out*	*Avg.*	*σ*	*# Fall Out*
**Lot 1**	**12**	**31.10**	1.19	4	**28.14**	0.33	1	**30.26**	0.65	0	**32.61**	2.52	1
**Lot 2**	**24**	**30.52**	0.91	3	**28.28**	0.46	4	**29.99**	0.70	0	**33.36**	2.81	4
**Lot 3**	**24**	**30.78**	1.61	7	**28.33**	0.57	4	**29.98**	0.40	0	**33.45**	3.42	7
**All Lots**		**30.80**	1.28	**14**	**28.25**	0.49	**9**	**30.08**	0.59	**0**	**33.14**	2.98	**12**

From these data, the unreliability of the current Positive PCR standard is seen. When the control performs well, the averages for each target are consistent, however, the positive PCR control should not allow for any fall out from the range.

In Table A8.12, it is clearly seen there is pervasive fall out across 3 of 4 targets, with each instance of fall out triggering a qPCR repeat.

A transition to plasmids will provide a more controlled spike-in quantity, in addition to increased confidence in the amount of each target that is spiked in.

If there is the desire to maintain use of WGA gDNA, considering this data, it can be clearly seen that there would be frivolous batch-wide repeats, due to the contrived, titrated nature of the controls. It is believed that this could be ameliorated if the control is no longer titrated.

It is also important to note, that while there was no fall out across the first 11 batches, all these batches were testing with Lot 1 of Positive PCR, which had much tighter ranges for all targets.

Evidence to Support Positive PCR Proposal

Other assays that have been developed for testing of STH species have used plasmids as the positive PCR control. By using a plasmid as a positive PCR control, it would more closely align with the current standard within the field.

While there has been concerns about increased contamination when using plasmids, but this contamination concern is only present when the plasmids are growing. This can be alleviated by only growing plasmids at Smith or Quantigen, and then exporting the plasmid DNA to the other sites for testing.

Reference

1. Benjamin-Chung J, Pilotte N, Ercumen A, Grant JR, Maasch JRMA, et al. (2020) Comparison of multi-parallel qPCR and double-slide Kato-Katz for detection of soil-transmitted helminth infection among children in rural Bangladesh. PLOS Neglected Tropical Diseases 14(4): e0008087. https://doi.org/10.1371/journal.pntd.0008087

2. Dunn, J.C., Papaiakovou, M., Han, K.T. *et al.* The increased sensitivity of qPCR in comparison to Kato-Katz is required for the accurate assessment of the prevalence of soil-transmitted helminth infection in settings that have received multiple rounds of mass drug administration. *Parasites Vectors* **13,** 324 (2020). https://doi.org/10.1186/s13071-020-04197-w

3. Papaiakovou M, Pilotte N, Baumer B, et al. A comparative analysis of preservation techniques for the optimal molecular detection of hookworm DNA in a human fecal specimen. *PLoS Negl Trop Dis*. 2018;12(1):e0006130. Published 2018 Jan 18. doi:10.1371/journal.pntd.0006130
