# Supplementary figures and images for: Development and validation of a high-throughput qPCR platform for the detection of soil-transmitted helminth infections
Source: PLoS Negl Trop Dis. 2025 Jan 10;19(1):e0012760. doi: 10.1371/journal.pntd.0012760 (PMC11756772; doi:10.1371/journal.pntd.0012760)

**S1 Text. Assay Accuracy at the Technical Replicate and Individual Extraction Level**


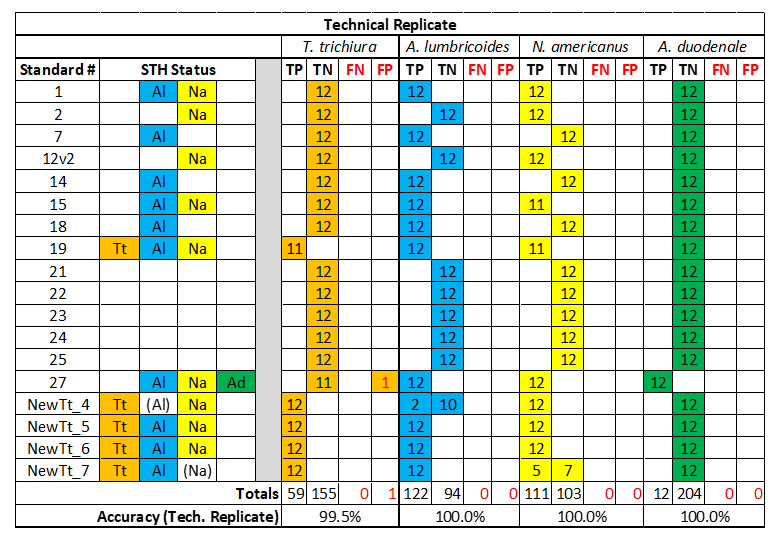


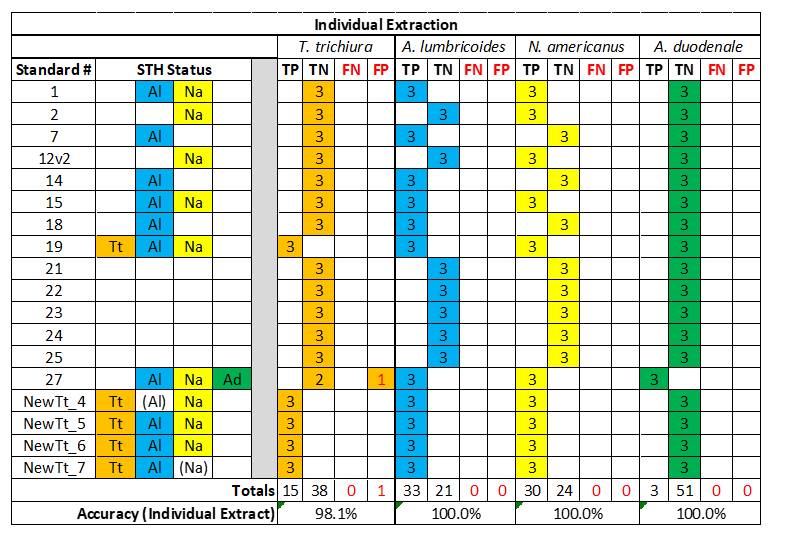

Supplement: S1 Text — Performance metrics were determined using contrived samples containing various combinations of target species. (DOCX) [file pntd.0012760.s008.docx]
